# An Allele of *Glutamate Formiminotransferase* Triggers 5‐Methyl‐Tetrahydrofolate‐to‐MeFox Conversion and Facilitates Folate Biofortification in Maize

**DOI:** 10.1002/advs.202415082

**Published:** 2025-08-21

**Authors:** Tong Lian, Wenzhu Guo, Yanjing Wang, Weiwei Wen, Weixuan Wang, Ling Jiang, Qiuju Liang, Ji'an Liu, Haijun Liu, Yuan Xue, Lixu Pan, Qiaoquan Liu, Ping Yin, Delin Zhang, Jianbing Yan, Chunyi Zhang

**Affiliations:** ^1^ Biotechnology Research Institute Chinese Academy of Agricultural Sciences Beijing 100081 China; ^2^ Sanya Institute Hainan Academy of Agricultural Sciences Sanya 572000 China; ^3^ National Nanfan Research Institute Chinese Academy of Agricultural Sciences Sanya 572000 China; ^4^ Institute of Food Crops Hainan Academy of Agricultural Sciences/Hainan Key Laboratory of Crop Genetics and Breeding Haikou 571100 China; ^5^ National Key Laboratory of Crop Genetic Improvement Huazhong Agricultural University Wuhan 430070 China; ^6^ Key Laboratory of Crop Genomics and Molecular Breeding of Jiangsu Province College of Agriculture Yangzhou University Yangzhou 225009 China

**Keywords:** 5‐methyl‐tetrahydrofolate, folate metabolism, *GFT*, maize, MeFox

## Abstract

Identifying genes involved in folate accumulation is critical for elucidating the regulatory mechanisms of folate metabolism and breeding folate‐rich crops. Here, a natural A‐to‐G variation at the 682nd bp is identified in the coding sequence of an identified plant gene *glutamate formiminotransferase* (*GFT*) in maize, leading to a glycine‐to‐asparagine substitution at the 228th in the protein sequence and contributing to the variation of folate accumulation in mature seeds of a maize inbred line population. This gene encodes a protein highly similar to the *formiminotransferase* domain of mammalian *f ormiminotransferase cyclodeaminase*. In vitro biochemical analysis of this protein reveals an activity of triggering 5‐methyl‐tetrahydrofolate (5‐M‐THF)‐to‐MeFox conversion, other than exerting an activity of *formiminotransferase* in mammals. Loss of *ZmGFT* function triples 5‐M‐THF levels, and overexpression of G‐allele‐carrying *ZmGFT* boosts the metabolic flow toward MeFox. Functional conservation of *GFT* is validated in rice and *Arabidopsis*. The asparagine‐to‐glycine substitution enhances 5‐M‐THF‐to‐MeFox conversion, as demonstrated by in vitro assays and in silico analyses. The functional characterization of the *GFT* gene has uncovered a new metabolic fate of 5‐M‐THF, apart from a C1 donor for methionine synthesis, in plants, and a distinct activity from its mammalian ortholog. The natural variation identified is useful for breeding folate‐fortified maize varieties.

## Graphical abstract

This work demonstrates the distinct roles of the *glutamate formiminotransferase* (*GFT*) enzyme in plants. The *GFT* triggers the conversion of 5‐methyl‐tetrahydrofolate to MeFox in plants, a process distinct from its role as a *formiminotransferase* in mammals. A key allelic variation in *GFT*, with the G‐allele linked to high folate levels in maize, highlights its significant impact on folate metabolism and breeding.

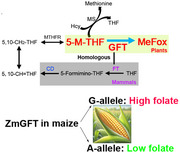

## 1 Introduction

Folates, including tetrahydrofolate (THF) and its derivatives, are essential water‐soluble B‐vitamins for all living organisms. Human body cannot synthesize folates de novo and must capture folates from dietary foods.^[^ ^1^ ^]^ Unfortunately, the dietary intake of folates is inadequate in both developing and developed countries.^[^ ^2^, ^3^, ^4^ ^]^ The worldwide prevalence of neural tube defects, a severe disorder caused by folate deficiency, remains between 0.3 and 124.1 per 10 000 births.^[^ ^5^, ^6^ ^]^ Folate deficiency is considered a global public health problem and prevention of the folate deficiency remains a global priority.

**Figure 1:**
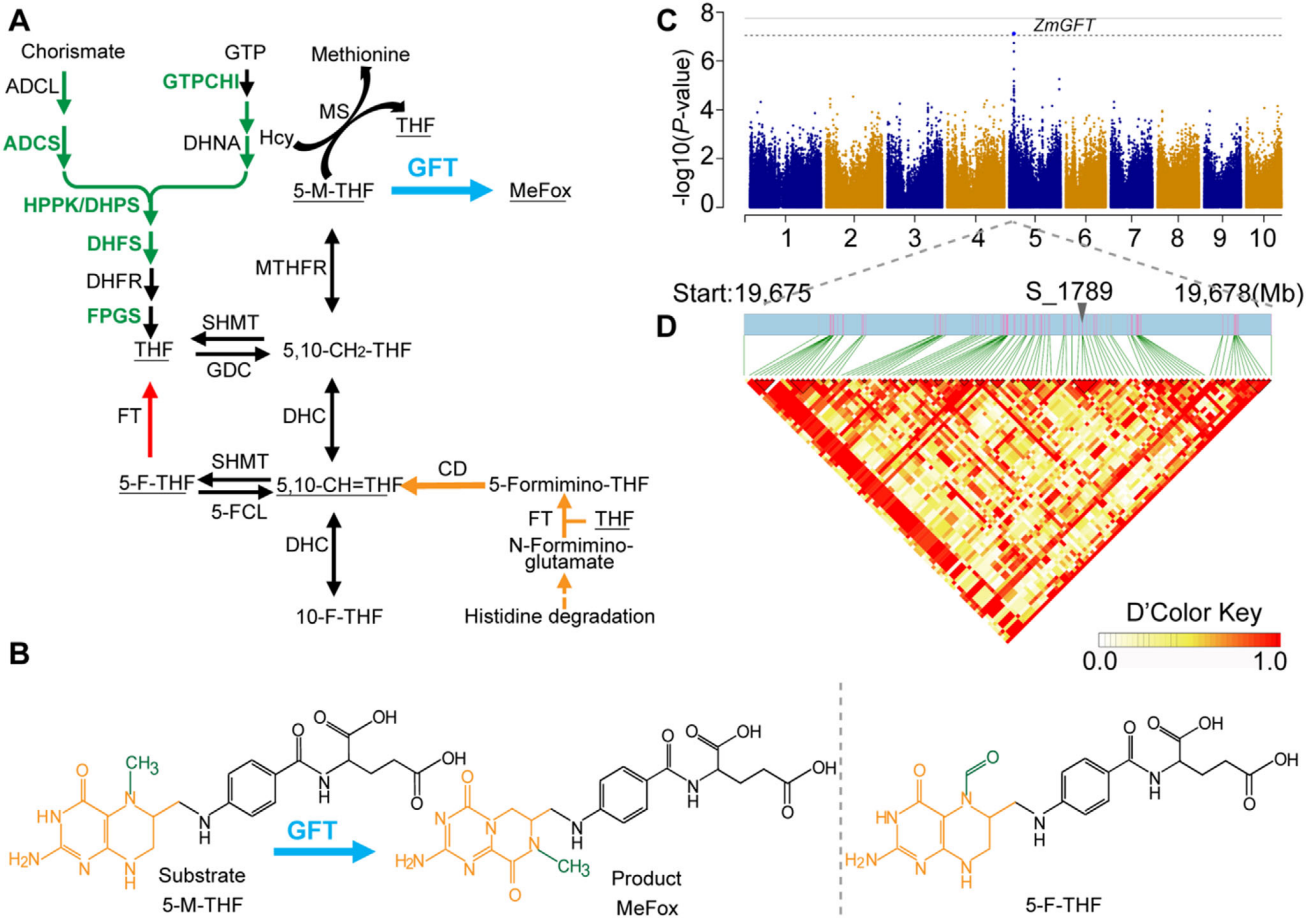
Genome‐wide association study and identification of the major gene locus that underlies folate variation in corn seeds. A) Scheme of folate metabolism. Chemicals: 5‐F‐THF, 5‐formyl‐tetrahydrofolate; 10‐F‐THF, 10‐formyl‐tetrahydrofolate; GTP, guanosine triphosphate; Hcy, homocysteine; MeFox, a pyrazino‐*s*‐triazine derivative of 4α‐hydroxy‐5‐methyl‐tetrahydrofolate; 5‐M‐THF, 5‐methyl‐tetrahydrofolate; 5,10‐CH = THF, 5,10‐methenyl‐tetrahydrofolate; 5,10‐CH_2_‐THF, 5,10‐methylene‐tetrahydrofolate; THF, tetrahydrofolate. The underlined chemicals were described in this report. Enzymes: ADCL, 4‐aminodeoxychorismate lyase; ADCS, 4‐aminodeoxychorismate synthase; *GFT*, conversion from 5‐M‐THF to MeFox; DHC, 5,10‐methylene‐THF dehydrogenase/5,10‐methenyl‐THF cyclohydrolase; DHFR, dihydrofolate reductase; DHFS, dihydrofolate synthase; DHNA, 7,8‐dihydroneopterin aldolase; 5‐FCL, 5‐F‐THF cycloligase; GDC, glycine decarboxylase complex; GTPCHI, GTP cyclohydrolase I; *FTCD*, *glutamate formiminotransferase cyclodeaminase*; FPGS, folylpolyglutamate synthetase; MS, methionine synthase; MTHFR, methylenetetrahydrofolate reductase; SHMT, serine hydroxymethyltransferase. Genes used in biofortification were bold green. Green arrows indicate the plant‐specific reactions. Red arrows indicate the mammalian and bacterial *FT* mediate a reversible formyl transfer between 5‐FCL and THF. Orange arrows indicate the mammal‐specific reactions. Blue arrow indicates the conversion reported in this study. B) Structural formulas of 5‐F‐THF, 5‐M‐THF, and MeFox. The formyl group in 5‐F‐THF and methyl groups in either 5‐M‐THF or MeFox were colored in green. The different structures of 5‐M‐THF or 5‐F‐THF (pteridine part) and MeFox were colored in orange. C) Manhattan plot of GWAS for the content of 5‐M‐THF in mature corn seeds, with the position of *ZmGFT* being indicated. D) Linkage disequilibrium (LD) heatmap of candidate gene *ZmGFT* (chromosome 5: 19.675Mb‐19.678Mb). The *x*‐axis indicates the physical positions of SNP markers within this interval, with the highlighted marker S_1789 denoting a specific SNP of interest. The triangular heatmap below illustrates the pairwise LD (*D'*) values between SNPs in this region, with color intensity representing the strength of LD. The *D'* Color Key to the right shows the gradient from 0.0 (white, low LD) to 1.0 (red, high LD), indicating varying degrees of LD between SNP pairs. High *D'* values in red represent strong LD between loci, suggesting closer genetic linkage.

To alleviate folate deficiency, food fortification and medical supplementation have been implemented in many countries. Folic acid supplementation was proved effective for reducing the prevalence of neural tube and fetal abdominal wall defects,^[^ ^7^, ^8^ ^]^ but led to concerns regarding the potential adverse effects of elevated folate status.^[^ ^9^ ^]^ However, staple crops, including rice (*Oryza sativa*), maize (*Zea mays*), and wheat (*Triticum aestivum*), contain low levels of folates,^[^ ^10^, ^11^, ^12^ ^]^ thus likely leading to a poor folate intake in the population mainly depending on cereal foods.^[^ ^13^ ^]^ To reduce folate shortage in human, biofortification in crops with high levels of folates has been considered cost‐effective, sustainable, and easily accessible,^[^ ^14^, ^15^, ^16^, ^17^ ^]^ making it more acceptable for humans, especially for poor populations. Metabolic engineering of the *para*‐aminobenzoic acid and pterin branch with the trunk of folate biosynthesis, but not the genes involved in one‐carbon metabolism, has been an effective strategy to enhance folate accumulation in crops (**Figure** **1**A).^[^ ^16^, ^17^ ^]^ Consequently, various levels of folate accumulation were achieved in maize (≈160 µg per 100 g), potato (*Solanum tuberosum*, ≈400 µg per 100 g), rice (≈2500 µg per 100 g), tomato (*Solanum lycopersicum*, ≈1000 µg per 100 g), and wheat (≈140 µg per 100 g) through overexpression of the folate biosynthetic genes *GTP cyclohydrolase I* (*GTPCHI*) and *4‐aminodeoxychorismate synthase* (*ADCS*) in combination with enhanced vitamin stability.^[^ ^11^, ^18^, ^19^, ^20^, ^21^ ^]^ Unfortunately, to our knowledge, these genetically modified crops have not yet been commercialized.

Marker‐assisted breeding approaches have also been attempted to increase folate accumulation in crops, but to our knowledge, only were some genetically mapped quantitative trait loci (QTLs)/markers reported, such as the transcriptional association of folate accumulation with *7,8‐dihydroneopterin aldolase* (*DHNA*) and *ADCS* in common beans (*Phaseolus vulgaris*),^[^ ^22^ ^]^ two QTLs in maize,^[^ ^23^ ^]^ and single nucleotide polymorphism (SNP) markers in pea (*Pisum sativum*)^[^ ^24^ ^]^ and potato.^[^ ^25^ ^]^ Potential of these QTLs/markers in folate biofortification awaits further investigation. Hence, there is an urgent need to identify new target genes and allelic variations for breeding folate‐rich crops using molecular‐marker‐assisted selection approach. On one hand, this will further advance our understanding of the regulatory mechanism of plant folate metabolism, and on the other hand, the crop varieties rich in folates can be easily accessed by consumers for their health benefits.

In this study, a genome‐wide association study (GWAS) was conducted using a maize association panel genotyped by more than 1 million genome‐wide SNPs.^[^ ^26^, ^27^ ^]^ Consequently, a gene was cloned and extensively characterized for its biological function in both in vitro assays and in vivo studies in plants. We showed that this gene encodes a previously uncharacterized protein functioning to trigger the conversion of 5‐methyl‐tetrahydrofolate (5‐M‐THF) to MeFox in plants, a pyrazino‐*s*‐triazine derivative of 4α‐hydroxyl‐5‐methy‐THF (Figure 1B), other than to possess a *formiminotransferase* activity as in mammals. Additionally, a natural A‐to‐G variation in this gene was also identified with a potential in genetic breeding for folate‐rich maize varieties.

## 2 Results

### 2.1 GWAS Analyses Identify a Candidate Gene Associated with 5‐M‐THF Accumulation in Corn Seeds

Using a previously developed high‐performance liquid chromatography/mass spectrum (HPLC/MS) method,^[^ ^28^ ^]^ the folate derivative of 5‐M‐THF was profiled in the mature seeds collected from the maize inbred lines of the association panel^[^ ^26^ ^]^ grown in Hainan in 2009 (09ZHN, Table S1, Supporting Information), Yunnan in 2010 (10WY, Table S2, Supporting Information), and Hubei in 2010 (10AMH, Table S3, Supporting Information). The best linear unbiased prediction (BLUP) approach based on a mixed linear model was used for GWAS analysis (Table S4, Supporting Information). The 5‐M‐THF levels ranged from 0.19 to 0.79 nmol g^−1^ and displayed a heritability of 0.42 (Table S5, Supporting Information). We identified some highly linked significant SNPs, including Chr5.S_19676906 (S1789, corresponding to G228N), Chr5.S_19676907 (S1790, corresponding to G228N), Chr5.S_19676887 (S1770, corresponding to G221G), and Chr5.S_19676929 (S1882, corresponding to C235C) which are described in detailed below, associated with 5‐M‐THF content at the level of *p* ≤ 6.93 × 10^−8^ (Figure 1C,D, and Table S6 (Supporting Information)). All the association signals were mapped to a locus on chromosome 5 (GRMZM2G124863), encoding a protein that triggers the conversion of 5‐M‐THF to MeFox (described in detail below). It was reported that its orthologs in algae and land plants are initially named as *glutamate formiminotransferase* (*GFT*), possessing a single *formiminotransferase* domain and a well‐conserved motif pattern.^[^ ^29^ ^]^ The *ZmGFT* gene consists of two exons, and is annotated to be a folic‐acid‐binding protein of 326 amino acids (www.maizegdb.org). The protein sequence alignment and structure analysis of *GFT* orthologs from plants and mammals were performed to identify the sequence differences and domain variations between them (Figure S1, Supporting Information).

### 2.2 *GFT* Has an Impact on Folate Derivative Accumulation in Maize

To investigate whether *ZmGFT* is responsible for the folate accumulation in corn seeds, two independent *ZmGFT*‐editing mutants carrying an extra T (*Crispr1*) or A (*Crispr2*) at the 62th bp of the coding region which both resulted in a stop‐gain mutation were generated using the Crispr–CAS9 system (Figure S2A, Supporting Information). The transcript expression levels were decreased in the mutant plants (Figure S2B, Supporting Information). The *Crispr1* mutant and *Crispr2* mutant have the same truncated amino acid sequence of *ZmGFT* and identical phenotypes, therefore, we used *Crispr1* as the mutant material for subsequent experiments. Phenotypic characterization of the CAS9‐free mutant plants revealed some small differences in morphology and seed quality, as compared with the wild type, including a slightly shorter plant height and ear height, and a higher seed protein content (Figure S2C–I, Supporting Information).

**Figure 2:**
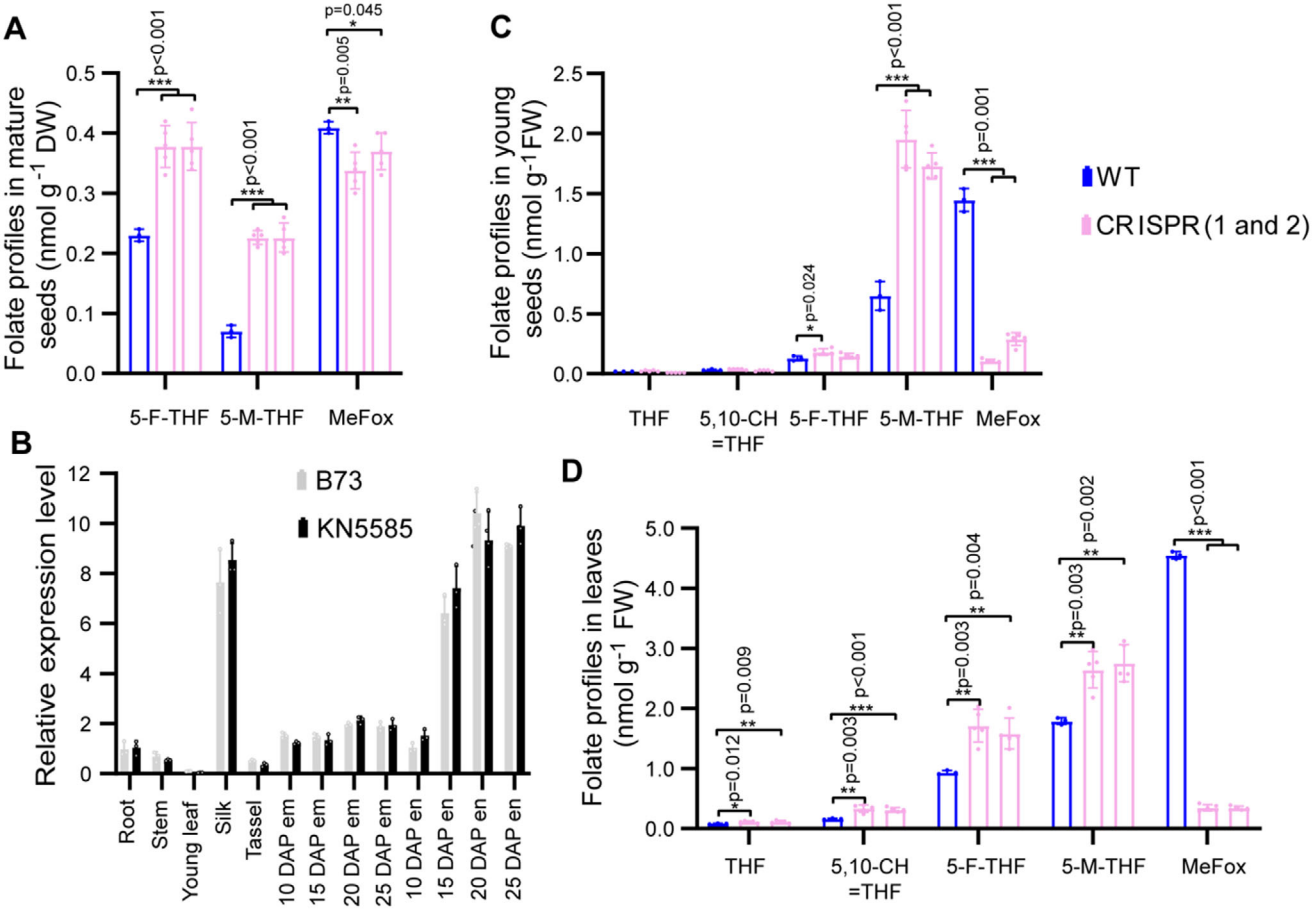
Effects of *GFT* editing and knockdown on folate accumulation in plants. A) Folate profiles in mature seeds from the wild type (WT) and *ZmGFT*‐editing (Crispr1 and Crispr2) maize plants. B) Relative expression levels of *ZmGFT* in two maize inbred lines. Gray bars, *B73*. Black bars, KN5585. DAP, days after pollination. em, embryo. en, endosperm. C) Folate profiles in young seeds from the WT and Crispr (1 and 2) maize plants. D) Folate profiles in leaves from the WT and Crispr (1 and 2) maize plants. Accumulated folate derivatives are presented as mean ± standard deviation (SD) of three for WT and five for Crispr biological replicates, respectively. *p*‐values are shown (Student's *t*‐test, *, *p* < 0.05; **, *p* < 0.01; ***, *p* < 0.001). Error bars represent standard deviations. Source data for (A–D) are provided in the Source Data file.

More importantly, loss of *ZmGFT* function led to, in mature seeds, a 3.3‐fold increase of 5‐M‐THF, a 1.7‐fold increase of 5‐formyl‐tetrahydrofolate (5‐F‐THF), and a 17% decrease of MeFox (**Figure** **2**A). MeFox, a pyrazino‐*s*‐triazine derivative of 4α‐hydroxy‐5‐methyl‐THF, is an isobaric compound of 5‐F‐THF (Figure 1B). The chromatographic detection method for MeFox was developed in maize.^[^ ^30^ ^]^ As *ZmGFT* was highly expressed in the endosperm of the seeds at 15–25 days after pollination (DAP; Figure 2B), different folate forms in the young seeds from mutant plants were further profiled, and a more pronounced MeFox reduction as much as up to 92% was observed. Similar as that in the mature seeds, a threefold increase of 5‐M‐THF and a 1.4‐fold increase of 5‐F‐THF were observed in the young seeds, with no significant changes observed for THF or 5,10‐CH═THF (Figure 2C). Such a folate profiling pattern was also observed in the leaves for 5‐M‐THF, 5‐F‐THF, and MeFox, with THF and 5,10‐CH═THF excluded (Figure 2D). Thus, *ZmGFT* was considered responsible for folate derivative accumulation in maize.

### 2.3 The Natural Variation of *GFT* Contributes to the Conversion of 5‐M‐THF to MeFox

To confirm the four highly linked polymorphic sites, i.e., Chr5.S_19676906, Chr5.S_19676907, Chr5.S_19676887, and Chr5.S_19676929, which were identified in the GWAS analyses, we resequenced the *ZmGFT* locus in 113 inbred lines from the 368‐association panel and 155‐association panel and conducted folate profiling as well as candidate gene association analysis. The data revealed these four polymorphic sites were significantly associated with levels of 5‐M‐THF (*p* ≤ 0.01, MLM; Figure 1D and Figure S3A, (Supporting Information)) and MeFox (*p* ≤ 4.84 × 10^−7^, MLM; Figure 1D and Figure S3B (Supporting Information)). The SNPs at positions S1789 and S1790 jointly cause the amino acid to change from G (Glycine) to N (Asparagine), while SNPs at positions S1770 and S1882 do not affect the amino acid sequence. Subsequently, we only focused on the S1789 (Chr5.S_19676906). Considering the differences in sample size (86 for A‐allele vs 26 for G‐allele) and the high variability of folate content in different genotypes, we analyzed the data using the median and interquartile range (IQR), in combination with the nonparametric Mann–Whitney *U* test. The results showed that the G genotype had a higher MeFox level compared to the A genotype (*p* < 0.05, Mann–Whitney *U* test < 0.05) (Tables S7 and S8, Supporting Information). Moreover, the overexpression studies also supported the superiority of the G‐allele over the A‐allele in 5‐M‐THF and MeFox accumulation (see below).

To compare the effects of the A‐ or G‐allele on folate metabolism in plants, we introduced *ZmGFT*–*B73* (carrying A‐allele) and *ZmGFT*–*Qi319* (carrying G‐allele), under the control of the constitutive ubiquitin promoter into the maize inbred line KN5585. The transcript expression levels in the overexpression lines were significantly increased (Figure S4A,B, Supporting Information). Like the *ZmGFT*‐editing plants, the overexpressors were more or less the same as the wild type with respect to plant height, and ear height, but had a higher seed protein content (Figure S4C–I, Supporting Information). Folate derivatives profiling in these transgenic plants revealed, as expected, overexpression of *ZmGFT*–*Qi319* resulted in a larger increase of MeFox than that of *ZmGFT*–*B73* in the seeds (0.65 ± 0.05 nmol g^−1^ DW vs 0.45 ± 0.02 nmol g^−1^ DW for mature seeds; 2.50 ± 0.08 nmol g^−1^ FW vs 1.88 ± 0.02 nmol g^−1^ FW for young seeds; Figure S5A,B, Supporting Information). Surprisingly, overexpression of *ZmGFT*–*Qi319*, other than *ZmGFT*–*B73*, achieved a 23% increase of 5‐M‐THF in young seeds (Figure S5B, Supporting Information). These observations demonstrated that overexpression of the G‐allele had a greater impact than the A‐allele on MeFox accumulation in corn seeds.

Amino acids were also profiled to see if gain or loss of *GFT* function in maize affects the methionine pool given that the only known metabolic fate of 5‐M‐THF is to donate one‐carbon unit for the synthesis of methionine so far.^[^ ^31^ ^]^ No significant changes of methionine in the seeds were observed in either genotype, i.e., overexpressed or mutated *ZmGFT*; however, some of the other amino acids were changed, with an increase of Glu in all changed genotypes, and a decrease of Cys in both overexpressors in mature seeds (Table S9, Supporting Information). In fact, this was not surprising since overexpression of the *ZmGFT* did not lead to a decrease of 5‐M‐THF although *GFT* is proposed to consume 5‐M‐THF, indicating an existence of a compensating mechanism for maintaining the 5‐M‐THF pool in seeds given the fact that 5‐M‐THF plays a vital role in one‐carbon metabolism.

### 2.4 Plant *GFTs* Trigger 5‐M‐THF‐to‐MeFox Conversion In Vitro

**Figure 3:**
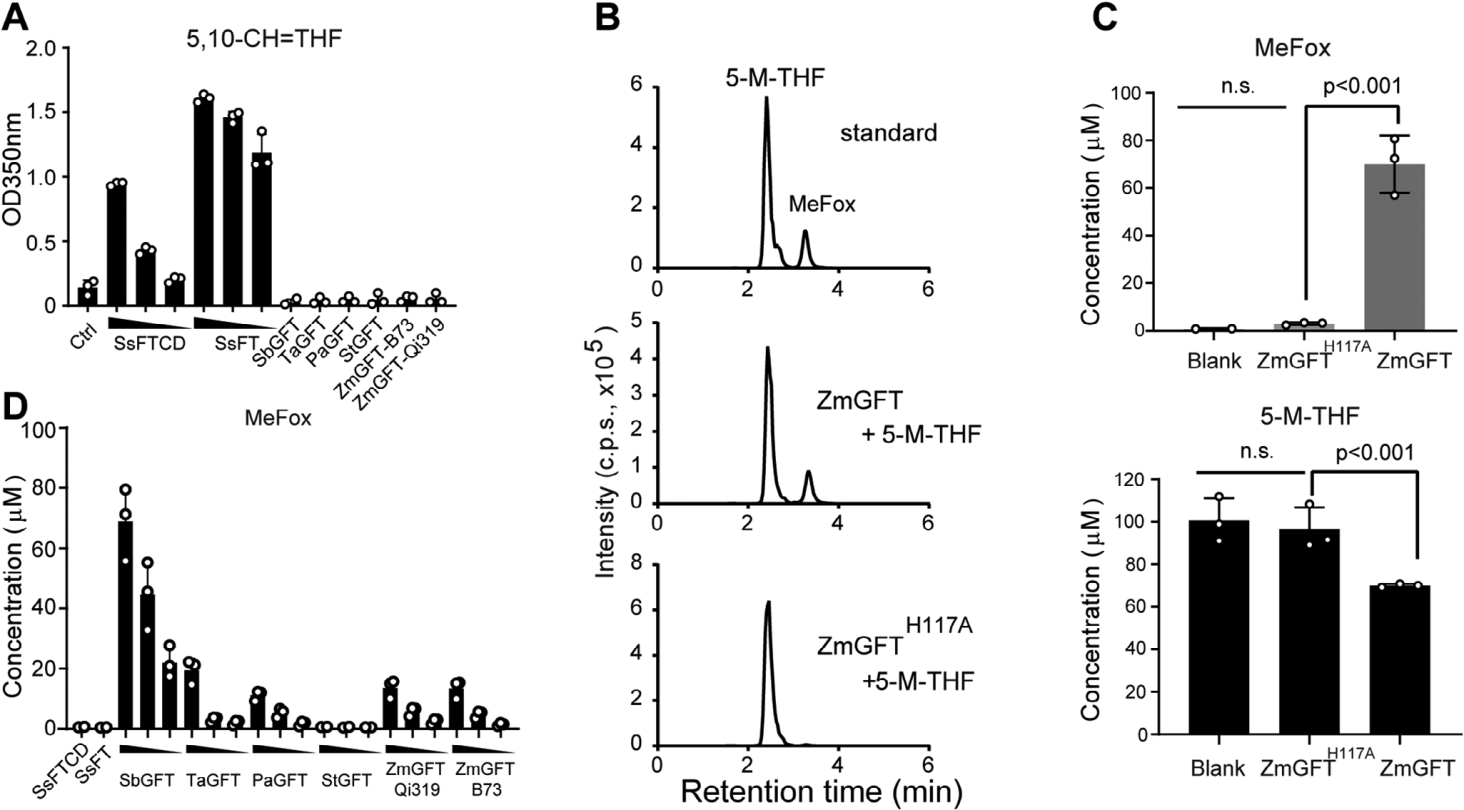
Plant *GFTs* have an activity of converting 5‐M‐THF to MeFox in vitro. A) Assays of the *formiminotransferase* activity using *SsFTCD* and *SsFT*. Note that plant *GFTs* were also included to test if they have such an activity. *SsFTCD* and *SsFT* were analyzed for their *formiminotransferase* activity at three concentrations, i.e., 10, 5, and 2 µm, and the plant *GFTs* were analyzed at 10 µm. Abbreviations: *GFT*, conversion from 5‐M‐THF to MeFox; FT, *formiminotransferase*; *FTCD*, *formiminotransferase cyclodeaminase*. Species: Pa, *Prunus avium*; Sb, *Sorghum bicolor*; Ss, *Sus scrofa*; Ta, *T. aestivum*; St, S. *tuberosum*; Zm, *Z. mays*. B) Liquid chromatography–mass spectrometry (LC–MS) detection of MeFox production catalyzed by *ZmGFT* in the presence of 5‐M‐THF. The standards of 5‐M‐THF and MeFox were detected using LC–MS (upper panel). Significant production of MeFox was detected in the presence of *ZmGFT* protein (0.4 µmol) and 5‐M‐THF (1 µmol; middle panel). When *ZmGFT* was mutated to *ZmGFT^H117A^*, MeFox production greatly diminished (lower panel). 5‐M‐THF, 5‐methyl‐tetrahydrofolate; MeFox, pyrazino‐*s*‐triazine derivative of 4a‐hydroxy‐5‐M‐THF. C) Quantification, calculated concentration from (B), of MeFox production or 5‐M‐THF consumption by the addition of *ZmGFT* or *ZmGFT^H117A^*. Each column represents a mean value of three independent measurements and the error bar represents standard deviation. *p*‐value is based on a two‐sided Student's *t*‐test. n.s., not significant. D) MeFox production (calculated concentration) catalyzed by plant *GFTs*, *SsFTCD*, and *SsFT*. The concentrations of *SsFTCD* and *SsFT* for *GFT* assay were fixed at 0.8 µm, while concentrations of plant *GFTs* for assays were 0.8, 0.4, or 0.2 µm. The data are presented as mean ± SD of three biological replicates, respectively. Source data for (A–D) are provided in the Source Data file.

To confirm the plant *GFTs* trigger 5‐M‐THF‐to‐MeFox conversion in vitro, the maize *GFTs* were expressed in Sf‐9 insect cells and other plant *GFT* proteins were expressed in *Escherichia coli* cells, followed by purification using the affinity chromatography and size‐exclusion chromatography (SEC). The SEC results exhibited that *Sorghum bicolor GFT* (*SbGFT*) and *ZmGFT* proteins are highly homogeneous and exist mainly as monomers in vitro (Figure S6, Supporting Information). The monomeric *GFT* proteins were subjected to biochemical characterization to understand how they function in folate metabolism. We first investigated whether *ZmGFT* had a *GFT* activity. As expected, both recombinant porcine (*Sus scrofa*) *formiminotransferase* (*SsFT*) and *porcine formiminotransferase cyclodeaminase* (*SsFTCD*), converted THF to 5,10‐CH═THF in the presence of *N*‐formimino‐l‐glutamate, which was consistent with the previous report.^[^ ^32^, ^33^ ^]^ However, *ZmGFTs*, i.e., *ZmGFT*–*Qi319* and *ZmGFT*–*B73*, did not have such an activity, nor the orthologs from sorghum (*SbGFT*), wheat (*TaGFT*), sweet cherry (*PaGFT*), and potato (*StGFT*) (**Figure** **3**A). Subsequently, we speculated that *ZmGFT* may participate in 5‐M‐THF‐to‐MeFox conversion given the concurrent increase of 5‐M‐THF and decrease of MeFox observed in the *ZmGFT*‐editing plants (Figure 2A,C,D). In the crystal structure of *SsFT*/ rectus (R) 5‐F‐THF, the side chain of His82 lies in proximity toward the N5 of tetrahydrofolate, and was proposed to be a base catalyst of *formiminotransferase* reaction.^[^ ^33^ ^]^ To this end, the *ZmGFT*–*B73* protein and its mutant protein bearing an alanine substitution of the conserved histidine at the position 117 (i.e., *ZmGFT^H117A^*), which was homologous to His82 of *SsFT*, were expressed and subjected to an in vitro assay using HPLC/MS. Incubation of 5‐M‐THF with the recombinant *ZmGFT*–*B73* protein resulted in production of MeFox, with ≈30% of the 5‐M‐THF being consumed in 2 h, whereas MeFox was undetectable when the protein was not supplied (Figure 3B,C). Additionally, incubation of 5‐M‐THF with monomeric *ZmGFT* or *SbGFT* resulted in a rapid increase of MeFox over 9 min (Figure S7, Supporting Information). Incubation of 5‐M‐THF with the mutant protein *ZmGFT^H117A^* produced only trace amounts of MeFox (Figure 3B,C), indicating that this conserved histidine is necessary for the activity. Other *GFTs* from plants, such as *SbGFT* or *PaGFT*, but neither *SsFTCD* nor *SsFT* from mammals, were also capable of converting 5‐M‐THF to MeFox in a dose‐dependent manner (Figure 3D). Therefore, we concluded that *ZmGFT*, a previously uncharacterized maize protein, and its plant orthologs can convert 5‐M‐THF to MeFox in vitro.

Because overexpression of the G‐allele‐carrying *ZmGFT* resulted in a folate‐accumulating pattern differing from that of the A‐allele‐carrying *ZmGFT* (Figure S5, Supporting Information), it is tempting to investigate whether this variation affects *ZmGFT*’s activity of converting 5‐M‐THF to MeFox. Specifically, we modified the favorable G‐allele in *ZmGFT*–*Qi319* to the unfavorable A‐allele, resulting in the *ZmGFT*–*Qi319^G232N^* mutant protein. Likewise, we also modified the unfavorable A‐allele in *ZmGFT*–*B73* to the favorable G‐allele, resulting in the *ZmGFT*–*B73^N228G^* mutant protein. As expected, *ZmGFT*–*B73^N228G^* displayed an enhanced activity for 5‐M‐THF‐to‐MeFox conversion (Figure S8A,B, Supporting Information), whereas *ZmGFT*–*Qi319^G232N^* had a reduced activity under the same conditions (Figure S8C,D, Supporting Information). A protein kinetic assay showed that the *k*_cat_/*K*_m_ value for *ZmGFT*–*Qi319* was 1.24 × 10^4^ ± 3.42 × 10^3^ mol^−1^ min^−1^ L, whereas it was reduced approximately by sixfold for the mutant *ZmGFT*–*Qi319^G232N^*. Furthermore, the *k*_cat_/*K*_m_ value for *ZmGFT*–*B73* was 1.51 × 10^3^ ± 1.23 × 10^3^ mol^−1^ min^−1^ L and the Asn‐to‐Gly mutation enhanced its activity, leading to a fourfold enhancement of *k*_cat_/*K*_m_ value (6.05 × 10^3^ ± 3.50 × 10^3^ mol^−1^ min^−1^ L; Figure S8E and Table S10, Supporting Information). These observations demonstrated that the natural variation at S1789 had an impact on *ZmGFT* function, with the G‐allele favoring a greater activity than the A‐allele.

Next, 5‐M‐THF was experimentally tested for if other compounds produced by *GFT* proteins using ultrahigh‐performance liquid chromatography–quadrupole time‐of‐flight mass spectrometry (UHPLC–QTOF‐MS/MS). As expected, when 5‐M‐THF was incubated with antioxidant in the absence of *GFT* protein for 2 h at 30 °C, a single peak corresponding to 5‐M‐THF appeared, but no MeFox or other folate derivatives were detected (Figure S9A, Supporting Information). However, addition of *SbGFT* or *PaGFT* to the assay buffer gave rise to a peak of the previously reported compound MeFox,^[^ ^34^ ^]^ containing the isobaric forms eluted from 4.217 to 4.287 min (Figure S9B,C, Supporting Information), demonstrating that MeFox was produced from this reaction (Figure S9D, Supporting Information). In addition, another peak at 3.34 min, whose fragments included 448.19, 406.17, 301.14, 284.11, and 259.11, was also observed (Figure S9E,F, Supporting Information), with the area being 4.29 × 10^4^ for *SbGFT* and 3.89 × 10^4^ for *PaGFT*, respectively (Figure S9G,H, Supporting Information). Unfortunately, none of the fragments was matched with any reference molecules, indicating the complexity of *GFT* reaction. To verify that the generation of MeFox is due to the action of *GFT* rather than spontaneous oxidation, 5‐M‐THF was incubated alone in the reaction buffer for 2 h. Compared the results of 5‐M‐THF incubated with *SbGFT* and β‐mercaptoethanol (Figure S10A, Supporting Information), MeFox was barely produced when 5‐M‐THF was incubated alone in the reaction buffer in the absence of antioxidants (Figure S10B, Supporting Information).

Besides 5‐M‐THF, some other folate derivatives (5‐F‐THF, Figure S9I,J, Supporting Information; THF and DHF, Table S11, Supporting Information) were also incubated with *SbGFT* and detected by UHPLC–QTOF‐MS/MS, but no signal for MeFox was retrieved. Triglutamyl 5‐methyl‐tetrahydrofolate (5‐M‐THFGlu_3_) was also tested in the in vitro assays given some folate‐metabolism‐relating enzymes prefer polyglutamylated‐THF as a substrate, such as *serine hydroxymethyltransferase* (*SHMT*)^[^ ^35^ ^]^ and *formiminotransferase cyclodeaminase* (*FTCD*).^[^ ^36^ ^]^ Consequently, no MeFox, MeFox‐Glu_3_ or other folate derivatives were detected (Table S11, Supporting Information). These observations demonstrated that 5‐M‐THF may serve as a substrate preferred by the *GFT* protein.

### 2.5 5‐M‐THF Could Be a Substrate of *GFT* Evidenced In Silico Analysis

**Figure 4:**
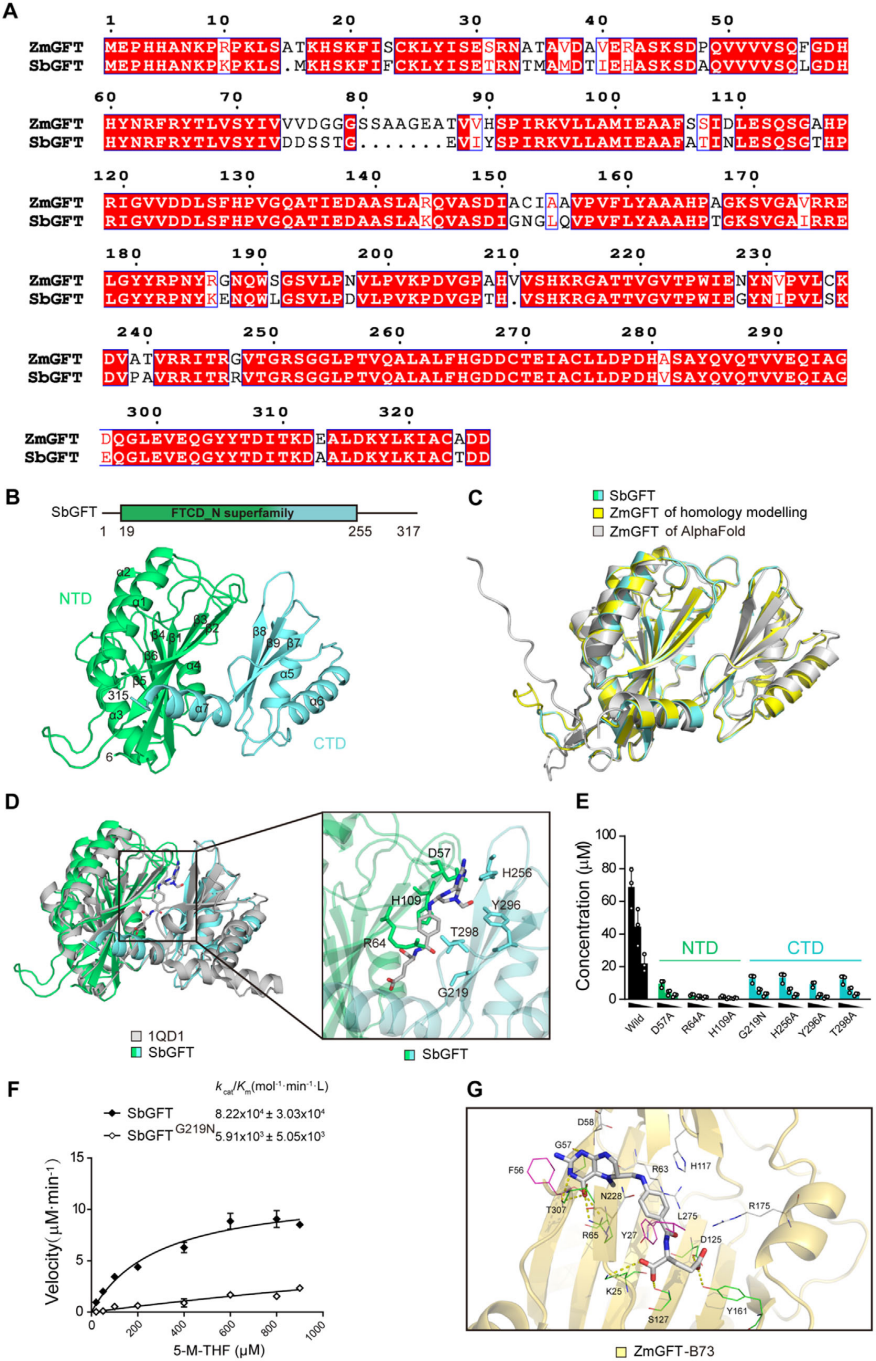
Analysis of the substrate‐binding pocket in *SbGFT* and apo *ZmGFT*. A) Alignment of amino acid sequences between *ZmGFT* and *SbGFT*. B) The overall structure of the apo state *SbGFT*. The N‐terminal domain (NTD) and C‐terminal domain (CTD) are shown in lime green and aquamarine. C) Superimposed structure of *SbGFT* (PDB code 7DYH; lime green for the NTD and aquamarine for the CTD), homology model of *ZmGFT*–*B73* (yellow), and AlphaFold model of *ZmGFT*–*B73* (gray). D) The structure of *SbGFT* superimposed with the porcine *FT* domain (PDB: 1QD1). The N‐terminal and C‐terminal domains are shown, respectively, in lime green and aquamarine, and *SsFT* is shown in gray. The ligand ([6R]‐5‐F‐THF) in *SsFT* is shown as a gray stick. All atoms are colored according to the element (carbon, gray; oxygen, red; nitrogen, blue). Zoom‐in view of the proposed substrate‐binding pocket in *SbGFT* is to the right of structure. A cartoon of *SsFT* is included for clarity. Amino acids that may interact with the substrate are indicated by sticks. E) Converting analysis of *SbGFT* proteins. The columns from left to right represent the protein concentrations used, i.e., 0.8, 0.4, and 0.2 µm, respectively. The conditions of wild‐type *SbGFT* and mutants bearing single amino‐acid substitutions used for in vitro assay are identical to these shown in NTD; CTD. The data are presented as mean ± SD of three biological replicates. F) Converting analysis of wild‐type *SbGFT* and its mutant *SbGFT^G219N^*. The velocity of the product MeFox generation is plotted against the concentration of substrate 5‐M‐THF, which ranges from 50 to 900 µm. The concentrations of *SbGFTs* are fixed at 0.5 µm, respectively. Black and white diamonds represent the values of wild‐type *SbGFT* and its mutant *SbGFT^G219N^*, respectively. *k*_cat_/*K*_m_ values of *SbGFTs* and its mutants were generated by calculating *k*_cat_ and *K*_m_ resulted from the below fitting. The data are presented as mean ± SD of two biological replicates, respectively. G) 3D binding model of 5M‐THF with *ZmGFT*–*B73*. Residues in the binding pocket are shown as lines and 5‐M‐THF is shown as a gray stick. Hydrogen bond*s* are shown as dashed lines. Source data for (D, E) are provided in the Source Data file.

We sought to resolve the crystal structure of *ZmGFT* protein in complex with 5‐M‐THF to uncover the molecular mechanism underlying how this protein triggers the 5‐M‐THF‐to‐MeFox conversion. Unfortunately, we could not generate such a complex crystal; instead, we constructed a 3D model of apo *ZmGFT* using the *SbGFT* crystal (Protein Data Bank [PDB]: 7DYH; 1.75 Å resolution) generated in this study (**Figure** **4**A,B, and Table S12 (Supporting Information)). *SbGFT* consisted of a N‐terminal domain (NTD) and a C‐terminal domain (CTD), in between which a cleft formed. Homology modeling revealed that *ZmGFT*–*B73* and *SbGFT* shared a very similar conformation with a root‐mean‐square deviation (RMSD) being over 1665 Cα atoms of 0.162 Å, and the AlphaFold^[^ ^37^ ^]^ also predicted a similar structure with the RMSD value being 0.799, indicating a high level of similarity between these two models (Figure 4C). Distance‐matrix ALIgnment analysis shows that the *GFT* protein is structurally similar to the *FTCD* from *S. scrofa* (PDB: 1QD1). This *SsFTCD* protein processively catalyzes formiminotransfer reactions through a histidine‐mediated nucleophilic mechanism. Structural and sequence comparison of the *formiminotransferase* domain with *SsFTCD* reveals conservation of the catalytic histidine H109 (Figure 4D). Additionally, other residues potentially important for binding substrate 5‐M‐THF were identified. For instance, the sidechains of D57, H256, and Y296 may form hydrogen bonds with the tetrahydropteridin ring system of the substrate, while the sidechains of R64 and T298 might interact with *p*‐aminobenzoate and glutamate moieties, respectively. Introduction of mutations into the substrate‐binding pocket in *SbGFT* resulted in a mutant proteins with significantly reduced activity (Figure 4E). It is noteworthy that the mutant protein *SbGFT^G219N^* displayed a dramatically reduced activity, indicating an important role of this amino acid for substrate binding (Figure 4F). Then, a binding pocket in the 3D model of the apo *ZmGFT* was constructed, and some amino acids, e.g., D58, R63, H117, R175, and N228, were identified to determine the shape of the binding pocket to adjust the ligand position (Figure 4G). Similar as the observation for *ZmGFT^H117A^* (see below), the mutant protein *SbGFT^H109A^*, where H109 corresponds to H117 in *ZmGFT*, also showed a significantly decreased activity (Figure 4E), indicating an accuracy of the docking‐based prediction of the binding pocket in *ZmGFT*.

Virtual screening was employed to verify the binding between 5‐M‐THF and *ZmGFT*. Most of the derivatives assessed were situated in the binding pocket of *ZmGFT*–*B73*. In the case of 5‐M‐THF (Figure S11A, Supporting Information), the key binding site N228, which corresponds to the A‐allele at S1789 which was mentioned above in GWAS results, was located near the benzene ring of the aminobenzoate. G57, R65, and T307 formed six hydrogen bonds with the aminobenzoate head of 5‐M‐THF, while K25, D125, S127, and Y161 formed another four hydrogen bonds with the glutamate tail. F56, G57, Y27, and L275 had pi–pi, amide–pi, or alkyl–pi interactions with the aromatic ring of 5‐M‐THF, and some adjacent residues such as F56 or G57 could also form hydrophobic interactions with the methyl group of 5‐M‐THF. The conformation of 5‐M‐THF binding with *ZmGFT* was like that of THF or 5‐F‐THF with *ZmGFT*, and the residues involved in the binding were almost the same (Figure S11A–C, Supporting Information). Notably, neither THF, the substrate of the mammalian *FT* domain (5‐formimino‐THF), nor the other folate derivatives had a higher docking score than 5‐M‐THF, indicating that 5‐M‐THF could be a favorable substrate for *ZmGFT* (Table S13, Supporting Information). Further molecular dynamics simulation of *ZmGFT*–*B73* in the complex with 5‐M‐THF, 5‐F‐THF, or THF, respectively, supported this conclusion. The trajectory data showed that all the three curves reached a stable convergence after 5 ns (Figure S11D, Supporting Information). The total binding free energies were −16.09 kcal mol^−1^ for 5‐M‐THF, −13.72 kcal mol^−1^ for 5‐F‐THF, and −12.99 kcal mol^−1^ for THF, respectively, indicating that 5‐M‐THF has higher binding affinity with *ZmB73* than the other two folate species do (Figure S11E, Supporting Information). These results indicated that 5‐M‐THF might be a substrate of *GFT* evidenced in silico analysis. In addition, the molecular dynamics simulations of 5‐M‐THF with *ZmGFT*–*Qi319* complex, revealing a binding free energy of −22.56 kcal mol^−1^, which is lower than the 5‐M‐THF with *ZmGFT*–*B73* complex (Figure S11E, Supporting Information). This indicates that *ZmGFT*–*Qi319* may have a stronger binding capability with 5‐M‐THF, suggestive of a higher catalytic efficiency for *ZmGFT*–*Qi319* than *ZmGFT*–*B73*.

### 2.6 *GFT* Function Is Conserved in Plants

A previous phylogenetic analysis demonstrated conservativeness of *GFT* throughout algal and land plant lineages.^[^ ^29^ ^]^ To experimentally verify if *GFT* is functionally conserved in cereal crops, expression of the *GFT* gene in rice (Os03g38540) was knocked down by RNA interference (RNAi; Figure S12A–C, Supporting Information). The *OsGFT*‐RNAi plants did not significantly differ from the wild type, with respect of effective panicle number and protein content (Figure S12D,E, Supporting Information), with a subtle decrease in amylose content and plant height observed (Figure S12F,G, Supporting Information). However, a 3.4‐fold increase of 5‐M‐THF and an 88% decrease of MeFox in the mature seeds was observed (0.83 ± 0.12 nmol g^−1^ DW vs 0.25 ± 0.03 nmol g^−1^ DW for 5‐M‐THF; 1.13 ± 0.12 nmol g^−1^ DW vs 9.02 ± 0.47 nmol g^−1^ DW for MeFox; Figure S12H, Supporting Information), similar with that observed in the loss‐of‐function maize (Figure 2A). These observations demonstrated that the endogenous *GFT* gene in rice may have the same function as that in maize.

To investigate whether the maize *GFT* functions in other plants, we introduced the *ZmGFT*–*B73* gene into *Arabidopsis* (*Arabidopsis thaliana*) under the control of the *CaMV 35S* promoter. There were no significant morphologic changes observed in the transgenic plants (Figure S13A,B, Supporting Information). Consequently, the overexpression resulted in a significant reduction of 5‐M‐THF (0.11 ± 0.01 vs 0.18 ± 0.01 nmol g^−1^ FW) and a dramatic accumulation of MeFox in the transgenic rosette leaves, whereas MeFox was undetectable in the wild‐type leaves (Figure S13C, Supporting Information). In addition to 5‐M‐THF, other folate forms, including 5‐F‐THF, 5,10‐CH═THF, and THF, were also significantly decreased in the leaves (Figure S13C, Supporting Information). These observations indicated that a heterogenous *GFT* from maize functioned to influence the folate metabolism in *Arabidopsis* as it does in maize.

Interestingly, the folate‐metabolism‐associated function of maize *GFT* was completely different from that of the *GFT* orthologs in mammals and bacteria, which catalyzes the transfer of a formimino group from formiminoglutamate to THF.^[^ ^38^ ^]^ To further understand the functional conservativeness of the *GFTs* across plant kingdom, sequences of the orthologs from plants (*ZmGFT*; *Sorghum bicolor GFT*, *SbGFT*; *TaGFT*; *OsGFT*; *StGFT*; *Prunus avium GFT*, *PaGFT*; *Lunaria annua GFT*, *LaGFT*; *Arabidopsis GFT*, *AtGFT*; and *Selaginella moellendorffii GFT*, *SmGFT*), cyanobacteria (*Crucigenia variabilis GFT*, *CvGFT*), and microalgae (*Volvox carteri GFT*, *VcGFT*) were aligned to that of the bacterial *glutamate formiminotransferase* (*Gloeobacter kilaueensis GFT*, *GkGFT*; *E. coli GFT*, *EcGFT*) and mammalian *formiminotransferase cyclodeaminase* (*S. scrofa FTCD*, *SsFTCD*; *Homo sapiens FTCD*, *HsFTCD*; *Mus musculus FTCD*, *MmFTCD*; *Rattus norvegicus FTCD*, *RnFTCD*) to construct a phylogenetic tree (Figure S14, Supporting Information). It is clearly shown that all the orthologs aligned were grouped into two classes, with class I consisting of the orthologs from cyanobacteria (*CvGFT*) and microalgae (*VcGFT*) as well as plants, and class II from bacteria and mammals. Among the plant orthologs, *ZmGFT* had the highest level of identity (81.6%) to its sorghum ortholog (*SbGFT*, 317 aa) and, interestingly, the cruciferous *GFT* (such as *AtGFT* and *LaGFT*) had a distinct grouping pattern, genetically distant from the other plant *GFTs* (Figure S14A, Supporting Information). Overall, the motif *GFT*/*FTCD‐N* was found well conserved across all the 17 different species, and the motif *FTCD‐C* was only conserved among the mammals (Figure S14B, Supporting Information). Albeit the *GFT*/*FTCD‐N* motif was conserved across all species, *GFT* differed in biological function as evidenced by an activity of converting 5‐M‐THF to MeFox in plants (Figure 3D) and that of transferring formimino group to THF in mammals,^[^ ^38^ ^]^ respectively. The analyses suggested a conserved function mediated by the *GFTs* in folate metabolism in plants.

### 2.7 Potential of the Natural Variation Identified in Folate Biofortification in Maize

**Figure 5:**
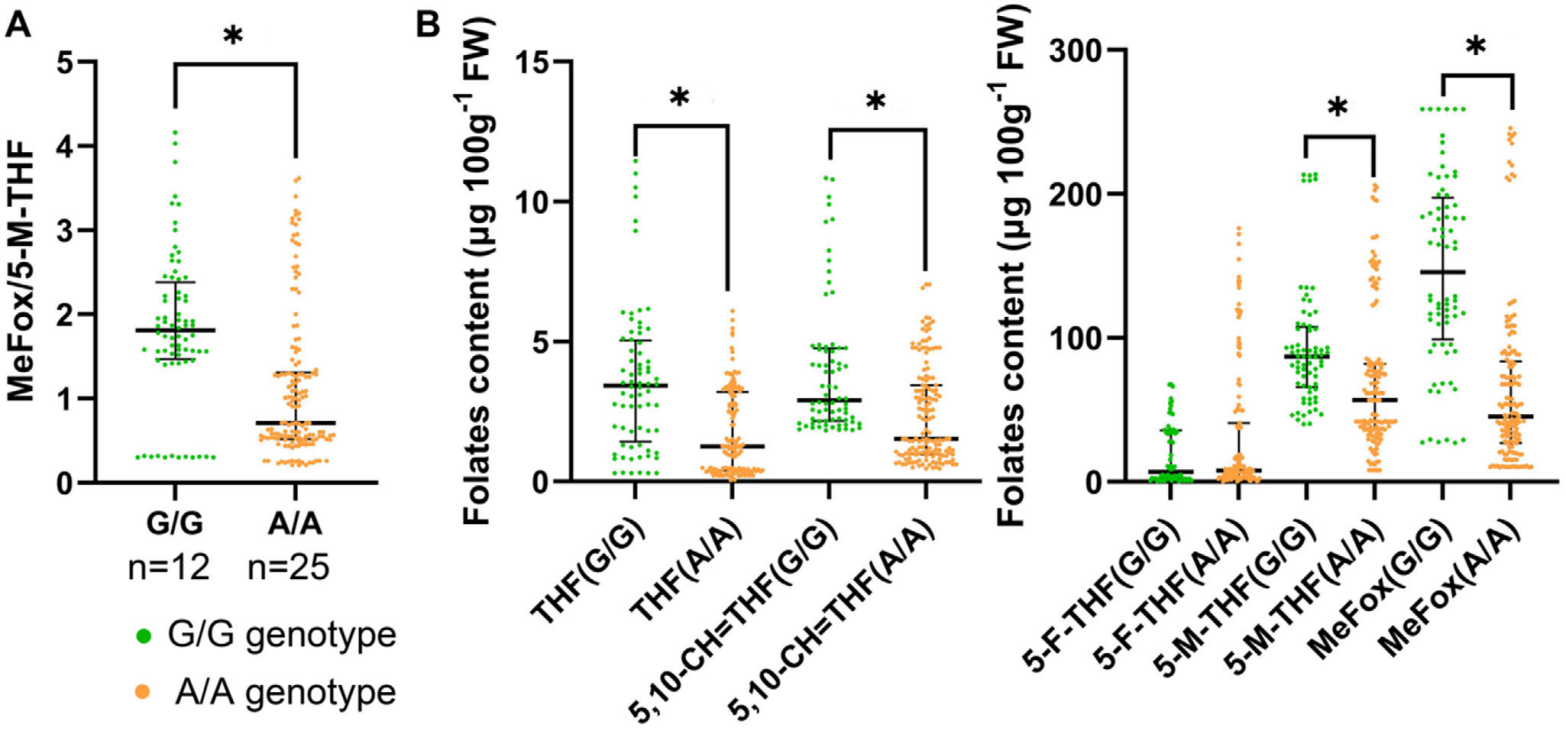
Folate profiles of commercial hybrid sweetcorn. The young seeds were collected at 20–24 DAP (harvest stage) for folate detection. A) MeFox to 5‐M‐THF ratio of sweetcorn with homozygous G/G‐allele and A/A‐allele. B) Folate profile of young seeds from sweetcorn with homozygous G/G‐allele and A/A‐allele, including MeFox, 5‐M‐THF, 5‐F‐THF, 5,10‐CH═THF, and THF. The dot plots display the distribution of MeFox/5‐M‐THF ratios (A) and folate contents (µg 100g^−1^ FW) (B) across different genotypes. Each dot represents an individual raw data point. The solid horizontal line indicates the median, and the thin horizontal lines indicate the interquartile range (IQR). Statistical significance was assessed using the two‐sided Mann–Whitney *U* test. Asterisks indicate significant differences (*, *p* < 0.01). The data are presented as median and IQR from six biological replicates, respectively. Source data for (A, B) are provided in Table S14 (Supporting Information) and the Source Data file.

Since the natural allelic G/A variation at S1789 had an important impact on 5‐M‐THF pool and folate metabolism as revealed in the study mentioned above, we assessed the potential of this variation in folate biofortification in sweetcorn due to its human preference at young stage and a close kinship with common corn.^[^ ^39^ ^]^ In total, 47 sweetcorn hybrids were collected, with 53.2% (25 of 47) being homozygous for the A‐allele (A/A), 25.5% (12 of 47) being homozygous for the G‐allele (G/G), and 21.3% (10 of 47) being heterozygous G/A (Table S14, Supporting Information). Folate profiling analyses revealed a higher MeFox/5‐M‐THF ratio, along with a higher level of 5‐M‐THF and MeFox accumulation, in the G/G genotype than those in the A/A genotype (1.81 (1.47–2.38) vs 0.71 (0.52–1.31) µg per 100 g, **Figure** **5**A, 86.99 (65.89–107.71) µg per 100 g FW vs 56.84 (36.45–81.84) µg per 100 g FW for 5‐M‐THF and 145.71 (98.98–197.78) µg per 100 g FW vs 45.42 (26.87–83.61) µg per 100 g FW for MeFox, Figure 5B). In addition, the G/G genotype exhibited higher levels of THF and 5,10‐CH═THF accumulation than the A/A genotype (Figure 5B).

Total folates in G/G (103.81 (85.68–152.31) µg per 100 g FW) and G/A lines (239.83 (175.08–263.18) µg per 100 g FW) were significantly higher than those in A/A lines (66.73 (44.41–121.19) µg per 100 g FW) (Figure S15A and Table S14, Supporting Information). When MeFox was also included as a form of folate derivative, the difference in total folate levels became more striking. The total folate content in G/G was 286.14 (188.27–343.44) µg per 100 g FW and in G/A was 332.49 (289.48–469.85) µg and 100 g FW, both higher than that in A/A (110.51 (82.60–284.72) µg per 100 g FW) (Figure S15B and Table S14, Supporting Information). These observations were consistent with the increased 5‐M‐THF and MeFox levels observed in the young seeds of the *ZmGFT*–*Qi319* overexpressors (Figure S5, Supporting Information). The median levels of total folates (excluding MeFox) in G/A sweetcorn and G/G sweetcorn were 60% and 26% of the dietary allowance recommended by the World Health Organization, respectively, if 100 g is consumed per day,^[^ ^15^ ^]^ whereas that in A‐allele sweetcorn only meets 16% (Figure S15A, Supporting Information). Additionally, G/A hybrids contained more folates than the other two genotypes (Figure S15, Supporting Information), providing an elite source for folate biofortification in corn. These results demonstrated that the G‐allele is one of the favorable alleles for folate accumulation in sweetcorn and could be used as a molecular marker for the development of high‐folate corn varieties.

## 3 Discussion

Understanding of the biochemical function of plant *GFTs* will shed a new light into the metabolic fate of 5‐M‐THF, beyond the long‐standing knowledge that methylenetetrahydrofolate reductase (MTHFR) converts polyglutamated 5,10‐methylene‐THF to 5‐M‐THF, which subsequently enters methionine cycle catalyzed by methionine synthase (METS) by serving as a one‐carbon donor.^[^ ^31^, ^40^ ^]^ Although we previously also observed a 5‐M‐THF accumulation resulting from the loss of *GFT* function in millet and rice,^[^ ^41^, ^42^ ^]^ neither enzymatic assay nor molecular docking was conducted to unravel the mechanism underlying the 5‐M‐THF‐to‐MeFox formation. Here, we performed a comprehensive investigation both in vivo and in vitro by employing various genetic and biochemical approaches and demonstrated that 5‐M‐THF and MeFox are the substrate and product of *GFT* protein, respectively, as well as the natural variations identified played an important role in 5‐M‐THF accumulation in maize. In *Setaria italica*, knockout of the *glutamate formiminotransferase* genes *SiGFT1* and *SiGFT2* led to a fourfold increase in bioactive folates compared to wild‐type plants, while simultaneously reducing the MeFox by 95%, highlighting a promising approach for folate biofortification in cereal crops.^[^ ^41^ ^]^ Disruption of *OsGFT* resulted in a 90% reduction in MeFox level and a twofold to threefold increase in bioactive folate content.^[^ ^42^ ^]^ Phylogenetic analysis reveals a close evolutionary relationship among the *GFTs* of maize, rice, and foxtail millet (Figure S14, Supporting Information). In combination with the previous observation in millet and rice, we concluded that the *GFT* function is likely to be conserved among plants, if not all. All the observations indicate that *GFT* plays an important role in 5‐M‐THF metabolism and can be an elite target gene for folate biofortification in cereal crops. Albeit the plant *GFTs* shared a high level of similarity with the *FT* domain of mammalian *FTCD*, they did not show the classical activity of *GFT* as the porcine *FT* did, indicating that *GFT*, the presumed ortholog of the *GFT* in mammals, has evolved a new biological function in plants. *N*‐formimino‐glutamate, a toxic breakdown product, is produced during histidine degradation in mammals, and catalyzed to formimino‐THF by *glutamate formiminotransferase* in the presence of THF as previously reported (Figure 1A).^[^ ^43^ ^]^ However, the mammalian‐type histidine degradation pathway has not been reported in plants yet,^[^ ^44^ ^]^ which is consistent with the observation that all the plant *GFTs* investigated did not have a *GFT* activity in our study (Figure 3A). Likewise, the *SsFT* domain or *SsFTCD* did not convert 5‐M‐THF to MeFox either (Figure 3D). Apart from catalyzing formimino transfer, mammalian and bacterial *FT* can also mediate a reversible formyl transfer between 5‐F‐THF and THF (Figure 1A).^[^ ^38^ ^]^ Taken together, we conclude that *GFT* participates in folate metabolism by triggering 5‐M‐THF‐to‐MeFox conversion in plants with an activity differing from its ortholog in mammals and bacteria. Therefore, we propose the alternative name MeFox Synthase to better reflect its biochemical role.

Elucidating how *GFTs* trigger 5‐M‐THF‐to‐MeFox conversion at molecular level is a long way to go. Previous studies showed that MeFox could be formed when 5‐M‐THF was exposed to strong oxidative conditions, such as continuous oxygen treatment or incubation with H_2_O_2_, catalase, or copper sulfate.^[^ ^45^, ^46^, ^47^ ^]^ However, MeFox remained barely detected within 2 h when 5‐M‐THF was incubated alone in the reaction solution in either presence or absence of antioxidants (Figures S9 and S10, Supporting Information). When plant *GFTs* were present in the buffer, MeFox was increased significantly (Figure 3D and Figures S9 and S10, Supporting Information), demonstrating that MeFox was formed via a *GFT*‐triggered catalysis other than an auto‐oxidation.

We examined the ratio between the decrease in MeFox and the increase in other folate vitamers, and observed that the former is not proportional to the latter in the mature seeds and leaves. For example, there was an increase of 0.30 nmol g^−1^ in folates and only a decrease of 0.056 nmol g^−1^ in MeFox in the mature seeds (Figure 2A). However, the increase in folates seemed to be proportional to the decrease in MeFox in young seeds (1.23 vs 1.25 nmol g^−1^) (Figure 2B). These observations suggest an existence of differentially regulated folate metabolisms in different tissues at different developmental stages. MeFox is an oxidative form of folate derivative and can be also generated through a *GFT*‐independent mechanism during seed maturation when oxidative stress takes place. Moreover, our unpublished transcriptomic data showed the expression level of *GFT* is gradually decreased during kernel development from the DAP 25 to DAP 35, indicating that the *GFT* may not be a major player for MeFox formation during the late developmental stage in maize seeds. These observations explained at least partially the more pronounced decrease of MeFox accumulation in young seeds than in mature seeds of the *GFT*‐knockout mutant (92% vs 17%). It is also important to point out that the presence of significant level of MeFox in the mutant seeds indicates that the *GFT* is not the only protein responsible for MeFox generation. Maize contains two *GFT* homologs with a sequence similarity of 64.5%. Currently, it remains unknown whether the other homolog, *GFT2*, possesses a biological function similar to the *GFT* investigated in this study. The *GFT*‐knockout rice plants had a significant decrease of MeFox, accompanied by a dramatic increase in 5‐M‐THF.^[^ ^42^ ^]^ In this study, we used *GFT*‐RNAi lines of rice and observed a markable reduction in MeFox. However, the increase in 5‐M‐THF was less pronounced than expected. We speculate that this discrepancy may be attributed to the residual enzymatic activity of *GFT* in the RNAi lines which still allowed 5‐M‐THF to maintain a metabolic homeostasis other than to accumulate at a high level.

People have proposed a two‐step reaction that how 5‐M‐THF is transformed into MeFox.^[^ ^34^ ^]^ The first step is that a hydroxyl group is added to the position 4α on pteridine ring of the 5‐M‐THF molecule by oxidation and transformed into 4α‐hydroxy‐5‐methyltetrahydrofolate, an intermediate product also called hmTHF. The second step is that hmTHF undergoes an intramolecular structure rearrangement to form, in the absence of reducing agent, a stable pyrazino‐*s*‐triazine derivative known as MeFox. In fact, it is not difficult for the pteridine ring to undergo such an extensive structural change given that only one chemical bond breakage and one chemical bond formation is required, respectively, during the process. Thus, we speculate that the activity of *SbGFT*/*ZmGFT* is to accelerate the conversion of 5‐M‐THF to MeFox. The oxidation could first happen on position 4α of the pteridine of the 5‐M‐THF molecule using oxygen as an electron acceptor to form a hydroxyl group, and then the intermediate undergoes a rearrangement to form MeFox (Figure S16, Supporting Information).^[^ ^34^, ^45^, ^46^, ^47^, ^48^ ^]^ Considering the structural rearrangement of hmTHF is automatic in none‐reducing environment, we speculate that monomeric *GFT* can accommodate 5‐M‐THF in its substrate binding pocket and subsequently facilitate the oxidation on 4α position of pteridine ring and subsequent structural rearrangement. Due to lack of the crystal structure of *GFT* complexed with 5‐M‐THF and explicit biochemical evidence, we are unable to determine the specific catalytic mechanism. We believe that a detailed elucidation of the molecular mechanism of the *GFT*‐mediated 5‐M‐THF‐to‐MeFox conversion will be eventually dependent on both dissection of the crystal structure of *GFT*/folate complex and identification of a series of intermediate products from the in vitro reaction.

A peak containing the isobaric forms eluted from 4.217 to 4.287 min was observed, and all the MS scans of the complete MeFox peak were investigated. They all contained equivalent compositions and thus identical masses with a slight difference in elution time from the tandem mass spectrometry analysis, indicating this wide peak may represent different isobaric forms (Figures S9B and S10A, Supporting Information). The mechanism of how isobaric forms are produced remains unknown in most cases; chemical ionization reaction mass spectrometry has shown promise for differentiating plant‐derived isobaric molecules, such as aldehydes and ketones, by selecting specific reaction conditions.^[^ ^49^ ^]^ At present, it is a pity that we are unable to structurally identify those isobaric forms due to technical difficulties. Identification of the *GFT*‐triggered production of MeFox has also led to a big concern about its biological or physiological significance in plants. For example, one may speculate that MeFox can serve as an oxidized storage form of 5‐M‐THF, and be recycled upon certain developmental signals. After all, the reason why MeFox is formed in plants remains an enigma.

Metabolic flow of 5‐M‐THF to methionine or 5,10‐CH═THF is regarded as a major route in plants,^[^ ^50^ ^]^ but we could not rule out a new metabolic fate of 5‐M‐THF, such as *GFT*‐triggered conversion, apart from a C1 donor for methionine synthesis from multiple disciplines such as genetics and molecular biology. Also, we found that the in vitro reaction condition for *GFTs* was similar like that for *glutamate formiminotransferase* or pteridine oxidase.^[^ ^32^, ^51^ ^]^ *Glutamate formiminotransferase* activity was detected in animals, and pteridine oxidase was reported only in two plant species, *Ricinus communis* L. and *Sinapis alba* L., possessing an activity of hydroxylating and oxidizing 2‐amino‐4‐hydroxypteridine.^[^ ^51^ ^]^ We believe that a detailed elucidation of the molecular mechanism of the *GFT*‐mediated 5‐M‐THF‐to‐MeFox conversion will be eventually dependent on both dissection of the crystal structure of *GFT*/folate complex and identification of a series of intermediate products from the in vitro reaction.

Acquiring structure of a protein can be of a great help for understanding its biological function. In this study, we failed to obtain the structure of the maize *GFT*; instead, we were successful for sorghum *GFT*, with a sequence identity of 81.6%, thus allowing construction of a 3D structure of maize *GFT* by homologous modeling and adoption of molecular simulation to investigate protein‐ ligand binding.^[^ ^52^, ^53^ ^]^ It is observed that although the three folate derivatives investigated exhibited a similar binding mode, some subtle changes led to different binding abilities, with 5‐M‐THF possessing the lowest binding free energy and thus serving as the most suitable substrate (Figure 4 and Figure S11 and Table S13 (Supporting Information)). However, it is necessary to point out that a more comprehensive study is needed to eventually elucidate the catalytic mechanism of *GFT* protein.

5‐M‐THF serves as the substrate for *GFT*, while a stable 5‐M‐THF accumulation was observed in the *GFT*‐overexpressing plants. The G/G genotype, with a higher activity, is expected to consume more 5‐M‐THF than the A/A genotype. However, a higher level of 5‐M‐THF was detected in G/G sweet corn (Figure 5 and Figure S15 (Supporting Information)). To better understand this phenomenon, we performed a transcriptomic analysis of young seeds (DAP 25) from wild‐type, CRISPR, OE‐B73, and OE‐Qi319 and investigated the expression of folate metabolism‐relating genes (Figure S17 and Table S15, Supporting Information). As a result, both OE‐B73 and OE‐Qi319 lines, some folate‐related genes, such as *5‐FCL*, *ADCL1*, *ADCL2*, *DHC3*, *DHNA1*, *DHFR3*, *DHFR4*, *GCH‐1*, *GCSH2*, *GCSH3*, *GCST*, *HPPK/DHPS*, *MS1*, *MTHFR1*, *SHMT7‐1*, and *SHMT7‐2*, exhibit significantly elevated expression. These genes participate in the folate biosynthesis pathway and the interconversion of folate derivatives. Notably, the genes encode MTHFR (involved in 5‐M‐THF synthesis directly), METS, *GCST*, and *SHMT*, were previously identified and had a strong positive correlation with 5‐M‐THF levels in maize kernel.^[^ ^54^ ^]^ Hence, a plausible explanation would be that *GFT* overexpression is likely to trigger a shift of the folate metabolic flux toward 5‐M‐THF to maintain its homeostasis to ensure the plant growth and development, and G/G possess a stronger “force” than A/A to drive the metabolic flux toward 5‐M‐THF. The observation of the G/A‐allele exhibiting a higher folate level than G/G‐allele was somewhat unexpected. We speculate that the heterozygous G/A‐allele might exhibit a synergistic effect via an unknown mechanism, leading to a high level of 5‐M‐THF accumulation compared to G/G. Obviously, a detailed investigation is needed.

A recent study reported the same SNP as we identified in this study, unfortunately, the gene function and the SNP effects were not precisely characterized.^[^ ^55^ ^]^ In this study, selection for favorable G‐allele in *ZmGFT* is effective for increasing folate concentrations in corn, as the G‐allele favors the accumulation of folates in both transgenic plants and conventional sweetcorn hybrids. Hybrids containing favorable G‐allele in *ZmGFT* gene yielded an average increase in total folates of 100 µg per 100 g, above the average of A‐allele‐containing hybrids (Figure 5). Regardless of the fact this Asn/Gly mutation had a significant impact on the maize *GFT* activity, it seems that neither Asn nor Gly residue is conserved among the plant species investigated. For example, the residue at the same position is serine in wheat (*TaGFT*), leucine in sweet cherry (*PaGFT*), and methionine in potato (*StGFT*), respectively (Figure S1, Supporting Information). In silico molecular docking analysis revealed that *ZmGFT*–*Qi319* had a greater binding affinity to 5‐M‐THF than *ZmGFT*–*B73* (Table S16, Supporting Information), which was attributable to that G232 occupied less space than N228 (Figure S8A,B, Supporting Information), thus favoring the conversion. These observations indicate that an amino acid variant with enhanced interaction with substrate or intermediate could contribute to improving enzyme activity, as observed in the thermostable β‐ketothiolase.^[^ ^56^ ^]^ From the practical viewpoint of improving human folate status, consumption of less G‐allele‐containing corn, in comparison with A‐allele‐containing corn, will readily achieve the dietary allowance recommended by the World Health Organization. Further, the natural A‐to‐G single nucleotide variation identified in this study may serve as a molecular marker to generate more G‐allele carrying inbreds during breeding process to boost the metabolic flow toward the accumulation of 5‐M‐THF. Enhancing the nutritional value of staple crops to produce biofortified foods is crucial and has become one of the 100 essential questions for the future of agriculture.^[^ ^57^ ^]^ This natural A‐to‐G variation contributing to the variation of folate accumulation in mature seeds of a maize inbred line population, which would be also useful for breeding folate‐fortified maize varieties, as the favorable alleles involved in carotenoid metabolism were used for developing carotenoid‐rich crops,^[^ ^27^, ^58^, ^59^, ^60^ ^]^ thus providing consumers with nutritious foods for their health benefits. In our case, a higher level of both 5‐M‐THF and MeFox was detected in G/G sweet corn than in A/A sweet corn (Figure 5B). Considering that the sweet corn hybrids were collected from different sources, we further analyzed the possible impact of geographic origins on the G/A‐allele's role in folate metabolism. In the samples sourced from Beijing, no significant differences in MeFox and 5‐M‐THF levels were observed between the G‐ and A‐allele, suggesting that the effect of the G/A‐allelic variation on 5‐M‐THF or MeFox accumulation may be location‐dependent (Figure S18, Supporting Information). Further G × E effects should be examined by collecting more additional sweet corn hybrids from a broader range of geographic origins in the future. 5‐M‐THF offers significant metabolic advantages as it does not require hepatic activation and is directly bioavailable in human body, reducing the risk of unmetabolized folic acid accumulation in the bloodstream. This is particularly beneficial to those with genetic polymorphisms (e.g., MTHFR variants) or impaired liver function, where conversion of folic acid to its active form is limited.^[^ ^61^ ^]^ Regarding MeFox, its biological function in both plants and animals remains completely unknown. High levels of accumulation of over 1000 µg per 100 g in cereal crops including rice and wheat were previously detected.^[^ ^30^ ^]^ However, it is believable that MeFox may do no harm to human health given the fact that humans have consumed rice and wheat for thousands of years. Likewise, we would like to conclude that MeFox in sweet corn is unlikely to pose any adverse effects on human health especially when its concentration is much lower in sweet corn than in other staple food crops. The average total folate (excluding MeFox) content in gene‐edited maize (Crispr lines) was 94.65 ± 11.42 µg per 100 g FW. Folate loss typically occurs during corn processing. An average folate reduction of 45% was observed in boiled corn, while steaming and microwave heating resulted in a loss of 12% or 15%, respectively.^[^ ^62^ ^]^ When steaming‐caused loss is taken into account, a daily intake of 200 g of the gene‐edited maize is expected to meet 41.6% of the recommended daily allowance and 25–26.0% of the recommended daily allowance for boiled corn. Fresh sweet corn also can be processed as juice, thus consumed directly with a high level of folate retention, highlighting the importance of breeding high‐folate fresh sweet corn varieties to enhance human folate intake.

## 4 Experimental Section

### Plant Materials and Field Trials

A diverse maize association panel consisting of 531 maize inbred lines was used for folate profiling.^[^ ^26^ ^]^ The panel was planted in three field trials including Hainan in 2009 (09ZHN), Yunnan in 2010 (10WY), and Hubei (10AMH) in 2010. Samples were harvested from 501, 406, and 464 lines in these environments, respectively. Field experiments followed the procedures described previously, where one row was planted for each inbred line and at least six ears in each row were self‐pollinated for all 531 lines.^[^ ^27^ ^]^

A collection of 113 lines from the panel containing 368 maize inbred lines, which were part of the 531‐association panel were grown in Hebei (China) in 2019. A commercial collection of 47 fresh corn hybrids were collected from Shanghai, Guangdong, Beijing, and Sichuan (all in China) and grown in in Hebei (China) in 2020. Field experiments followed the procedures described previously.^[^ ^23^ ^]^ For the 113 inbred lines, one row was planted for each inbred line and at least three ears in each row were self‐pollinated and harvested for folate profiling of mature seeds. For the 47 commercial hybrid lines, one row was planted for each line and at least six ears in each row were self‐pollinated and harvested for folate profiling of the young seeds (DAP 20 to 24). Six samples were detected in two rounds in total. In each sample, kernels from the middle part of three cobs were collected. In round 1, samples 1, 2, and 3 were extracted and detected twice, and so for samples 4, 5, and 6 in round 2, then their median values and interquartile ranges of folate levels were calculated.

### Genome‐Wide Association Study

In total, 368 inbred lines from the abovementioned 531‐association panel were genotyped using RNA‐Seq.^[^ ^27^ ^]^ 1.03 million high‐quality SNPs were identified by RNA sequencing. Furthermore, 5‐M‐THF was measured in mature seeds of 513 maize inbred lines grown in the three environments mentioned above. The BLUP approach based on a mixed linear model was used for reducing the influence from the environment.^[^ ^63^ ^]^ In total 558 529 SNPs with a MAF > 0.05 were used to investigate the association with 5‐M‐THF levels on the base of BLUP data across these three environments by means of a mixed linear model that considered population structure and individual relatedness.^[^ ^64^ ^]^ Population structure and individual relatedness were calculated using 16 338 SNPs with <20% missing data and MAF >5% with a Bayesian Markov Chain Monte Carlo program integrated in software STUCTURE.^[^ ^65^ ^]^ and using the method of Loiselle et al.,^[^ ^66^ ^]^ respectively. A strict threshold (*p* = 0.05/*n* = 6.99 × 10^−7^, where *n* = total markers used) was set to identify considerable genome‐wide SNPs.

### Resequencing of Candidate Gene *ZmGFT*

The sequence of the candidate gene *ZmGFT* was obtained from the *B73* reference at the MaizeSequence database (http://ensembl.gramene.org/Zea_mays/Info/Index?db = core). Primers were designed using Primer Premier 5 software to cover the entire gene, including 5′‐UTR (350 bp), exons, introns, and 3′‐UTR (50 bp). Polymerase chain reaction products of 113 inbred lines from 368‐association panel and 155‐association panel, were subsequently sequenced. The sequences were assembled using ContigExpress.^[^ ^67^ ^]^ and aligned using MUSCLE40,^[^ ^68^ ^]^ then refined manually in BioEdit.^[^ ^69^ ^]^ Nucleotide polymorphisms including SNPs with a frequency of ≥ 0.05 were extracted. Candidate gene association analysis based on these polymorphic markers was further conducted using the abovementioned mixed linear model.^[^ ^63^ ^]^

### Transgenic Analysis

*ZmGFT*‐editing maize plants carrying stop‐gain mutation were generated using the Crispr–Cas9 system,^[^ ^70^ ^]^ with an SgRNA target sequence of CCAAGTTCATCTCCTGCAAG. The *ZmGFT* open reading frames of the *B73* (S1789 = A) and *Qi319* (S1789 = G) inbred lines driven by the ubiquitin promoter were introduced into the maize hybrid line KN5585 via *Agrobacterium*‐mediated transformation to obtain transgenic lines.^[^ ^71^ ^]^ The endogenous *GFT* gene of KN5585 carried G at S1789. After T1 seeds were obtained, all transgenic plants were self‐pollinated for two generations. Three individual lines of homozygous *ZmGFT*‐editing, *ZmGFT*–*B73* overexpressing, and *ZmGFT*–*Qi319* overexpressing maize were characterized for 2 years, and representative data from 1 year were shown in this study because the lines exhibited similar patterns. The highest blades at day after sowing 70, young seeds (DAP25), and mature seeds of third‐generation‐transgenic plants were collected for folate profiling. The *ZmGFT* open reading frames of the *B73* (S1789 = A) inbred lines driven by the CaMV35S promoter were introduced into *Arabidopsis* Columbia via *Agrobacterium*‐mediated transformation to obtain transgenic lines.^[^ ^72^ ^]^ Rosette leaves of the transgenic plants were grown for 30 days and collected for folate profiling. Two individual lines of homozygous *ZmGFT*–*B73* overexpressors and *ZmGFT*–*Qi319* overexpressors were characterized twice, and represented data from one assay were shown in this study because they exhibited similar patterns. To knock down *OsGFT* (*Os03g38540*), an ortholog of *ZmGFT* in rice genome, an RNAi construct containing 360‐bp *OsGFT*‐specific fragment under the control of the ubiquitin promoter was also transformed into the *O. sativa* subsp. japonica rice cultivar Yandao 8 via *Agrobacterium*.^[^ ^73^ ^]^ Mature seeds of the transgenic plants were collected for folate profiling. Five individual lines of homozygous *OsGFT*‐RNAi were characterized twice, and represented data from one assay were shown in this study because they exhibited similar patterns. Plant heights and ear lengths of the mature wild type and transgenic maize and rice were measured by a ruler. Hundred‐grain and ear weights were measured using a weighing scale, and protein content and starch content were measured by near‐infrared‐transmission spectroscopy (InfratecTM1241 Grain Analyzer; Foss, Denmark) when the mature seeds were harvested.

### Transcriptome Analysis

Total RNA was extracted from maize kernels of wild type, knockout, and overexpression lines at DAP 25 using the BAIXU Maize Kernel Total RNA Extraction Kit (Beijing, China). To remove residual genomic DNA, the RNA samples were treated with RNase‐free DNase I (New England Biolabs, Ipswich, MA, USA). First‐strand complementary DNA (cDNA) was synthesized from the purified RNA using the RevertAid First Strand cDNA Synthesis Kit (Fermentas, Waltham, MA, USA). RNA‐Seq library preparation, sequencing, and data analysis were carried out by TsingkeBiotechnology Co., Ltd. (Beijing, China).

### Real Time‐Polymerase Chain Reaction

The *B73* and KN5585 inbred lines were used to investigate potential patterns. Roots, stems, young leaves, tassels, and embryos/endosperm at 10, 15, 20, or 25 DAP were collected separately. Transcript levels of *ZmGFT* were also analyzed in the transgenic plants. Total RNA was extracted with TRIzol reagent.^[^ ^74^ ^]^ cDNAs were constructed using the first‐strand CdnA synthesis kit (USA, https://www.thermofisher.cn/). Quantitative real‐time polymerase chain reactions were performed with an ABI7500 (USA, https://www.thermofisher.cn/). The primers used in this experiment are listed in Table S17 (Supporting Information).

### Extraction and Profiling of Folates

Preparation of mature seeds from the inbred line samples (09ZHN, 10WY, and 10AMH) for testing of folate composition, including 5‐M‐THF, was performed as described by Jiang et al.^[^ ^28^ ^]^ Detection of folate derivatives, including MeFox in fresh leaves, young seeds, and mature seeds from maize, rice, and *Arabidopsis* was carried out according to the method described by Shahid et al.^[^ ^30^ ^]^ Folate separation and quantification was performed using an Agilent 1260 HPLC system coupled with an Agilent 6420 triple‐quadrupole tandem MS operated in positive electrospray ionization (ESI) mode (Palo Alto, CA, USA). A Kromasil 100−5 C18 column (50 × 2.1 mm, 2.5 µm particle size) with an Agilent SB‐C18 precolumn (2.1 × 5 mm, 2.7 µm particle size) was used. The mobile phases were 0.1% v/v formic acid in water (phase A) and 0.1% v/v formic acid in acetonitrile (phase B). The gradient program was a total of 16.5 min. The initial mobile phase B was set at 5% at a flow rate of 0.3 mL min^−1^. The proportion of mobile phase B increased linearly from 5% to 9% over 2 min. In the following 5.9 min, phase B increased to 9.5%, then sharply increased to 20% over 0.3 min. After holding at 20% for 3 min at a flow rate of 0.6 mL min^−1^, the proportion of phase B decreased to 5% in 0.2 min and holding on for 3 min. Subsequently, the flow rate decreased into 0.3 mL min^−1^ in 0.1 min followed by an equilibration time of 2 min. Extraction procedure, retention times, parameters of multiple reaction monitoring, matrix effect, linearity and sensitivity, absolute recoveries, interday and intraday precision were all described in Shahid et al.^[^ ^30^ ^]^

### Amino Acid Profiling

Young and mature seeds were collected from KN5585 and transgenic maize. Free amino acids were analyzed with a Hitachi amino acid analyzer L‐8900 (https://www.techcomp.cn/) according Chinese national specification for determination of amino acids in foods (GB 5009.124‐2016).^[^ ^75^ ^]^

### Sequence Alignment

Phylogenetic evolutionary analyses were conducted using MEGA version 11.^[^ ^76^ ^]^ The amino acid sequences of *GFT* in maize and other species were aligned using ClustalW (https://www.genome.jp/tools‐bin/clustalw), and output by Espript3.0 http://espript.ibcp.fr/ESPript/cgi‐bin/ESPript.cgi).^[^ ^77^ ^]^ The secondary structure model of *AtGFT* was downloaded from AlphaFold Protein Structure Database (https://alphafold.ebi.ac.uk/).^[^ ^37^ ^]^ The conserved domain analysis was annotated using The Conserved Domain Database in NCBI (Batch Web CD‐Search Tool, https://www.ncbi.nlm.nih.gov/Structure/bwrpsb/bwrpsb.cgi).

### Purification of *ZmGFTs*, *SbGFTs*, *PaGFT*, *TaGFT*, *StGFT*, *SsFTCD*, and *SsFT* Proteins

*ZmGFT* genes were amplified from the cDNAs of *Qi319* and *B73* and subcloned into a modified pFastBac1 vector with a 10 × His affinity tag fused to the C‐terminus, respectively. Bacmids were generated in DH10Bac cells in accordance with the instructions for the Bac‐to‐Bac baculovirus expression system (USA, https://www.thermofisher.cn/cn/zh/home/brands/invitrogen.html). Baculovirus was generated and amplified in Sf‐9 insect cells.^[^ ^78^ ^]^ Variant *ZmGFT* proteins were expressed in Sf‐9 insect cells at 27 °C for 60 h using individual viruses. Cells were harvested by centrifugation at 2000 × *g* for 15 min and homogenized in ice‐cold lysis buffer containing 25 mm Tris‐HCl (pH 8.0), 150 mm NaCl, and 0.5 mm phenylmethanesulfonyl‐fluoride. Cells were disrupted using a cell homogenizer (China, http://www.xajuneng.cn/). The insoluble fraction was precipitated by ultracentrifugation (20 000 × *g*) for 1 h at 4 °C. The supernatant was loaded onto a Ni‐NTA superflow affinity column (German, https://www.qiagen.com/) and washed 3 times with lysis buffer plus 10 mm imidazole. Elution was performed in buffer containing 25 mm Tris‐HCl (pH 8.0), and 250 mm imidazole. The protein was further purified using Source15Q (USA, https://www.gehealthcare.com/) and concentrated to ≈1 mg mL^−1^ (Amicon 30 kDa cutoff, USA, https://www.merckmillipore.com/), followed by SEC (Superdex‐200 Increase 10/300; USA, https://www.gehealthcare.com/) and equilibrated with 25 mm Tris‐HCl (pH 8.0), 150 mm NaCl, and 5 mm dithiothreitol. Peak fractions were pooled for the enzymatic assay. Mutant *ZmGFT* genes were constructed by overlap polymerase chain reaction and the proteins were expressed and purified as description above.

The genes of *SbGFT*, *PaGFT*, *TaGFT*, *StGFT*, and *SsFTCD* were synthesized by Genewiz (China, https://www.genewiz.com.cn/) and optimized for expression in *E. coli*. The sequence of *SsFT* was amplified by polymerase chain reaction using the *SsFTCD* gene as the template. All genes were individually subcloned into a modified PeT21b vector with a 6 × His tag fused at the C terminus (USA, https://www.novagen.com/), and the plasmid was transformed into BL21 (DE3) cells. One liter of lysogeny broth medium supplemented with 100 µg mL^−1^ ampicillin was inoculated with a transformed bacterial preculture and shaken at 37 °C until the optical density reached 1.0 at 600 nm. After induction with 0.2 mm isopropyl‐β‐d‐thiogalactoside and 16 h of growth at 16 °C, the bacterial cells were collected and homogenized in a buffer containing 25 mm Tris‐HCl (pH 8.0), and 150 mm NaCl, then centrifugated at 23 000 × *g* at 4 °C. The supernatant was loaded onto a column equipped with Ni^2+^ affinity resin (Ni‐NTA, Qiagen), washed with a buffer containing 25 mm Tris‐HCl (pH 8.0), 150 mm NaCl, 15 mm imidazole, and eluted with a buffer containing 25 mm Tris‐HCl (pH 8.0) and 250 mm imidazole. The eluted protein was applied to Source15Q (USA, https://www.gehealthcare.com/), then subjected to gradient NaCl elution (up to 1 m) in 25 mm Tris‐HCl (pH 8.0). The elution peak was concentrated to 1 mL (≈10 mg mL^−1^) and subjected to gel filtration chromatography (Superdex200 Increase 10/300; USA, https://www.gehealthcare.com/) equilibrated with a buffer containing 25 mm Tris‐HCl (pH 8.0), 150 mm NaCl, and 5 mm 1,4‐dithiothreitol. The peak fractions were collected for enzymatic activity determination or crystallization trials. The primers used in this experiment are listed in Table S17 (Supporting Information).

### Determination of *GFT* Activity In Vitro

MeFox powder was purchased from Toronto Research Chemicals (Canada, http://www.trc‐canada.com), and other folate derivative powder was purchased from Schircks Laboratories (Switzerland, http://www.schircks.ch), and resolved in a buffer containing 50 mm sodium phosphate (pH 7.0) and 10 mm β‐mercaptoethanol as the standard sample for mass spectrometry determination or substrate for enzymatic activity measurement. *N*‐formimino‐l‐glutamate powder was purchased from Sigma‐Aldrich (China, https://www.sigmaaldrich.com/china‐mainland.html).

For the *formiminotransferase* activity assay,^[^ ^33^ ^]^ a 0.1 mL mixture containing 0.1 m phosphate buffer (pH 7.4), 10 mm β‐mercaptoethanol, 0.5 mm THF, 5 mm *N*‐formimino‐l‐glutamate, and tested proteins (10, 5, 2 µm for *SsFTCD* or *SsFT*, 10 µm for *SbGFT*, *TaGFT*, *PaGFT*, *StGFT*, *ZmGFT*–*B73*, and *ZmGFT*–*Qi319*, respectively) was incubated for 2 h at 30 °C. The reaction was stopped by the addition of 0.1 mL of 0.36 m HCl. The tubes were then heated at 100 °C for 55 s, cooled on ice, and centrifuged to remove the precipitant. The absorbance at 350 nm was determined by comparison with a blank which did not contain formiminoglutamate.

For mass spectrometry assays, MeFox and 5‐M‐THF were detected using multiple reaction monitoring in the ESI positive mode on a mass spectrometer. Multiple reaction monitoring was used to identify MeFox and 5‐M‐THF.^[^ ^34^ ^]^ Standard curves of MeFox or 5‐M‐THF were drawn by measuring the gradient concentration of the sample in 10 parts per billion (ppb: µg L^−1^), 20, 40, and 80 ppb, respectively. One millimolar 5‐M‐THF substrate was incubated with *SsFTCD* (0.8 µm), *SsFT* (0.8 µm), and a gradient plant *GFT* protein (0.8, 0.4, and 0.2 µm, respectively) in 100 µL reaction buffer {50 mm sodium phosphate (pH 7.0) and 10 mm β‐mercaptoethanol} at 30 °C for 4 h, respectively. The reaction was then diluted by tenfold and quenched with a buffer containing 50% methanol v/v, 0.1% sodium ascorbic acid w/v, 0.5% β‐mercaptoethanol v/v, and 20 mm ammonium acetate. A 2 µL reaction mixture was used for liquid chromatography (UFLC SHIMADZU CBM20A system; Japan, https://www.labx.com/product/shimadzu‐uflc) with a C18 column (VP‐ODS, 150 L × 20; Japan, https://www.labx.com/product/shimadzu‐uflc) and mass spectrometry (Applied Biosystems 4000 Q TRAP; USA, https://www.labx.com/product/applied‐biosystems‐api) to detect MeFox production or 5‐M‐THF consumption. The amounts of the product generated (or substrate remaining) during the reaction were calculated based on the peak areas.

For *GFT* converting assay, 0.5 µm protein was incubated at 30 °C with 20–900 µm of 5‐M‐THF substrate in a 100 µL reaction buffer containing 50 mm sodium phosphate (pH 7.0) and 10 mm β‐mercaptoethanol at 30 °C. Blank represented a reaction containing all necessary ingredients but the *GFT* protein. The reaction was quenched and subjected to liquid chromatography–mass spectrometry as described above. The initial velocity of the reaction was calculated by measuring MeFox generation at 3, 6, 9, or 12 min, respectively. The initial velocity in a gradient concentration of 5‐M‐THF was fitted by the Michaelis–Menten equation to obtain the *k*_cat_ and *K*_m_ values in GraphPad Prism software (GraphPad Software, Inc, USA; https://www.graphpad.com/). *k*_cat_/*K*_m_ values were then generated by calculation.

To figure out what could be the substrate and the product of *GFT* conversion, two types of reactions were prepared: the test group and the control group. The control group contained 1 mm 5‐M‐THF as the substrate in the reaction buffer and was incubated at 30 °C for 2 h. The test group incubated 1 mm 5‐M‐THF with *SbGFT* (0.8 µm), *PaGFT* (0.8 µm), or *TsGFT* (0.8 µm) in the reaction buffer at 30 °C for 2 h, respectively. When the time reached 2 h, the reactions were stopped by diluted tenfold and quenched with a termination buffer containing 50% methanol v/v, 0.1% sodium ascorbic acid w/v, 0.5% β‐mercaptoethanol v/v, and 20 mm ammonium acetate. The supernatant was subjected to 3 kDa ultra filtration and 100µL extracts were transferred to a glass amber vial featuring a microinsert for UHPLC (Thermo Vanguish Flex, USA, https://www.thermofisher.com/)–QTOF‐MS/MS (SCIEX TripleTOF 6600+, Canada, https://www.sciex.com) analysis. The injection volume was 10µL, the column was ACQUITY UPLC CSH C18 (130Å, 1.7 µm, 2.1 mm × 100 mm, Waters, USA, https://www.waters.com); solvent A was purified water with 0.1% formic acid, and solvent B was acetonitrile with 0.1% formic acid. The parameters of UHPLC were following: 0.3 mL min^−1^ of 95% solvent A/5% solvent B at 0 min, 0.3 mL min^−1^ of 91% A/9% B at 2 min, 0.4 mL min^−1^ of 90.5% A/9.5% B at 7.9 min, 0.4 mL min^−1^ of 80% A/20% B at 8.2 min, 0.4 mL min^−1^ of 80% A/20% B at 11.2 min, 0.4 mL min^−1^ 5% A/95% B at 11.3 min, 0.4 mL min^−1^ of 5% A/95% B at 14.3 min, 0.4 mL min^−1^ of 95% A/5% B at 14.5 min, 0.3 mL min^−1^ of 95% A/5% B at 16.5 min, 0.3 mL min^−1^ of 95% A/5% B at 16.6 min, 0.3 mL min^−1^ of 95% A/5% B at 18 min. Column temperature was 25 °C. For MS detection, capillary voltage was +3.50 kV when positive polarity was used. Cone voltage was 25.0 V, source temperature was 500 °C, declustering potential was: 80.0 V, collision energy was 30.0 V, and collision energy spread was 15.0 V, respectively. Mass range was 50 to 1000 *m*/*z*, the minimum TOF masses was 100 Da, and the maximum TOF masses max was 1000 Da, scan duration was 15.507 min, mass tolerance: 50 ppm, data acquisition was centroid mode, scan duration was 1.0 s, and interscan delay was 0.1 s, respectively. The retention time of 5‐M‐THF, MeFox, and the unknown intermediate was 1.45, 4.25, and 3.34 min, respectively. To figure out if MeFox was produced when 5‐M‐THF was incubated alone even in the absence of antioxidants, the reaction at various time points between 0 and 2 h was immediately loaded for UHPLC–QTOF‐MS/MS analysis without adding termination buffer.

### Crystallization of *SbGFT*

*SbGFT* (A6‐D317, C260S/C314S, C‐His) was crystallized using the sitting‐drop vapor‐diffusion method at 18 °C by mixing 1 mL of the sample with an equal volume of reservoir solution. Crystal optimization was carried out by hanging drop plate. After the pH buffer, salt concentration, and additive screening, high‐resolution crystals were obtained in the conditions containing 15% w/v polyethylene glycol 20 000, 0.1 m HEPES (pH 7.0), and 3% w/v 1.6‐hexanediol. The crystals were flash‐frozen in liquid nitrogen using 20% v/v ethylene glycol as the cryoprotective buffer and diffracted to 1.75 Å at the Shanghai Synchrotron Radiation Facility beamline BL17U1.^[^ ^79^ ^]^ The structure of *SbGFT* was resolved by the isomorphous replacement method using the selenomethionine method.

### Data Collection and Structural Determination

All data sets were collected at the Shanghai Synchrotron Radiation Facility beamline BL17U1 or BL19U and processed with the HKL3000 or HKL2000 packages.^[^ ^80^ ^]^ Further processing was performed with programs from the CCP4 suite.^[^ ^81^ ^]^ Data collection and structure refinement statistics are summarized in Table S7 (Supporting Information). The structure was manually iteratively refined with the PHENIX^[^ ^82^ ^]^ and COOT^[^ ^83^ ^]^ tools. All figures were generated using the PyMOL software (http://www.pymol.org).

### Structure Modeling

The X‐ray crystal structure of *SbGFT* was used as the template to construct the 3D model of *ZmGFT* because they shared the high amino acid sequence identity. In addition, a blast search^[^ ^84^ ^]^ of the amino acid sequence of *ZmGFT* was conducted against the current PDB (http://www.rcsb.org) to obtain further information. The *FT* domain of *SsFTCD* (PDB: 1QD1) was chosen as the reference because the crystal structure of the *SsFT* complex contained an analog molecule, 6R‐5‐F‐THF. Several initial models were constructed using the Modeler module^[^ ^85^ ^]^ in Discovery Studio 2.0 (Accelrys Software Inc.), and the model with the highest Profiles‐3D^[^ ^86^ ^]^ score was retained. Energy minimization procedures were processed under the CHARMm^[^ ^87^ ^]^ force field. The SHAKE algorithm^[^ ^88^ ^]^ was applied to constrain covalent bonds to hydrogen atoms during the minimization. Finally, the Profiles‐3D method was used to evaluate the fitness between the sequence and the current 3D model. *ZmGFT*–*B73* and *ZmGFT*–*Q319* were modeled separately. In addition, another structural model of *ZmGFT*–*B73* predicted by Alphafold was obtained in AlphaFold Protein Structure Database (https://alphafold.ebi.ac.uk/).^[^ ^37^ ^]^

### Molecular Docking

Using the established homology model, AutoDock Vina^[^ ^89^ ^]^ was employed to find the potential ligand of *ZmGFT* as well as the binding mode between ligand and protein. The 3D structures of folate derivatives (Table S13, Supporting Information) were sketched and further refined with the steepest descent minimization for 2000 steps, followed by gradient minimization for another 2000 steps, using the CHARMm force field. The active site pocket of the receptor was identified by Discovery Studio 2.0, and the location of the ligand 6R‐5‐F‐THF in 1DQ1 was referenced for additional information. A box size of 20 × 18 × 18 was set as the entire binding pocket using ADT software.^[^ ^90^ ^]^ Other parameters were set as default. The top nine docking positions ranked by the binding affinity were preserved to find the most probable binding mode. To investigate the binding abilities of 5‐M‐THF to *ZmGFT*–*B73* and *ZmGFT*–*Qi319*, four docking programs (i.e., AutoDock Vina,^[^ ^89^ ^]^ AutoDock 4,^[^ ^90^ ^]^ CDOCKER,^[^ ^91^ ^]^ and LigandFit^[^ ^92^ ^]^) were employed to find the best binding mode. The binding pockets were defined as above. The top docking scores of each program were collected to generate a consensus score.

### Molecular Dynamic Simulation

The molecular dynamics simulations of the complex *B73* and 5‐M‐THF, *B73* and 5‐F‐THF, *B73* and THF, and *Qi319* and 5‐M‐THF, respectively, were performed using GROMACS 2023.^[^ ^90^ ^]^ The simulation system was placed in a dodecahedral periodic cell with water molecules. After hydrogenation and energy minimization step, the system was gradually heated to 300 K in CHARMM36 force filed,^[^ ^93^, ^94^, ^95^, ^96^, ^97^ ^]^ the optimized complex was subjected to a 100 ps *NVT* ensemble equilibration followed by a 100 ps *NPT* ensemble equilibration at 1 bar pressure, and a 10 ns production of MD simulation was performed. All structures were simulated individually and the binding free energy of each complex was determined using MMPBSA method. The key residues contributed for binding of protein–ligand was calculated by GMXMMPBSA.^[^ ^98^, ^99^ ^]^ Average total binding energy through three simulations, along with the energy components, are presented in Figure S11E (Supporting Information).

### Accession Numbers

Gene resequencing data were available under GenBank accession codes KT727273‐KT727912. Isolated coding sequences of *ZmGFT* in maize inbred lines *B73* and *Qi319* were available under GenBank accession codes KT727913 and KT727914, respectively. *ZmGFT* (*ZmGFT*–*B73*), protein sequence from *Z. mays* inbred line *B73*, NP_001130076.1; *ZmGFT*–*Qi319*, protein sequence from *Z. mays* inbred line *Qi319*, AMK92167.1; *SbGFT*, protein sequence from *Sorghum bicolor*, XP_002466878.1; the atomic coordinates and structure factors for the reported crystal structures were deposited in the PDB (http://www.rcsb.org) with the accession codes 7DYH. *TaGFT*, sequence from *T. aestivum*, KAF6990789.1; *OsGFT*, sequence from *O. sativa*, XP_015633257.1; *PaGFT*, sequence from *P. avium*, XP_021832372.1; *StGFT*, sequence from *S. tuberosum*, XP_006357514.1; *LaGFT*, sequence from *L. annua*, txid153659; *AtGFT*, sequence from *A. thaliana*, NP_973497.1; *CvGFT*, sequence from *C. variabilis*, XP_005850624.1; *VcGFT*, sequence from *V. carteri*, XM_002946226.1; *SmGFT* sequence from *S. moellendorffii*, XP_002964559.1; *GkGFT*, sequence from *G. kilaueensis*, WP_023175819.1; *EcGFT*, sequence from *E. coli*, MZZ90505.1; *SsFTCD*, sequence from *S. scrofa*, NP_999440.1; *HsFTCD*, sequence from *H. sapiens*, NP_006648.1; *MnFTCD*, sequence from *M. musculus*, NP_543121.1; *RnFTCD*, sequence from *R. norvegicus*, NP_446019.1.

### Statistical Analysis

The statistical analysis of GWAS was detailed in the preceding section. Phenotypic data, including plant height, ear height, hundred‐seed weight, rows per ear, starch content, and protein content, were based on 20 biological replicates and expressed as the mean ± standard error of the mean. Folate data from wild type, CRISPR mutants, and overexpression lines were obtained from three to five biological replicates and expressed as the mean ± standard error of the mean. RNA‐seq data were obtained from two biological replicates of each group. Folate data from sweet corn were obtained from three biological replicates (two independent rounds of detection) and expressed as the median and IQR. The enzyme activity assay experiments were repeated at least 2 times to ensure reproducible outcomes. Statistical differences between experimental groups were typically evaluated using Student's *t*‐test, nonparametric Mann–Whitney *U* test, or one‐way ANOVA. A *p*‐value threshold of less than 0.05 was considered statistically significant. Statistical analysis was carried out using GraphPad Prism 9.5.0.

## Conflict of Interest

The authors declare no conflict of interest.

## Author Contributions

T.L., W.G., Y.W., W.W., and W.X.W. contributed equally to this work. P.Y., D.Z., J.Y., and C.Z. designed and supervised this study. T.L., W.G., W.X.W., L.J., Q.L., and J.L. performed the folate measurement and TOF analysis. T.L., W.G., W.W., and H.L. performed GWAS data analysis. T.L., L.J., Q.L., J.L., L.P., and Q.Q.L. performed the analysis on transgenic plants. Y.W., L.J., J.L., Y.X., P.Y., and D.Z. performed protein analysis in vitro. W.X.W. and J.L. performed molecular simulation analysis. T.L., W.W., W.X.W., L.J., D.Z., J.Y., and C.Z. prepared the paper with inputs from other authors. All authors read and commented on the paper.

## Supporting information



Supporting Information

Supplemental Figure 1

Supplemental Figure 2

Supplemental Figure 3

Supplemental Figure 4

Supplemental Figure 5

Supplemental Figure 6

Supplemental Figure 7

Supplemental Figure 8

Supplemental Figure 9

Supplemental Figure 10

Supplemental Figure 11

Supplemental Figure 12

Supplemental Figure 13

Supplemental Figure 14

Supplemental Figure 15

Supplemental Figure 16

Supplemental Figure 17

Supplemental Figure 18

Supplemental Table 1

Supplemental Table 2

Supplemental Table 3

Supplemental Table 4

Supplemental Table 5

Supplemental Table 6

Supplemental Table 7

Supplemental Table 8

Supplemental Table 9

Supplemental Table 10

Supplemental Table 11

Supplemental Table 12

Supplemental Table 13

Supplemental Table 14

Supplemental Table 15

Supplemental Table 16

Supplemental Table 17

Supplemental DataFile

## Data Availability

The data that support the findings of this study are available in the supplementary material of this article.
