# Supplementary material for: An Allele of Glutamate Formiminotransferase Triggers 5‐Methyl‐Tetrahydrofolate‐to‐MeFox Conversion and Facilitates Folate Biofortification in Maize
Source: Adv Sci (Weinh). 2025 Aug 21;12(42):e15082. doi: 10.1002/advs.202415082 (PMC12622545; doi:10.1002/advs.202415082)
Supplement: Supplementary file 1 — Supporting Information [file ADVS-12-e15082-s022.docx]

**Supporting Information**

# An Allele of Glutamate Formiminotransferase triggers 5-methyl-tetrahydrofolate-to-MeFox conversion and facilitates folate biofortification in maize

Tong Lian^1,2,3,6#^, Wenzhu Guo^4#^, Yanjing Wang^4#^, Weiwei Wen^4#^, Weixuan Wang^1,3#^, Ling Jiang^1^, Qiuju Liang^1,3^, Ji’an Liu^1^, Haijun Liu^4^, Yuan Xue^4^, Lixu Pan^5^, Qiaoquan Liu^5^, Ping Yin^4^, Delin Zhang^4^*, Jianbing Yan^4^*, Chunyi Zhang^1,2,3^*

*Correspondence should be addressed to Delin Zhang (zdl@mail.hzau.edu.cn), Jianbing Yan ([yjianbing@mail.hzau.edu.cn](mailto:yjianbing@mail.hzau.edu.cn)), and Chunyi Zhang (zhangchunyi@caas.cn)

^#^These authors contributed equally to this work

**
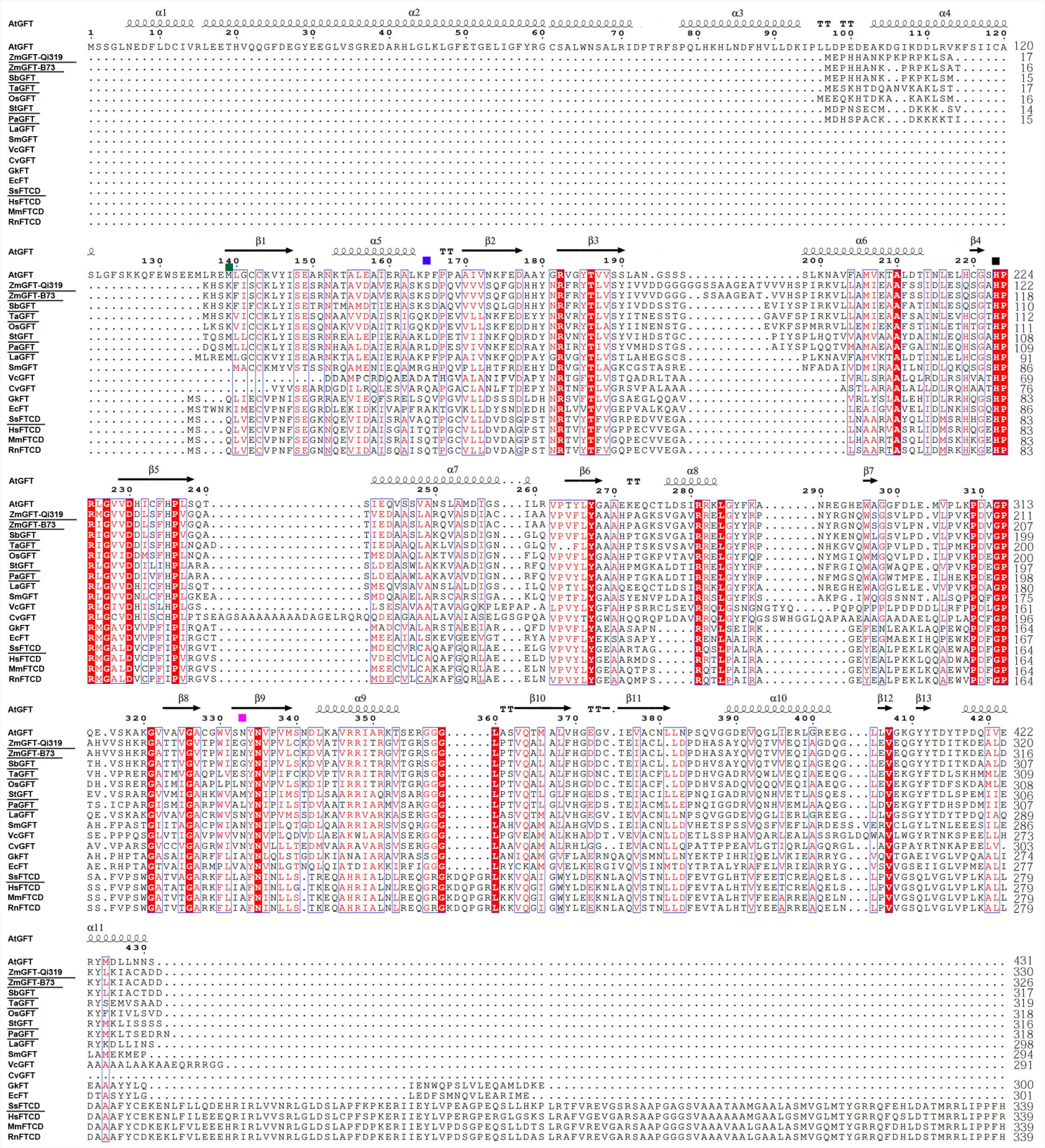
**

**Figure S1. Protein sequence alignment of GFT orthologs from plants and mammals.** On the bottom of protein sequences, three square symbol labeled different sites in the results. Dark green square symbol represents the changed serine (S) at position 20 where the peptide started to change due to the gene-editing in *ZmGFT*-editing protein. The blue square symbol represents the stop-gain mutation site at position 46. Black square symbol represents histidine (H) at position 117 in ZmGFT-B73 that was mutated for the activity assay. The pink square symbol represents asparagine (N) at position 228 (corresponding to S1789) in ZmGFT-B73 and glycine (G) at position 232 in ZmGFT-Qi319, aligning with alanine (A) at position 184 in SsFTCD, HsFTCD, MnFTCD, and RnFTCD. ZmGFT-B73, sequence from maize (*Zea mays*) inbred line B73, NP_001130076.1; ZmGFT-Qi319, sequence from maize inbred line Qi319, AMK92167.1; SbGFT, sequence from *Sorghum bicolor*, XP_002466878.1; TaGFT, sequence from *Triticum aestivum*, KAF6990789.1; OsGFT, sequence from *Oryza sativa*, XP_015633257.1; PaGFT, sequence from *Prunus avium*, XP_021832372.1; StGFT, sequence from *Solanum tuberosum*, XP_006357514.1; LaGFT, sequence from *Lunaria annua*, Luann.0189s0003.1; AtGFT, sequence from *Arabidopsis thaliana*, NP_973497.1; CvGFT, sequence from *Crucigenia variabilis*, XP_005850624.1; VcGFT, sequence from *Volvox carteri*, [XM_002946226.1](https://www.ncbi.nlm.nih.gov/nuccore/XM_002946226.1); SmGFT sequence from *Selaginella moellendorffii*, XP_002964559.1; GkGFT, sequence from *Gloeobacter kilaueensis*, WP_023175819.1; EcGFT, sequence from *Escherichia coli*, MZZ90505.1; SsFTCD, sequence from *Sus scrofa*, NP_999440.1; HsFTCD, sequence from *Homo sapiens*, NP_006648.1; MnFTCD, sequence from *Mus musculus*, NP_543121.1; RnFTCD, sequence from *Rattus norvegicus*, NP_446019.1. The proteins used in this study are underlined.

**
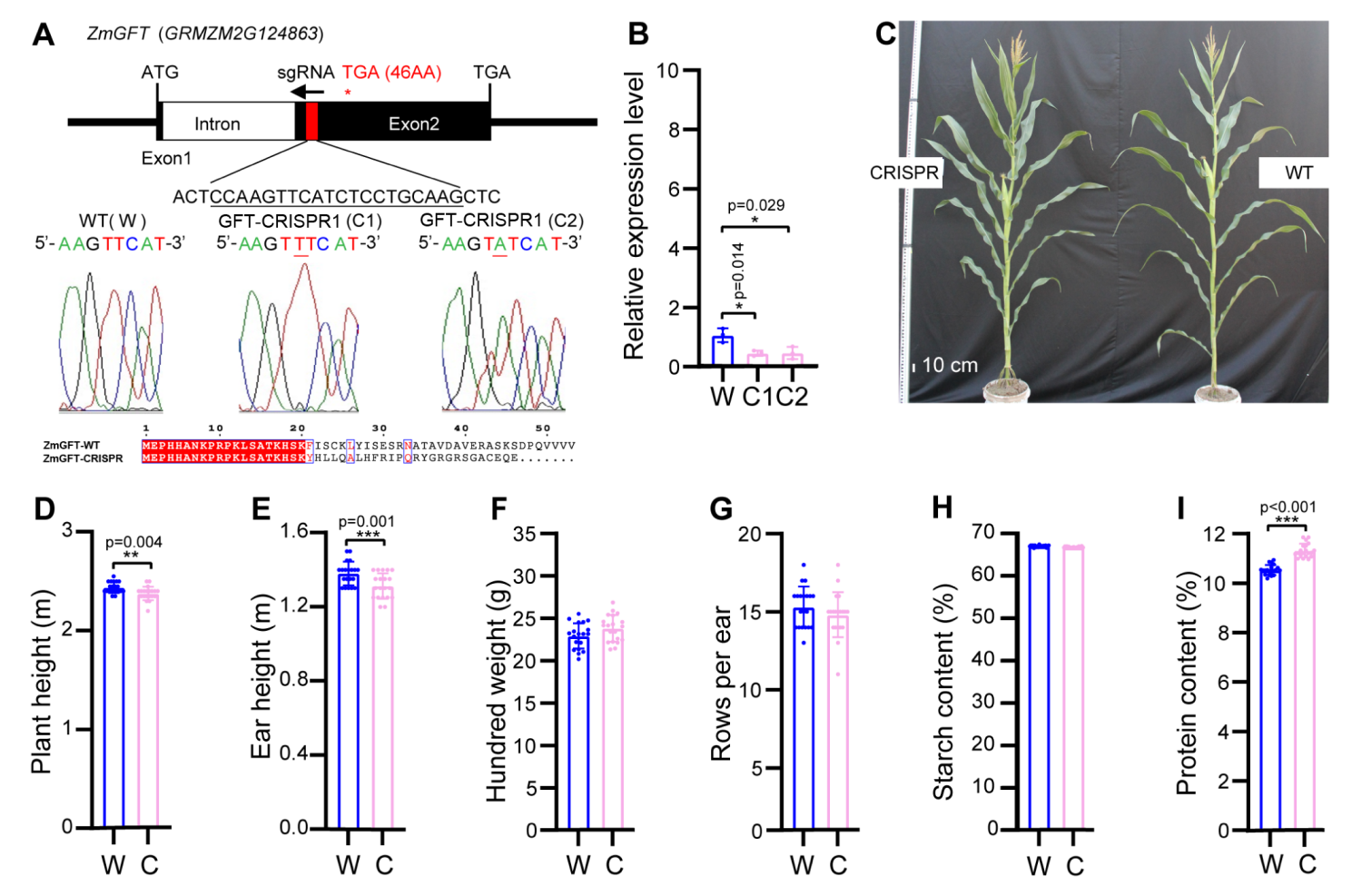
**

**Figure S2. Phenotypic characterization of *ZmGFT*-editing.** A) Gene structure of the *ZmGFT* and sgRNA target site in exon 2. The sgRNA target sequence is underlined. TGA, indicated by an asterisk, represents the resulting stop-gain mutation at the 62^th^ bp of the coding region corresponding to the stop at 47^th^ amino acid due to the insertion T for Crispr1 (C1) and A for Crispr2 (C2), respectively, highlighted by red underlines. The alignment between the truncated amino acid sequence of the Crispr mutant and the first 50 amind acid sequence of the wild type. B) Relative expression levels of in young seeds (DAP 25) from wild-type (WT, W) maize and *ZmGFT*-editing (Crispr 1 and 2) as mean ± SD of three biological replicates. Error bars represent standard deviations. C) Photos of WT and CAS9-free Crispr plants at DAS 70. D) Plant height of WT and Crispr plants at DAS 70. E) Ear height of WT and Crispr plants at DAS 70. F) Weight of hundred seeds from WT and Crispr plants. G) Rows per ear from WT and Crispr plants. H) Starch content of mature seeds from WT and Crispr plants. I) Protein content of mature seeds from WT and Crispr plants. Plant height, ear height, hundred-seed weight, rows per ear, starch content and protein content are presented as mean ± SD of twenty biological replicates, respectively. *p*-values are shown (Student’s *t*-test, *, *p* < 0.05; **, *p* < 0.01; ***, *p* < 0.001). Error bars represent standard deviations. Source data for Figure S2B,D,F,G,H,I are provided in the Source Data file.


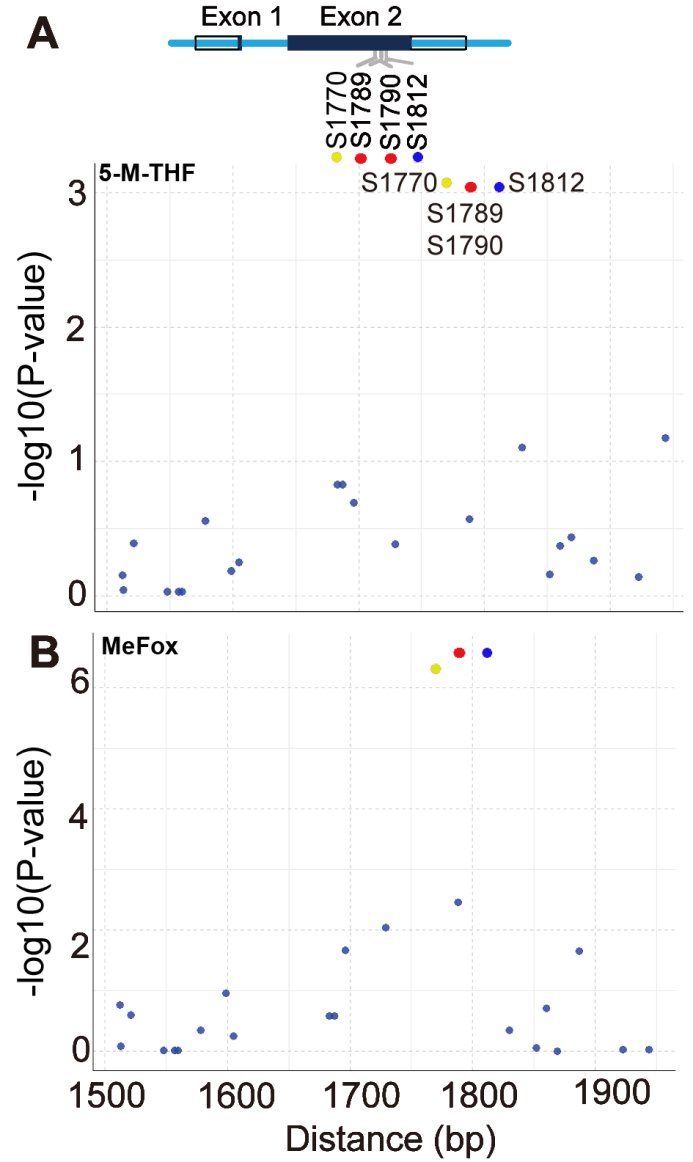


**Figure S3. Association analysis results of *ZmGFT* SNPs within a genomic region for 5-M-THF and MeFox.** A) For 5-M-THF: the plot shows the negative log-transformed p-values for SNPs across a range of distances (measured in base pairs, bp). Higher values indicate stronger associations with the 5-M-THF. B) For MeFox: Higher values indicate stronger associations with the MeFox; Significant SNPs (S1770, S1789, S1790, and S1812) are highlighted in yellow, red and blue, respectively, suggesting these SNPs may have a notable association with 5-M-THF and MeFox.


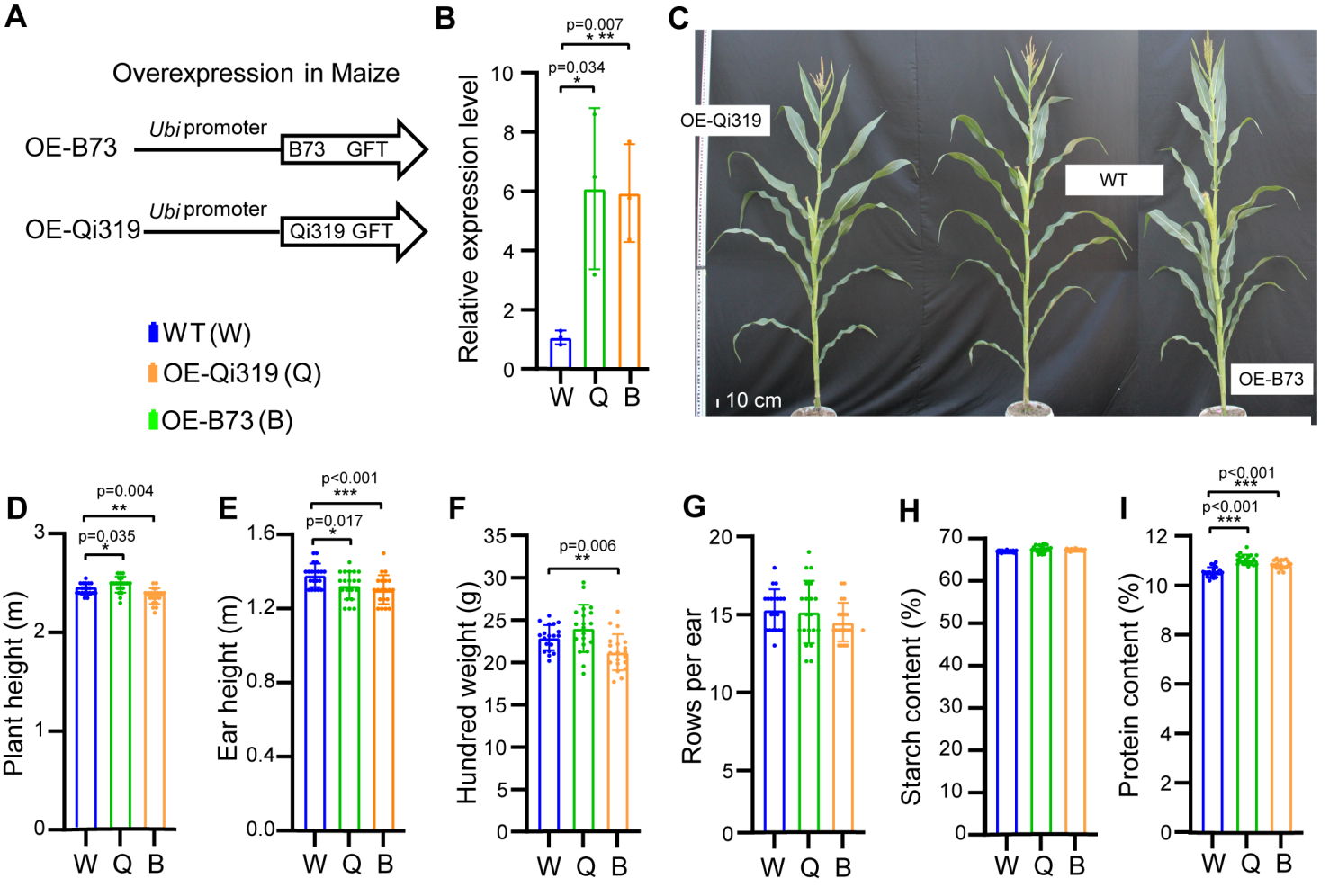


**Figure S4. Phenotypic characterization of *ZmGFT*-overexpressing maize plants.** A) Constructs used for *ZmGFT* overexpression driven by the maize ubiquitin promoter. OE-B73, *ZmGFT-B73* overexpressor; OE-Qi319, *ZmGFT-Qi319* overexpressor. B) Relative expression levels of in young seeds (DAP 25) from wild-type (WT, W) maize and and overexpressors either with the overexpression of *ZmGFT-Qi319* (OE-Qi319, Q) or *ZmGFT-B73* (OE-B73, B) as mean ± SD of three biological replicates. Error bars represent standard deviations. C) Photos of wild-type maize and overexpressors at DAS 70. Inbred line KN5585, the genetic transformation recipient, was used as the wild type (WT), and the endogenous *GFT* gene carries G at S1789. D) Plant height of WT, OE-Qi319, and OE-B73 plants at DAS 70. E) Ear height of WT, OE-Qi319, and OE-B73 plants at DAS 70. F) Weight of hundred seeds from WT, OE-Qi319, and OE-B73 plants. G) Rows per ear from WT, OE-Qi319, and OE-B73 plants. H) Starch content of mature seeds from WT, OE-Qi319, and OE-B73 plants. I) Protein content of mature seeds from WT, OE-Qi319, and OE-B73 plants. Plant height, ear height, hundred-seed weight, rows per ear, starch content and protein content are presented as mean ± SD of twenty biological replicates, respectively. *p*-values are shown (Student’s *t*-test, *, *p* < 0.05; **, *p* < 0.01; ***, *p* < 0.001). Error bars represent standard deviations. Source data for Figure S4B,D,F,G,H,I are provided in the Source Data file.


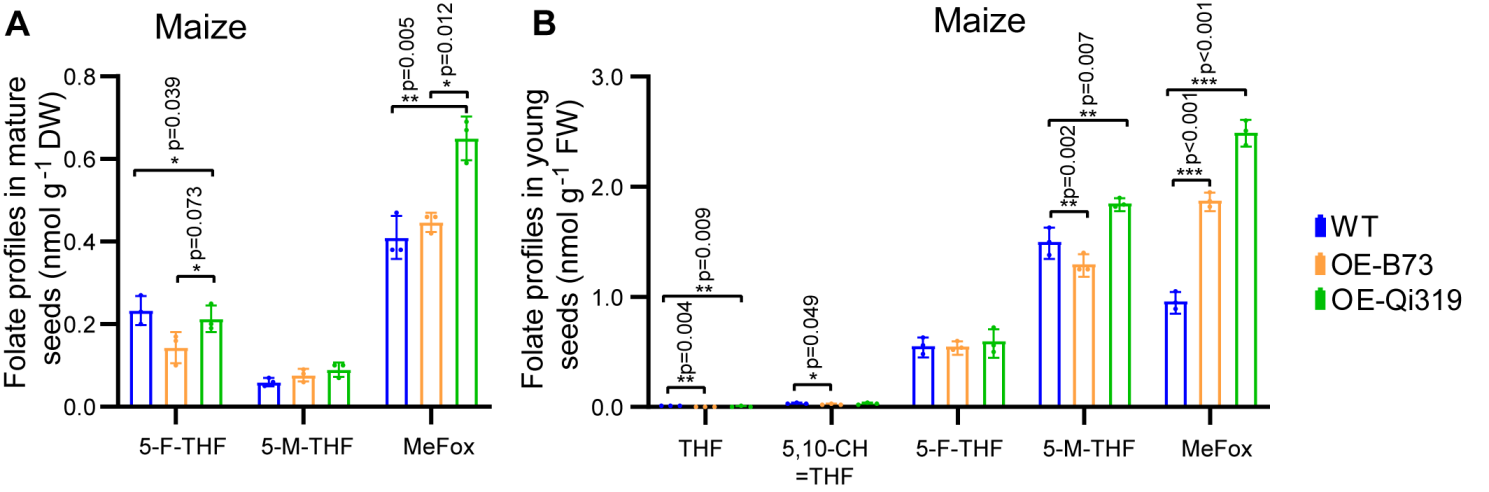


**Figure S5. Effects of *GFT* overexpression on folate accumulation in plants.** A) Folate profiles (nmol g^-1^ DW) in mature seeds from the WT, overexpressor of *ZmGFT-Qi319* (OE-Qi319) or *ZmGFT-B73* (OE-B73). B) Folate profiles (nmol g^-1^ FW) in young seeds from the WT, OE-Qi319 and OE-B73. Accumulated folate derivatives are presented as mean ± SD of three biological replicates, respectively. *p*-values are shown (Student’s *t*-test, *, *p* < 0.05; **, *p* < 0.01; ***, *p* < 0.001). Error bars represent standard deviations. Source data for Figure S5A,B are provided in the Source Data file.


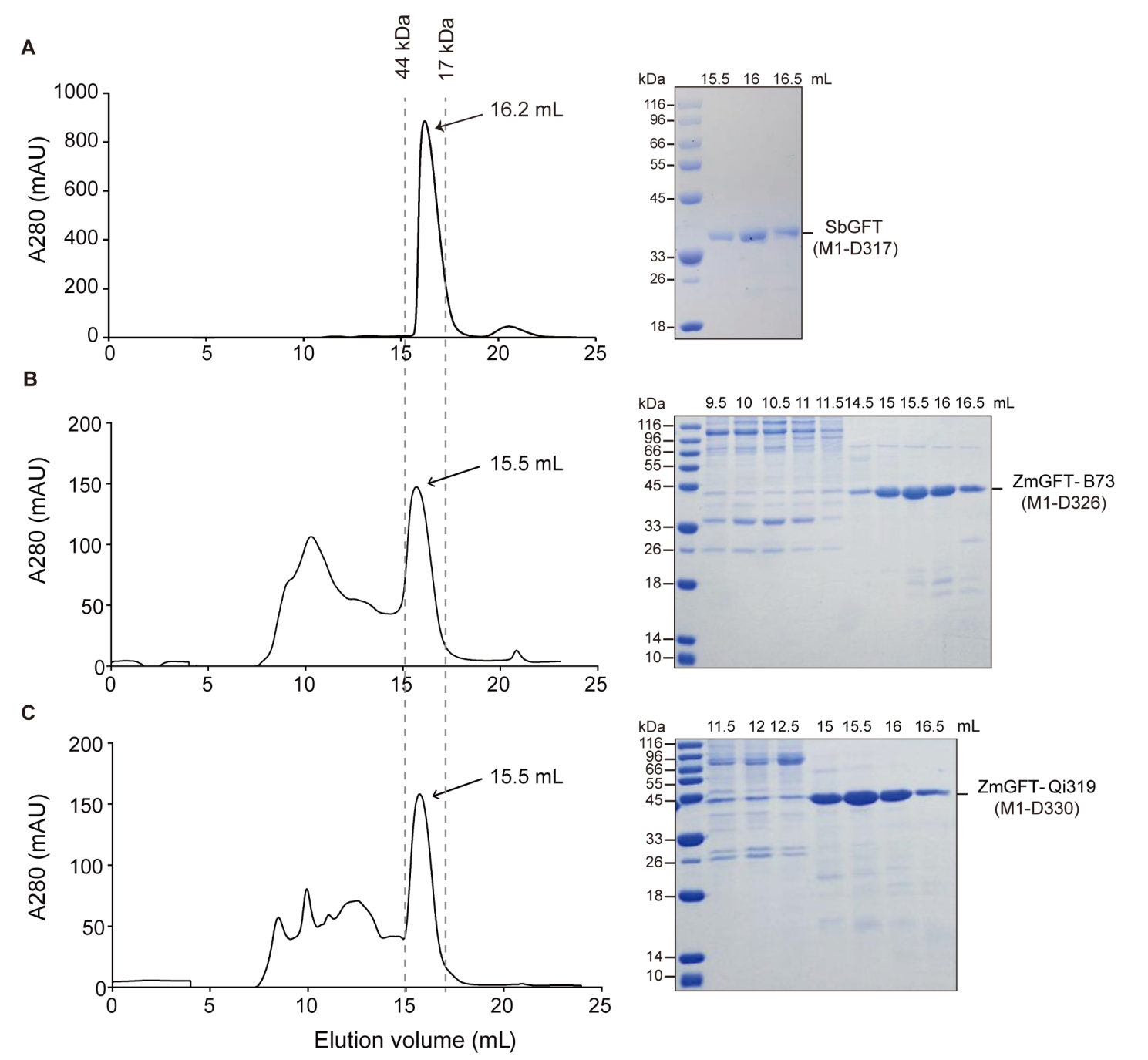


**Figure S6. Size-exclusion chromatography of the GFT proteins including *Zea mays* and *Sorghum bicolor*.** A) Representative gel filtration chromatography and 15% SDS-PAGE photo from the purification of the SbGFT protein. B) Representative gel filtration chromatography and 15% SDS-PAGE photo from the purification of the ZmGFT-B73 protein. C) Representative gel filtration chromatography and 15% SDS-PAGE photo from the purification of the ZmGFT-Qi319 protein. The peaks containing the target protein are illustrated by a black arrow. Plant GFT proteins appear to elute at ~16 ml of roughly 17,000 to 44,000 molecular weight, indicating that GFT proteins exist as monomer in solution. Source data for Figure S6 are provided in the Source Data file.


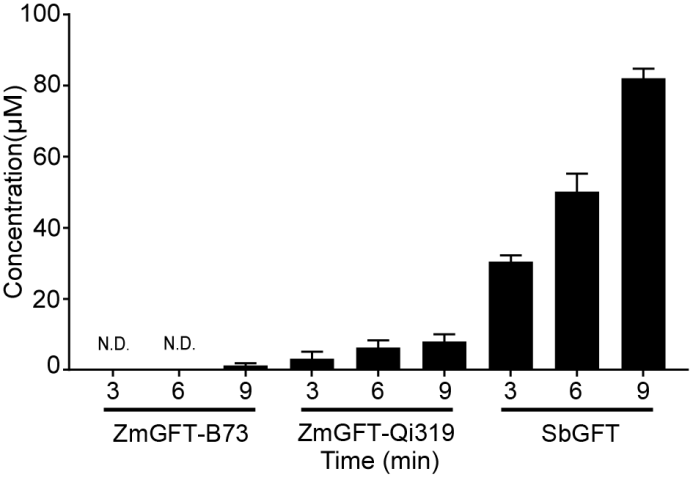


**Figure S7.** **The concentration of MeFox after the incubation of 5-M-THF with plant GFTs resulted at 0, 3, 6, and 9 minutes (min) *in vitro*.** After 5-M-THF incubated with GFTs from *Sorghum bicolor* and *Zea mays* for 3, 6 or 9 minutes, the quantification of the production MeFox is detected by HPLC–MS/MS. The concentration of the MeFox increased in a time-dependent manner. Each bar represents mean ± standard error of mean, n = 2. Source data for Figure S7 are provided in the Source Data file.


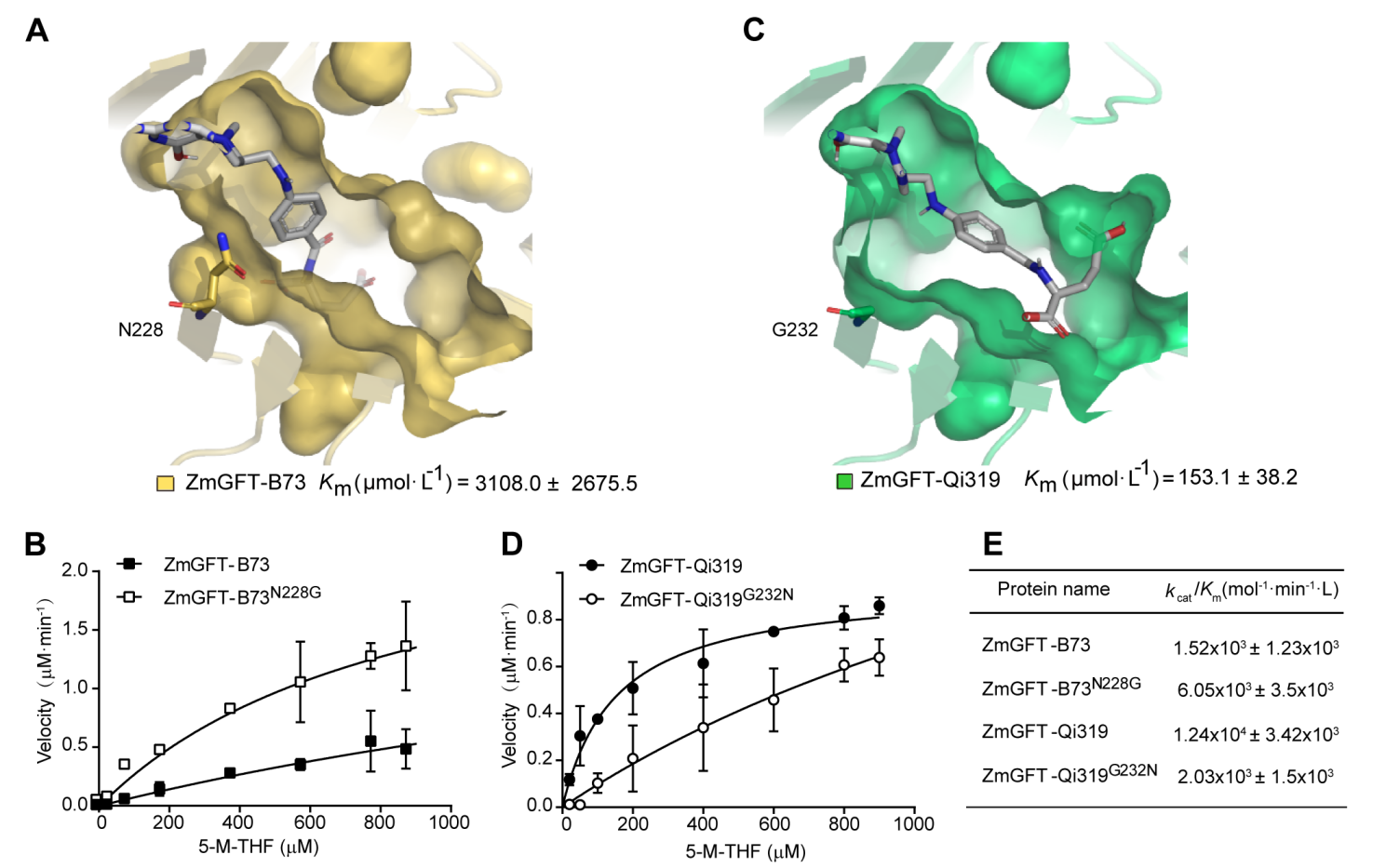


**Figure S8. The Analysis of natural variation at N228 on ZmGFT protein activity.** A) Binding pocket and binding model of 5-M-THF with ZmGFT-B73. The relative position of N228 in ZmGFT-B73 is shown. N228 is shown as a yellow stick and 5-M-THF is shown as a grey stick. All atoms are coloured according to the element (carbon, grey; oxygen, red; nitrogen, blue). N, asparagine. B) Binding pocket and binding model of 5-M-THF with ZmGFT-Qi319. The relative position of G232 in ZmGFT-Qi319 is shown. G232 is shown as a green stick and 5-M-THF is shown as a grey stick, and all atoms are colored according to the element (carbon, gray; oxygen, red; nitrogen, blue). G, glycine. C,D) The velocity of MeFox production is plotted against the concentration of 5-M-THF, which ranges from 50 μM to 900 μM. The concentration of ZmGFT was 0.5 μM. Black and white squares represent the velocity values of wild-type ZmGFT from B73 (ZmGFT-B73) and its mutant ZmGFT-B73^N228G^, respectively. The black and white circles represent the values of wild-type ZmGFT from Qi319 and ZmGFT-Qi319^G232N^, respectively. Each point represents the mean value of two independent measurements, and the error bars represent standard deviation. Fitting curves were generated using the Michaelis-Menten equation. E) *k_cat_*/*K*_m_ values of ZmGFTs and their mutants were generated by calculating *k_cat_* and *K*_m_ resulted from fitting in C,D). Source data for Figure S8B,D are provided in the Source Data file.


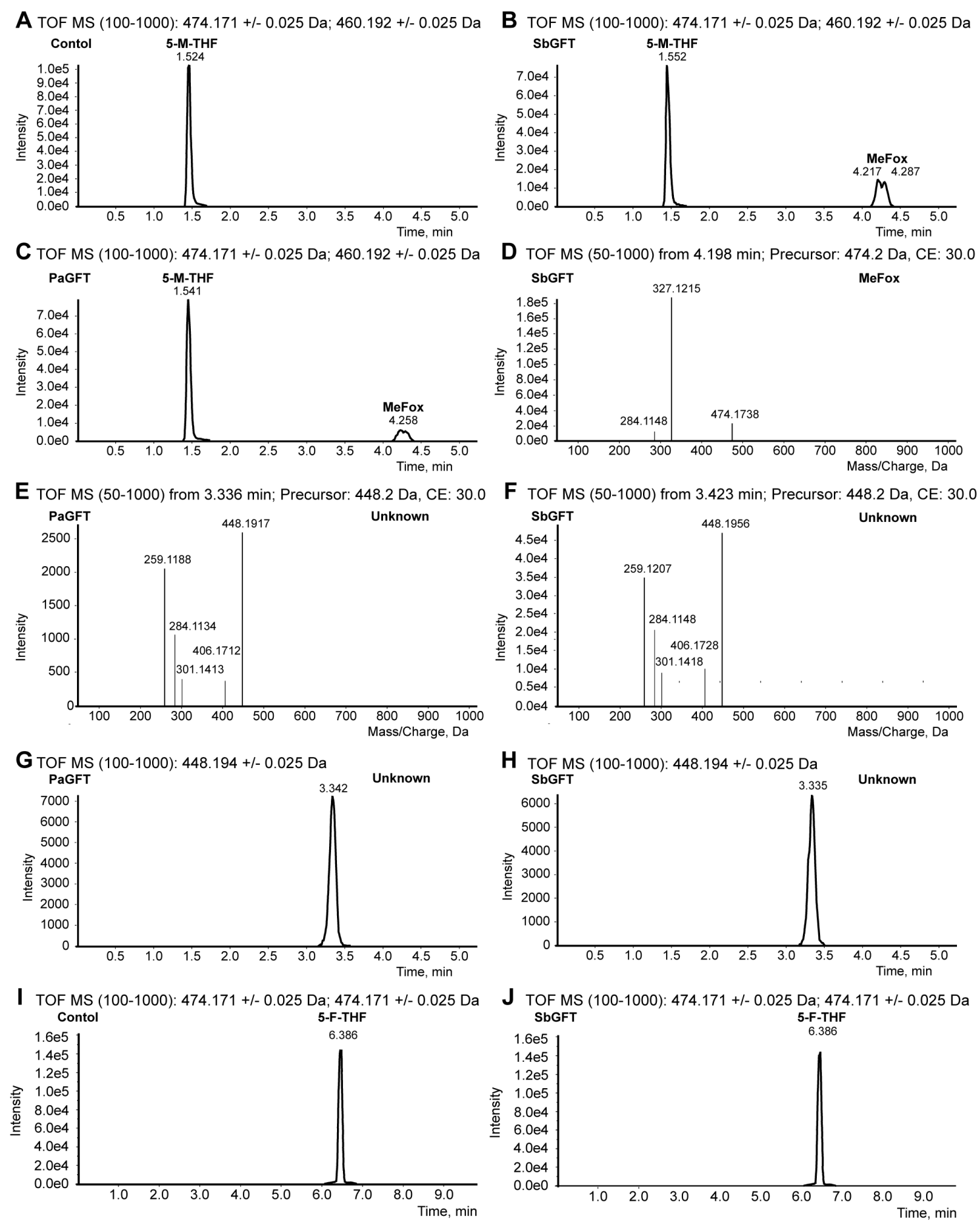


**Figure S9. UHPLC-QTOF-MS/MS analyses of *in vitro* GFT activity assay.** A) Representative mass spectra, extracted ion chromatographs (XIC) of 5-M-THF and MeFox after the incubation of 5-M-THF without GFT protein as control. B) Representative mass spectra, XIC of 5-M-THF and MeFox after the incubation of 5-M-THF with SbGFT. C) Representative mass spectra, XIC of 5-M-THF and MeFox after the incubation of 5-M-THF with PaGFT. D) High-resolution mass spectra showing the total ion chromatogram (TIC) and base peak chromatogram of MeFox after performing UHPLC-QTOF-MS/MS analyses using 5-M-THF incubated with SbGFT. E,F) High-resolution mass spectra showing the TIC and base peak chromatogram of unknown compounds after performing UHPLC-QTOF-MS/MS analyses using 5-M-THF incubated with PaGFT and SbGFT, respectively. G,H) Representative mass spectra and extract ion chromatographs of unknown compounds from the incubation of 5-M-THF with PaGFT and SbGFT, respectively. I) Representative mass spectra, XIC of 5-F-THF and MeFox after the incubation of 5-F-THF alone as control reaction. J) Representative mass spectra, XIC of 5-F-THF and MeFox after the incubation of 5-F-THF with SbGFT. No MeFox was detected. SbGFT, sequence from *Sorghum bicolor*, XP_002466878.1; PaGFT, sequence from *Prunus avium*, XP_021832372.1.


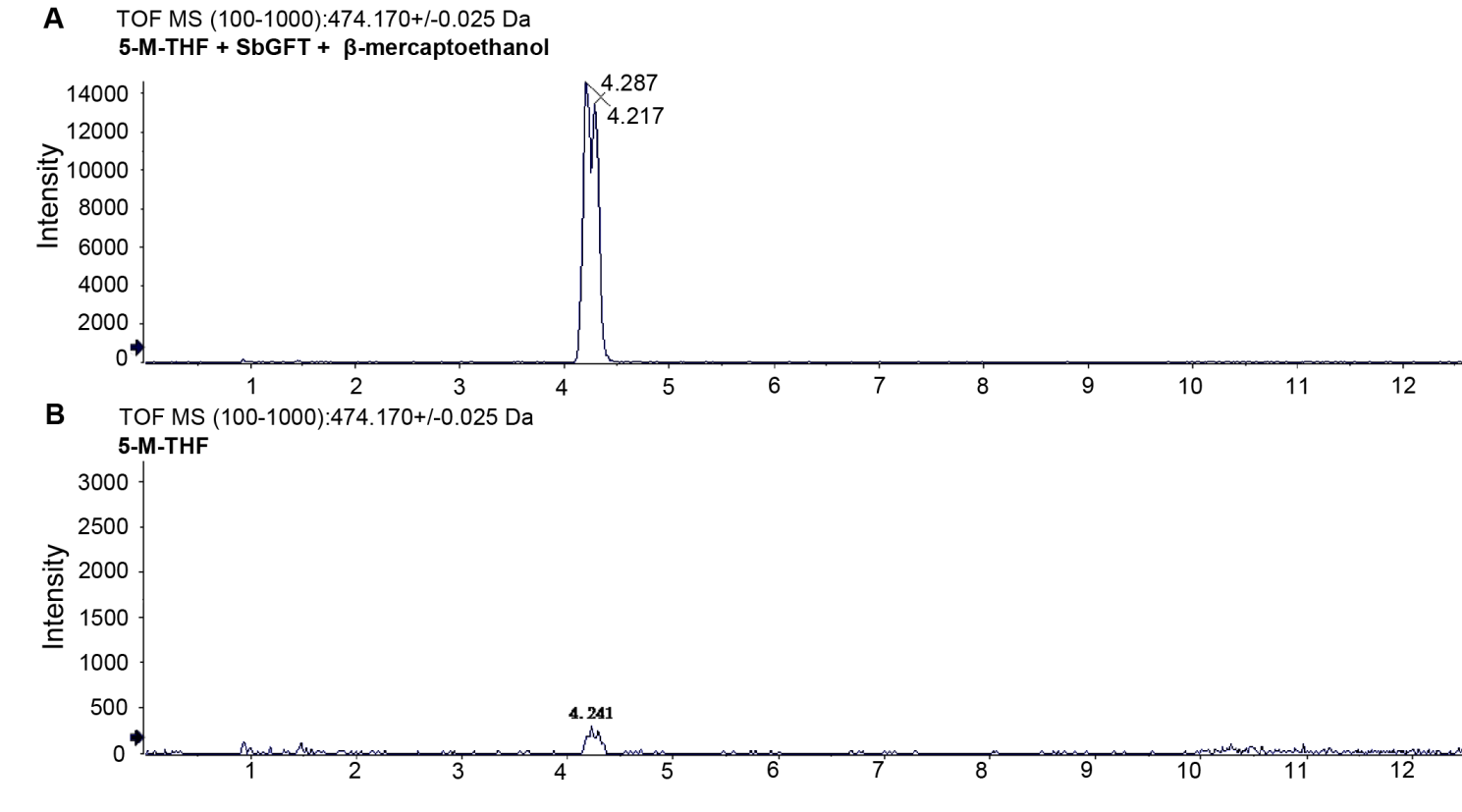


**Figure S10. Extracted Ion Chromatographs (XIC) of MeFox in the different reactions.** A) Representative mass spectra, XIC of MeFox after the incubation of 5-M-THF and SbGFT in reaction buffer with β-mercaptoethanol for two hours; B) Representative mass spectra, XIC of MeFox after the incubation of 5-M-THF alone in reaction buffer without β-mercaptoethanol for two hours. SbGFT, sequence from *Sorghum bicolor*, XP_002466878.1.


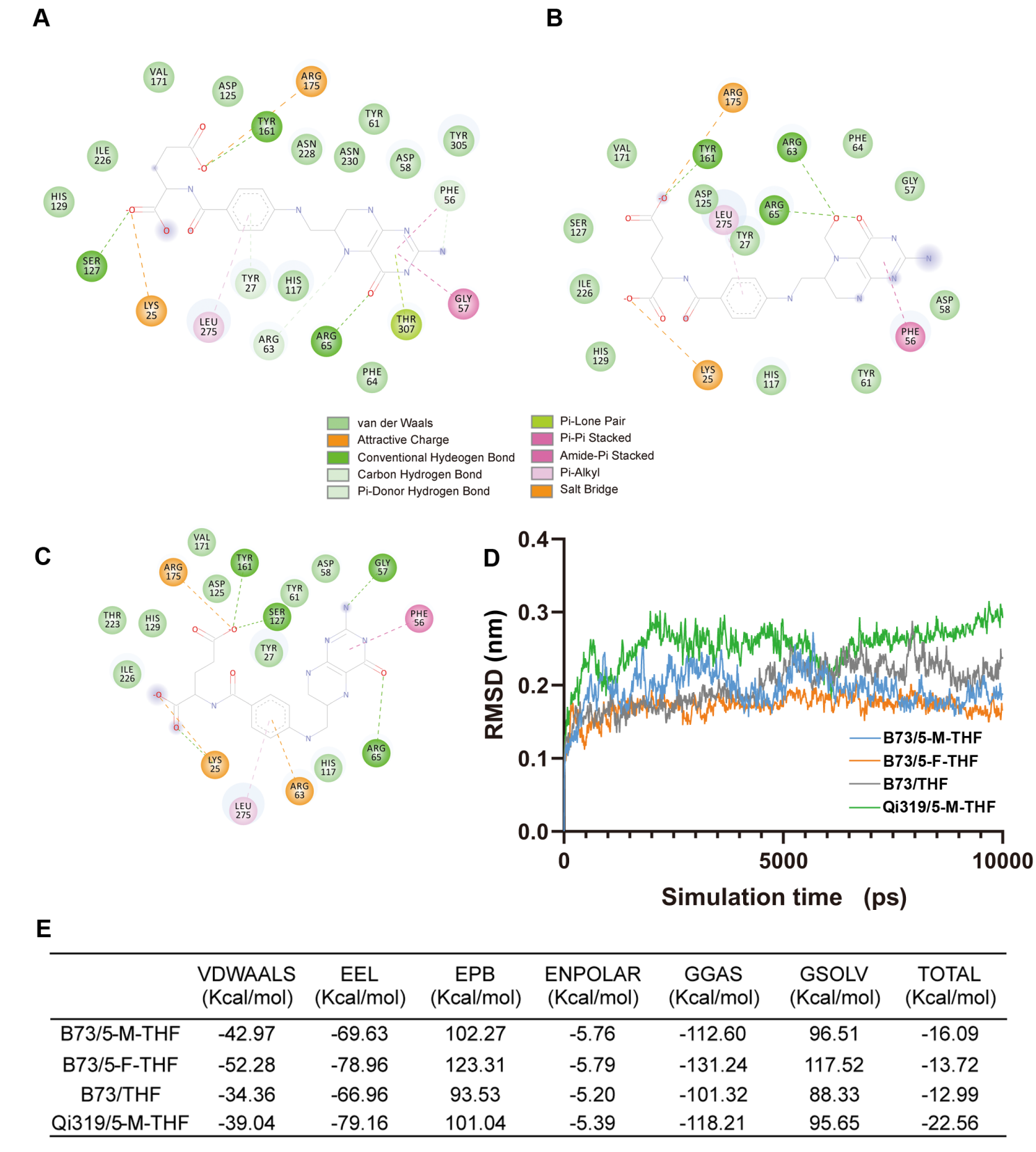


**Figure S11. Molecular simulation of ZmGFT-B73 and ZmGFT-Qi319 in complex with the different ligands.** A,B,C) 2D ligand interaction diagram of ZmGFT-B73 with 5-methyl-tetrahydrofolate (5-M-THF), 5-formyl-tetrahydrofolate (5-F-THF) and tetrahydrofolate (THF), respectively. Different color represents different interactions. D) RMSDs of the MD simulation between B73 and 5-M-THF (the blue line), B73 and 5-F-THF (the orange line), B73 and THF (the grey line), Qi319 and 5-M-THF (the green line) complex systems, respectively. E) Van der Waals energy (VDWAALS), Electrostatic energy (EEL), Polar solvation energy (EPB), Non-polar solvation energy (ENPOLAR), General gas phase energy (GGAS), General solvation energy (GSOLV) and Total binding free energy of the selected ligands against ZmGFT-B73 and ZmGFT-Qi319.


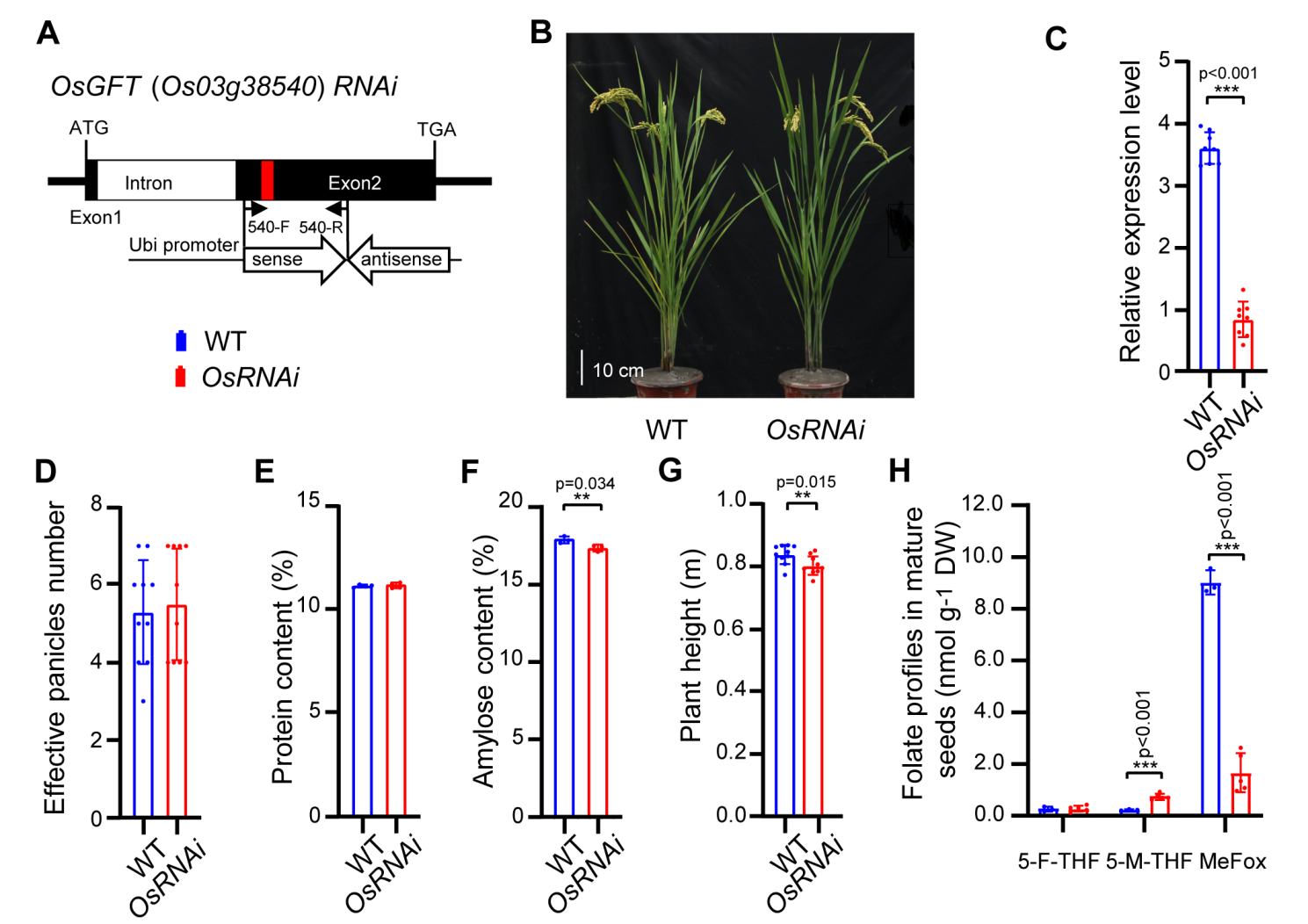


**Figure S12. Phenotypic analyses of transgenic *OsGFT-*RNAi rice.** A) Gene structure of *OsGFT* and RNAi primers in exon 2. B) Photos of WT and *OsRNAi* plants at day after sowing 100. C) Relative expression levels of *ZmGFT* in leaves of the wild-type (WT) rice and *OsGFT-*RNAi (*OsRNAi*) plants as mean ± SD of eight biological replicates. D) Effective panicles of WT and *OsRNAi* rice*.* E) Protein contents in mature seeds of WT and *OsRNAi* rice*.* F) Amylose contents in mature seeds of WT and *OsRNAi* rice*.* G) Plant heights of WT and *OsRNAi* rice*.* Yandao8. H) Folate profiles in mature seeds of wild type (WT) and *OsGFT-*RNAi (*OsRNAi*) rice plants. Rice line Yandao 8, the genetic transformation recipient, was used as the WT. Accumulated folate derivatives are presented as mean ± SD of three for WT and five for *OsRNAi* biological replicates, respectively. Mean values of ten biological replicates are shown for effective panicles plant height, three and four replicates for amylose and protein contents, respectively. *p*-values are shown (Student’s *t*-test, *, *p* < 0.05; **, *p* < 0.01; ***, *p* < 0.001). Source data for Figure S12C,D,E,F,G,H are provided in the Source Data file.


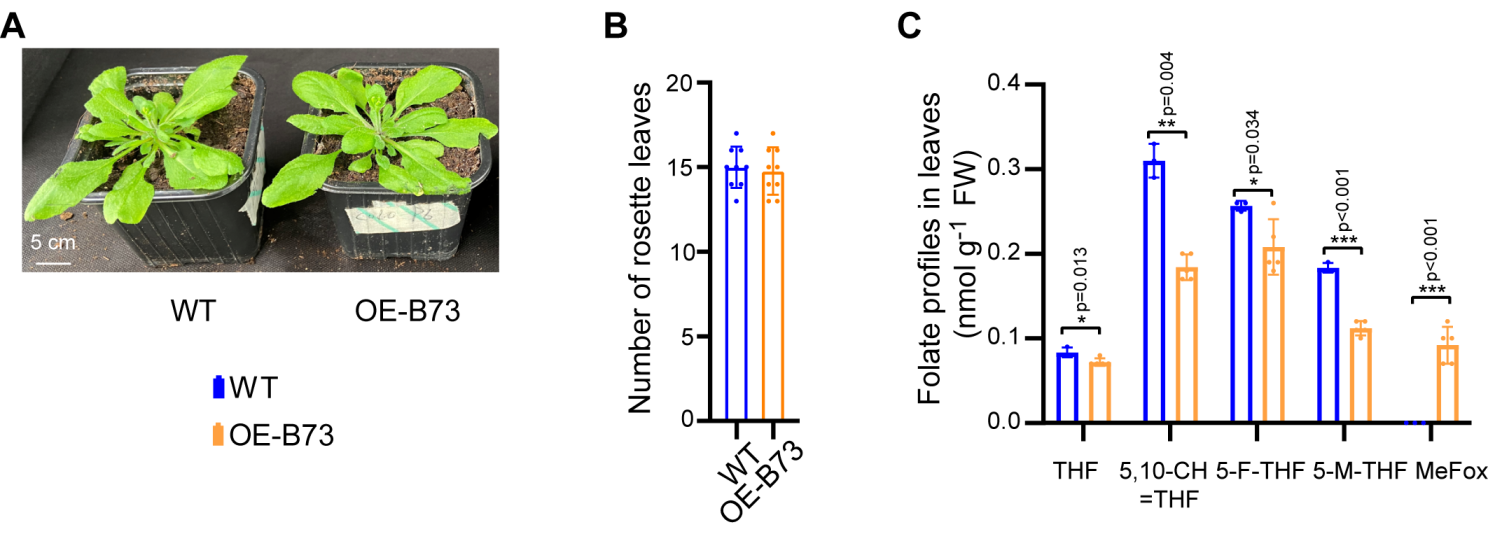


**Figure S13. Phenotypic analyses of transgenic *ZmGFT*-overexpressing Arabidopsis plants. A**) Photos of 30-day rosette leaves of *ZmGFT-B73* transgenic (OE-B73; right) and wild-type (WT; left) Arabidopsis plants. B) Number of rosette leaves of 30-day-old Arabidopsis plants. Data are presented as mean ± SD of nine biological replicates *Arabidopsis thaliana* ecotype Columbia was used as the wild-type Arabidopsis. C) Folate profiles in 30-day-old rosette leaves of wild type (WT; Columbia) or *ZmGFT-B73-*overexpressing *Arabidopsis* plants (OE-B73). Accumulated folate derivatives are presented as mean ± SD of three for WT and five for OE-B73 biological replicates, respectively. *p*-values are shown (Student’s *t*-test, *, *p* < 0.05; **, *p* < 0.01; ***, *p* < 0.001). Error bars represent standard deviations. Source data for Figure S13C are provided in the Source Data file.


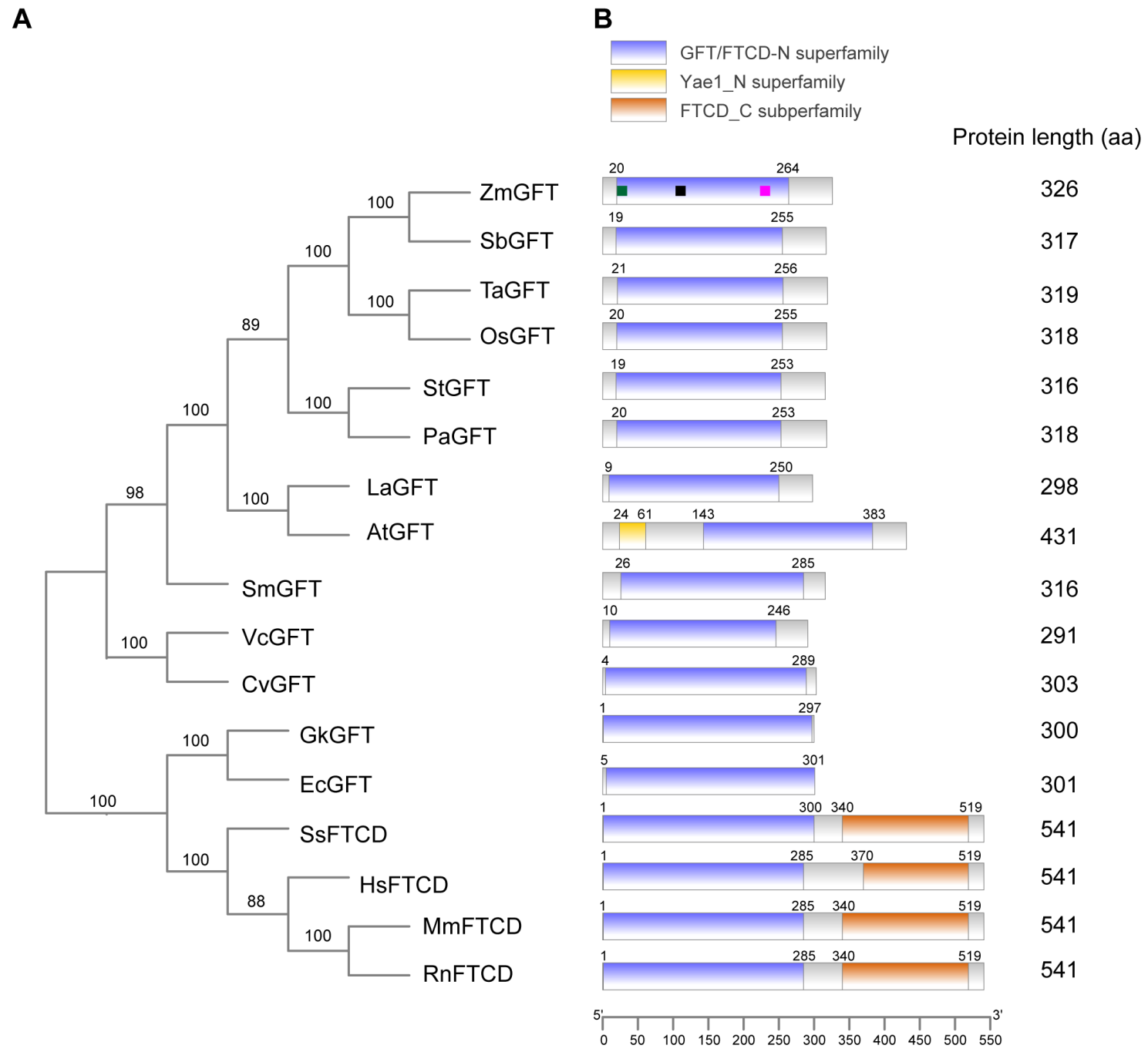


**Figure S14.** **Phylogenetic tree and conserved domain analysis of GFT orthologs from different organisms.** A) Phylogenetic tree of GFT orthologues from different organisms. B) Conserved domain analysis of GFT orthologues from different organisms. On the protein of ZmGFT, three cubes labeled different sites in the results. Dark green cubes represent the changed serine (S) at position 20 where the peptide started to change due to the gene-editing in *ZmGFT*-editing protein. Black solid cube represents histidine (H) at position 117 in ZmGFT-B73 that was mutated for the activity assay. Pink solid cube represents asparagine (N) at position 228 in ZmGFT-B73. ZmGFT-B73, sequence from maize (*Zea mays*) inbred line B73, NP_001130076.1; ZmGFT-Qi319, sequence from maize inbred line Qi319, AMK92167.1; SbGFT, sequence from *Sorghum bicolor*, XP_002466878.1; TaGFT, sequence from *Triticum aestivum*, KAF6990789.1; OsGFT, sequence from *Oryza sativa*, XP_015633257.1; PaGFT, sequence from *Prunus avium*, XP_021832372.1; StGFT, sequence from *Solanum tuberosum*, XP_006357514.1; LaGFT, sequence from *Lunaria annua*, Luann.0189s0003.1; AtGFT, sequence from *Arabidopsis thaliana*, NP_973497.1; CvGFT, sequence from *Crucigenia variabilis*, XP_005850624.1; VcGFT, sequence from *Volvox carteri*, [XM_002946226.1](https://www.ncbi.nlm.nih.gov/nuccore/XM_002946226.1); SmGFT sequence from *Selaginella moellendorffii*, XP_002964559.1; GkGFT, sequence from *Gloeobacter kilaueensis*, WP_023175819.1; EcGFT, sequence from *Escherichia coli*, MZZ90505.1; SsFTCD, sequence from *Sus scrofa*, NP_999440.1; HsFTCD, sequence from *Homo sapiens*, NP_006648.1; MnFTCD, sequence from *Mus musculus*, NP_543121.1; RnFTCD, sequence from *Rattus norvegicus*, NP_446019.1.


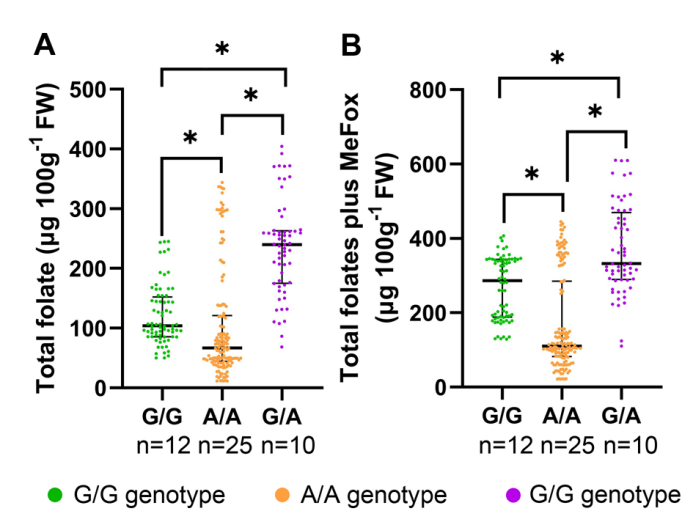


**Figure S15. Total folate of commercial hybrid sweetcorn.** A) Total folates of young seeds from sweetcorn with homozygous G/G-allele, homozygous A/A-allele, and heterozygous G/A. B) Total folates plus MeFox of young seeds from sweetcorn with homozygous G/G-allele, homozygous A/A-allele, and heterozygous G/A. Dot plots display the distribution of total folate content (μg 100g⁻¹ FW) across different genotypes. Each dot represents an individual raw data point. The solid horizontal line indicates the median, and the thin horizontal lines indicate the interquartile range (IQR). Statistical significance was assessed using the two-sided Mann-Whitney U test. Asterisks indicate significant differences (*, p < 0.01). The data are presented as median and IQR from six biological replicates, respectively. Source data for Figure S15A,B are provided in Table S14 and the Source Data file.


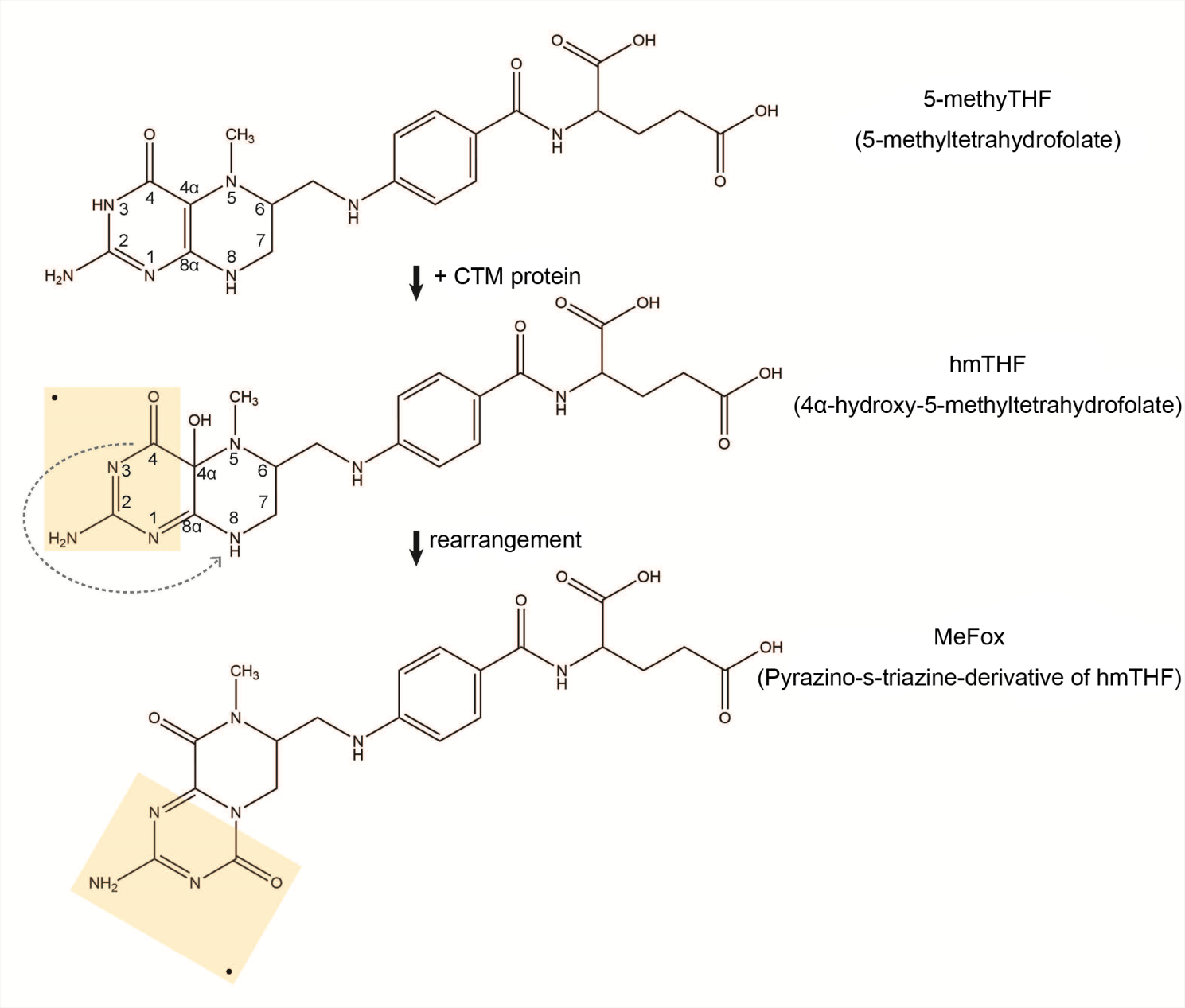


**Figure S16. Proposed mechanism for the GFTs facilitating oxidation of 5-M-THF to MeFox.** GFTs from plants trigger the conversion of 5-M-lTHF to 4α-hydroxy-5methylTHF (hmTHF) by the possible insertion of hydroxy group at 4α position (upper panel to middle panel). Subsequently, rearrangement of pteridine nucleus (C-4α hydroxy group being converted to a carbonyl group in the oxidation process) occurs with the formation of a pyrazino-s-triazine derivative (MeFox).


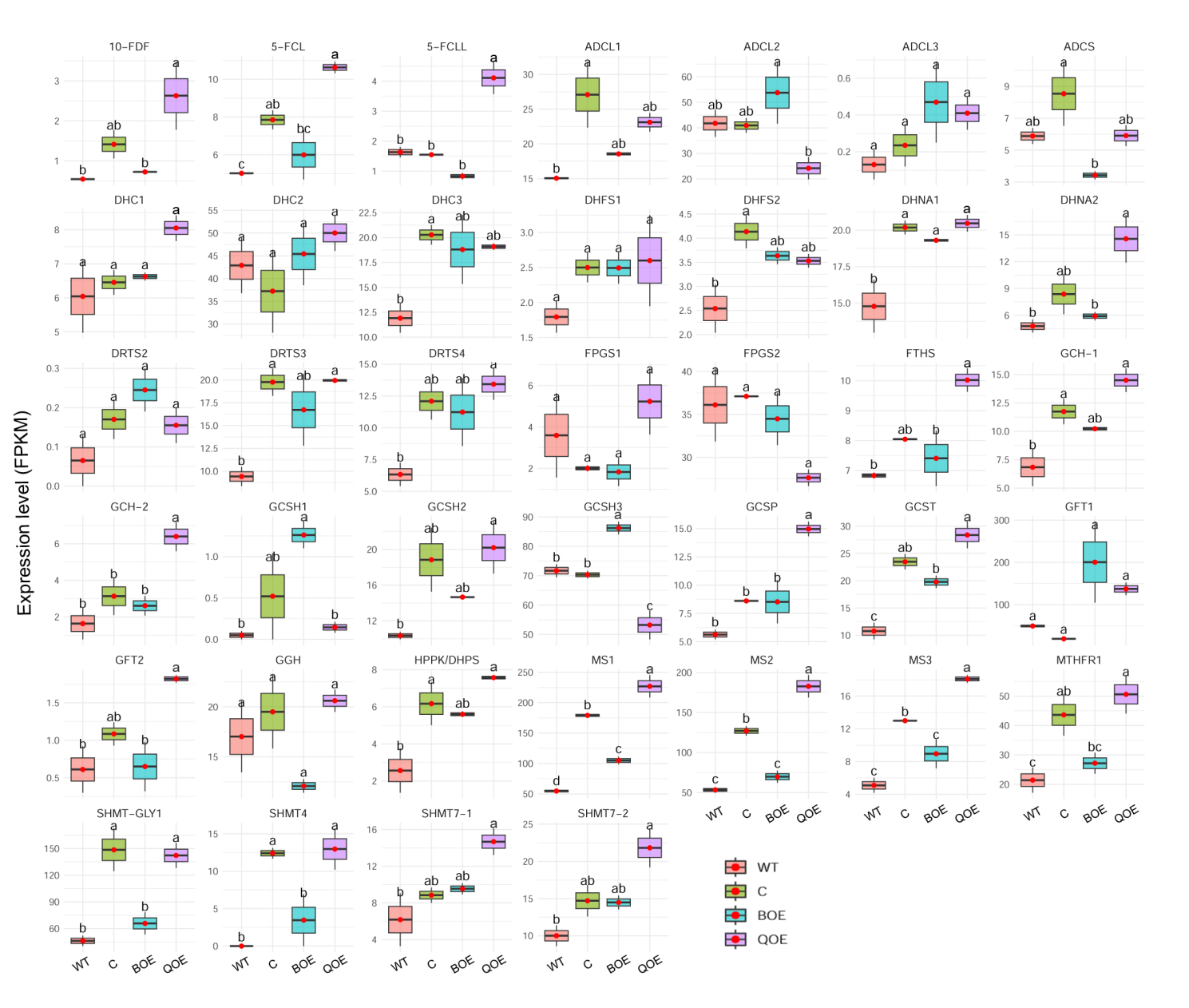


**Figure S17. RNA-Seq profile of folate-related genes from wild type, Crispr, OE-B73 and OE-Qi319 lines.** Expression levels of folate-related genes in young seeds (DAP 25) from wild type (WT, W), Crispr (C), overexpressor of ZmGFT-B73 (OE-B73, BOE) and overexpressor of ZmGFT-Qi319 (OE-Qi319, QOE) lines as mean ± SD of two biological replicates. Error bars represent standard deviations. 5-FCL, 5-F-THF cycloligase; 5-FCL, 5-F-THF cycloligase-like; 10-FDF, 10-formyl THF deformylase; ADCL, 4-aminodeoxychorismate lyase; ADCS, 4-aminodeoxychorismate synthase; DHC, 5,10-methylene-THF dehydrogenase/5,10-methenyl-THF cyclohydrolase; DHFS, dihydrofolate synthase; DHNA, 7,8-dihydroneopterin aldolase; DRTS, dihydrofolate reductase-thymidylate synthase; FPGS, folylpolyglutamate synthetase; FTHS, 10-formyltetrahydrofolate synthetase; GCH, GTP cyclohydrolase I; GCS (GDC), glycine cleavage system (glycine decarboxylase complex), including H-protein, P-protein and T-protein; GFT, glutamate formiminotransferase; GGH, γ-glutamyl-hydrolase; HPPK/DHPS, hydroxymethyldihydropterin pyrophosphokinase and dihydropteroate synthase; MS, methionine synthase; MTHFR, methylenetetrahydrofolate reductase; SHMT, serine hydroxymethyltransferase. Source data for Figure S17 are provided in Table S15.


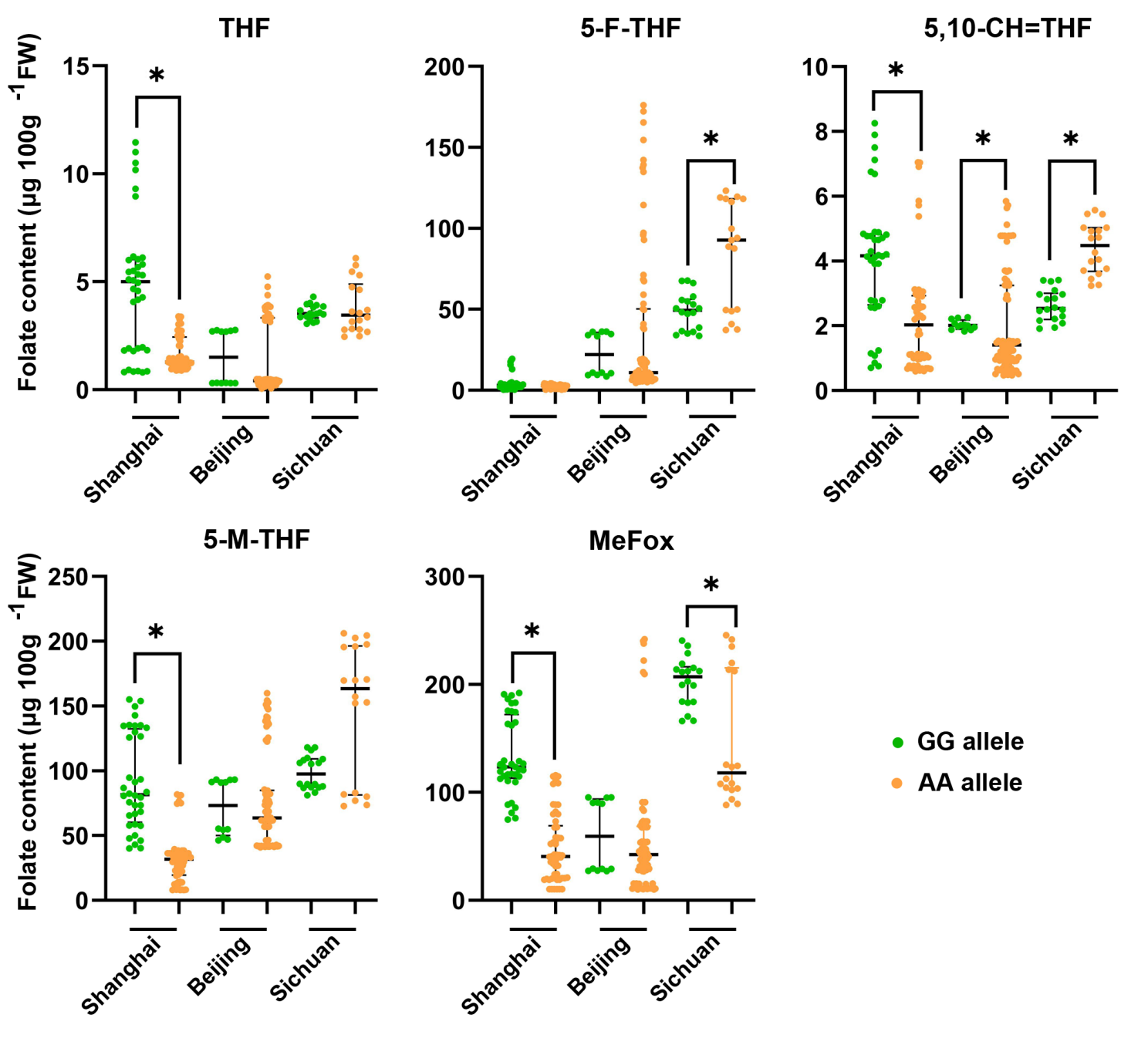


**Figure S18. Folate profiles of commercial hybrid sweetcorn from different source.** The commercial hybrid sweetcorn were collected from different sources and then planted in the same location (Langfang, Hebei), the young seeds were collected at 20-24 DAP (harvest stage) for folate detection. Shanghai, the sweetcorn from Shanghai with homozygous G/G-allele and A/A-allele. Beijing, the sweetcorn from Beijing with homozygous G/G-allele and A/A-allele. Sichuan, the sweetcorn from Sichuan with homozygous G/G-allele and homozygous A/A-allele. Each dot represent an individual raw data point from six biological replicates.. The solid horizontal line indicates the median, and the thin horizontal lines indicate the interquartile range (IQR). Statistical significance was assessed using the two-sided Mann-Whitney U test. Asterisks indicate significant differences (*, p < 0.01). Samples from Guangdong are not included for the analysis since no A/A and only one G/G were detected. Source data for Figure S18 are provided in Table S14 and the Source Data file.

**Supplemental Table S1**. Folate profile of 09ZHN (n = 501).

**Supplemental Table S2.** Folate profile of 10WY (n = 406).

**Supplemental Table S3.** Folate profile of 10AMH (n = 460).

**Supplemental Table S4.** Folate profile of best linear unbiased prediction data for the GWAS (n = 531).

**Supplemental Table S5.** Phenotypic variation and heritability analysis in the association panel (n = 531).

**Supplemental Table S6.** Genes associated with the content of 5-M-THF identified in the genome-wide association analysis.

**Supplemental Table S7.** Folate profiles in mature seeds of maize inbred lines grown in Hebei.

**Supplemental Table S8.** Association of MeFox with the alleles of locus SNP1789 in ZmGFT based on re-sequenced inbred lines.

**Supplemental Table S9.** Amino acid profiles in mature and young seeds of KN5585, ZmGFT-editing maize (Crispr), ZmGFT-Qi319 overexpressors (OE-Qi319), and ZmGFT-B73 overexpressors (OE-B73).

**Supplemental Table S10.** *kcat*, *Km*, and *kcat/Km* of GFT proteins.

**Supplemental Table S11.** The situation of the detected known compounds when folate derivative was incubated with SbGFT, respectively.

**Supplemental Table S12.** Data collection and refinement statistics of SbGFT.

**Supplemental Table S13.** Virtual screen against ZmGFT-B73 protein.

**Supplemental Table S14.** Folate profile of commercial sweetcorn hybrids grown in Hebei.

**Supplemental Table S15.** Transcriptomic data (FPKM value) of folate-related genes from wild type, Crispr, BOE and QOE lines.

**Supplemental Table S16.** Docking data of ZmGFT-Qi319 and ZmGFT-B73 with 5-M-THF.

**Supplemental Table S17.** Primers used in this study.
