# Supplementary figures and images for: An Allele of Glutamate Formiminotransferase Triggers 5‐Methyl‐Tetrahydrofolate‐to‐MeFox Conversion and Facilitates Folate Biofortification in Maize
Source: Adv Sci (Weinh). 2025 Aug 21;12(42):e15082. doi: 10.1002/advs.202415082 (PMC12622545; doi:10.1002/advs.202415082)

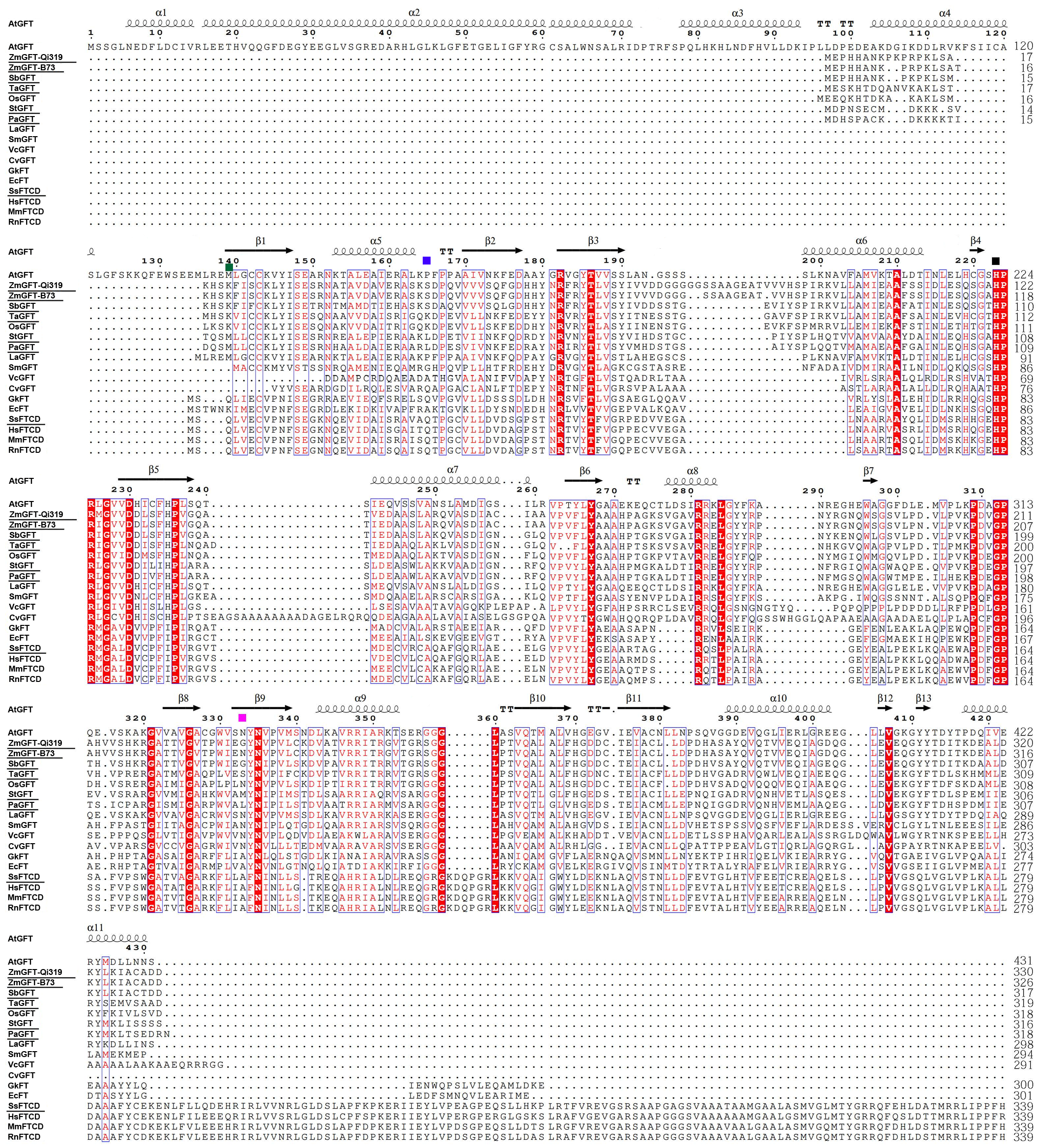

Supplement: Supplementary file 2 — Supplemental Figure 1 [file ADVS-12-e15082-s002.tif]

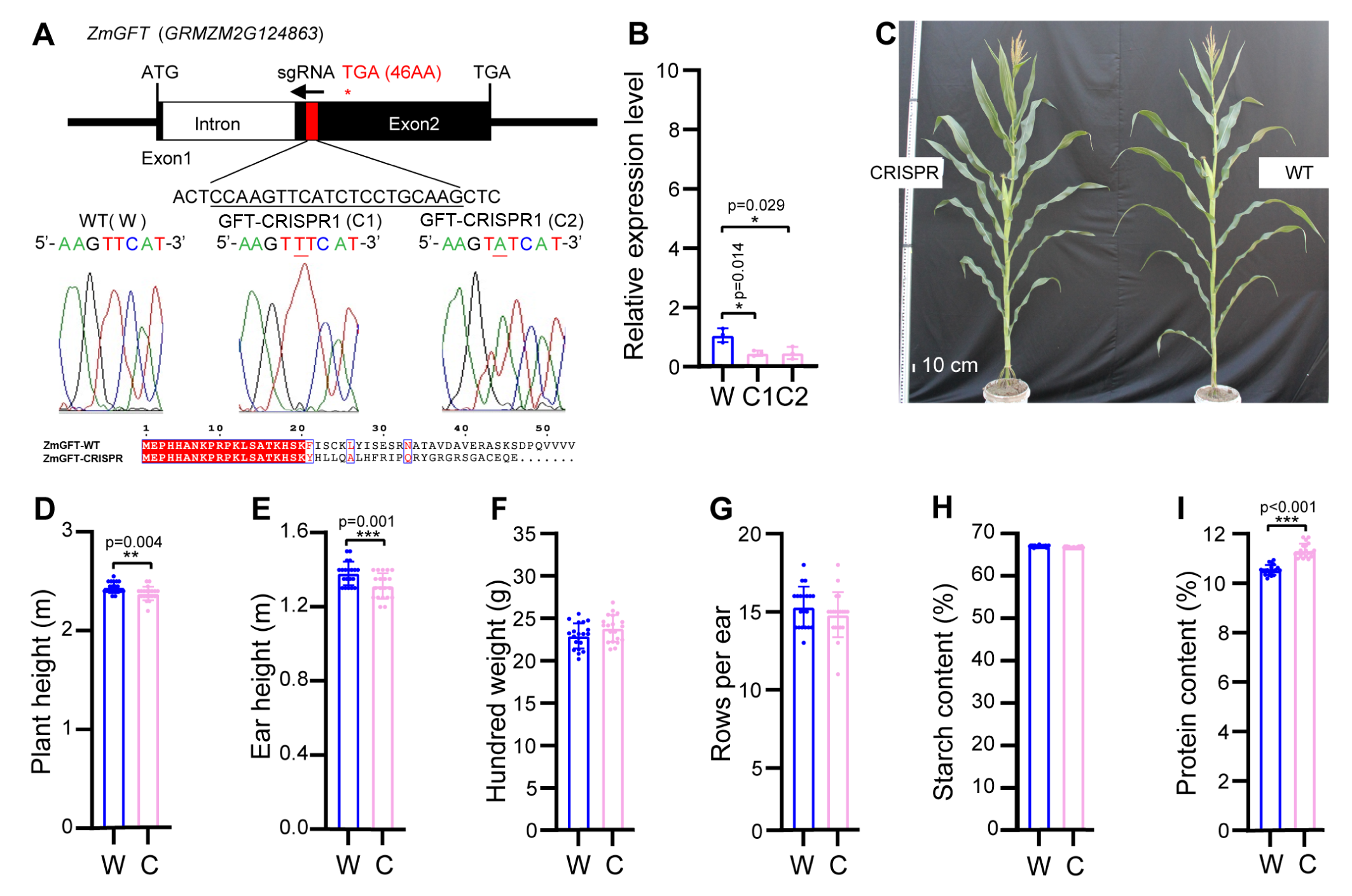

Supplement: Supplementary file 3 — Supplemental Figure 2 [file ADVS-12-e15082-s034.tif]

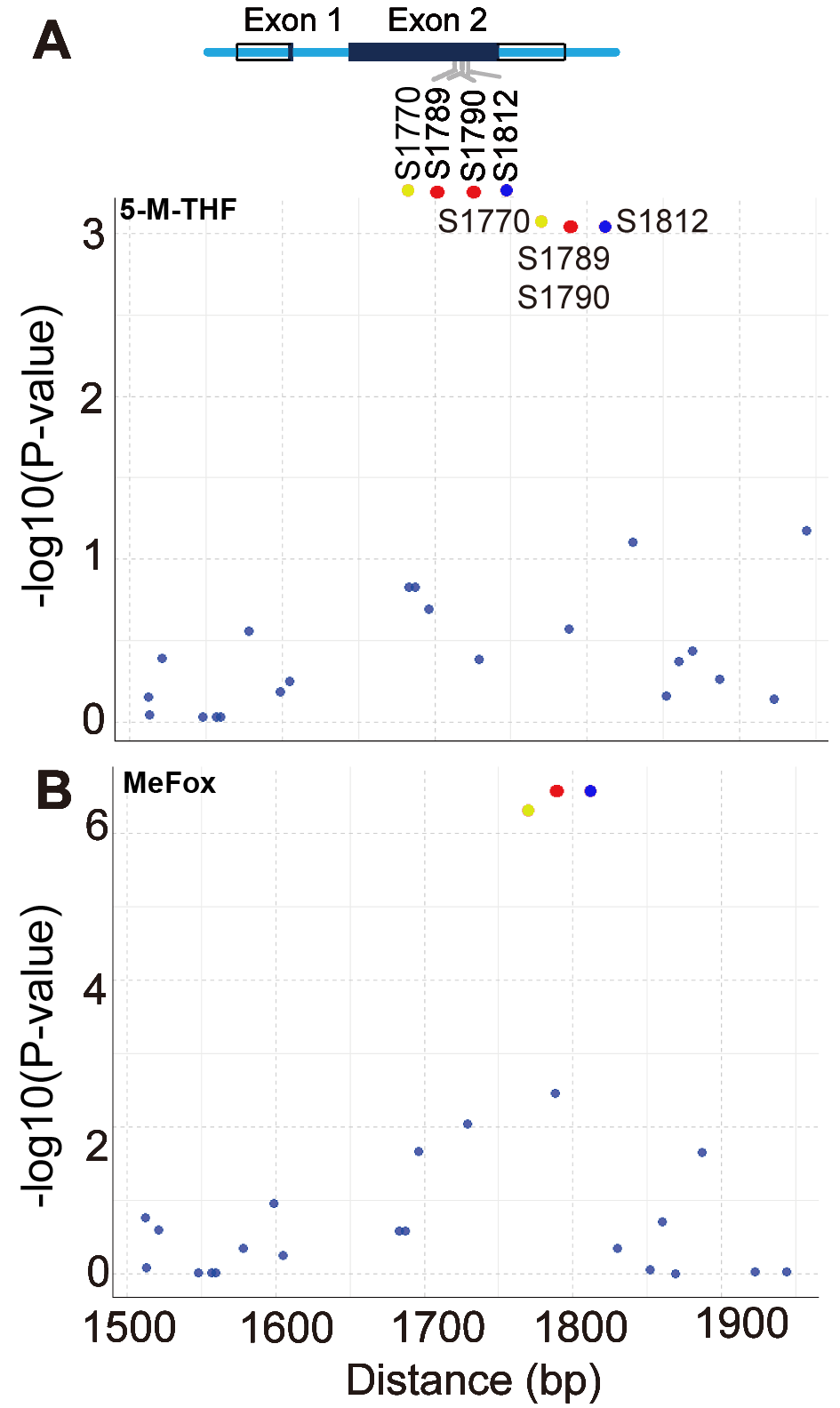

Supplement: Supplementary file 4 — Supplemental Figure 3 [file ADVS-12-e15082-s025.tif]

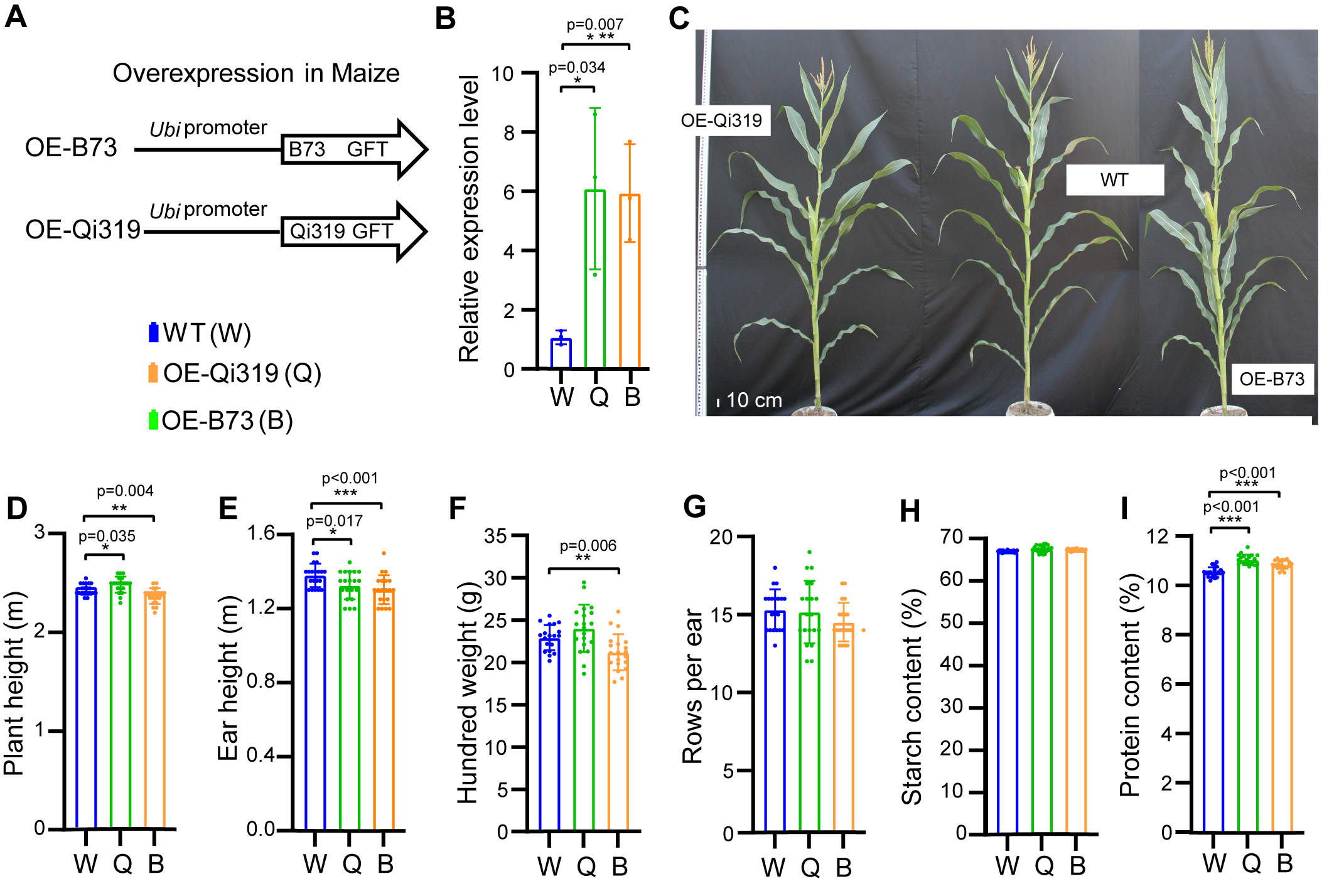

Supplement: Supplementary file 5 — Supplemental Figure 4 [file ADVS-12-e15082-s029.tif]

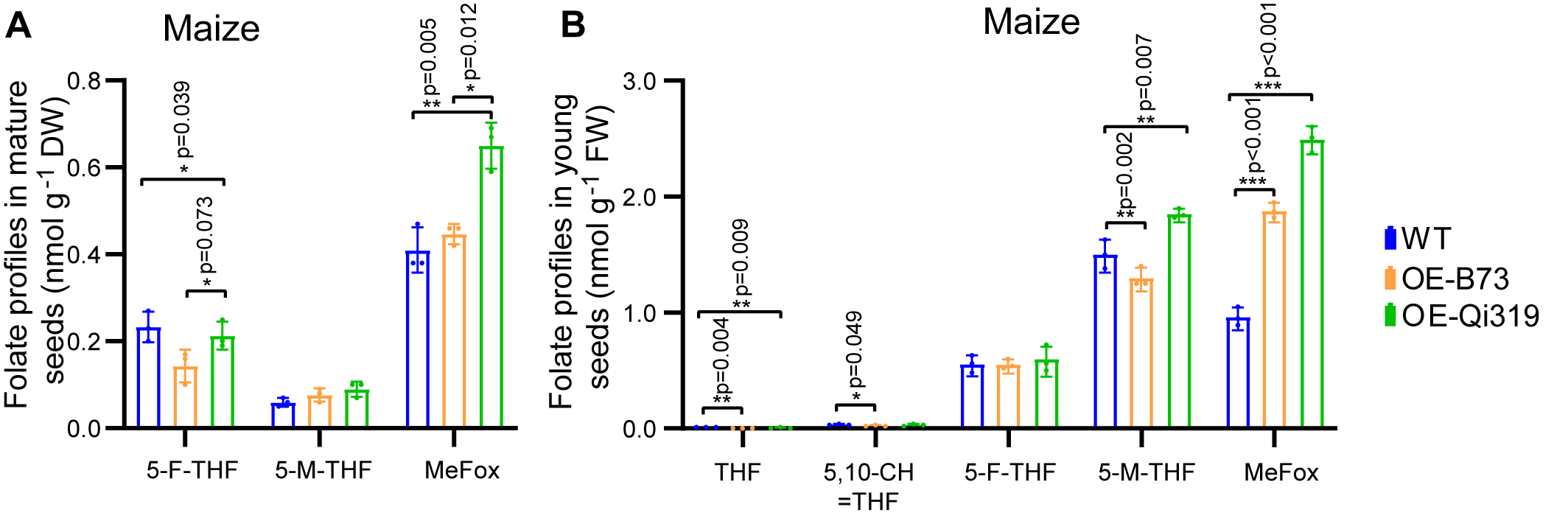

Supplement: Supplementary file 6 — Supplemental Figure 5 [file ADVS-12-e15082-s036.tif]

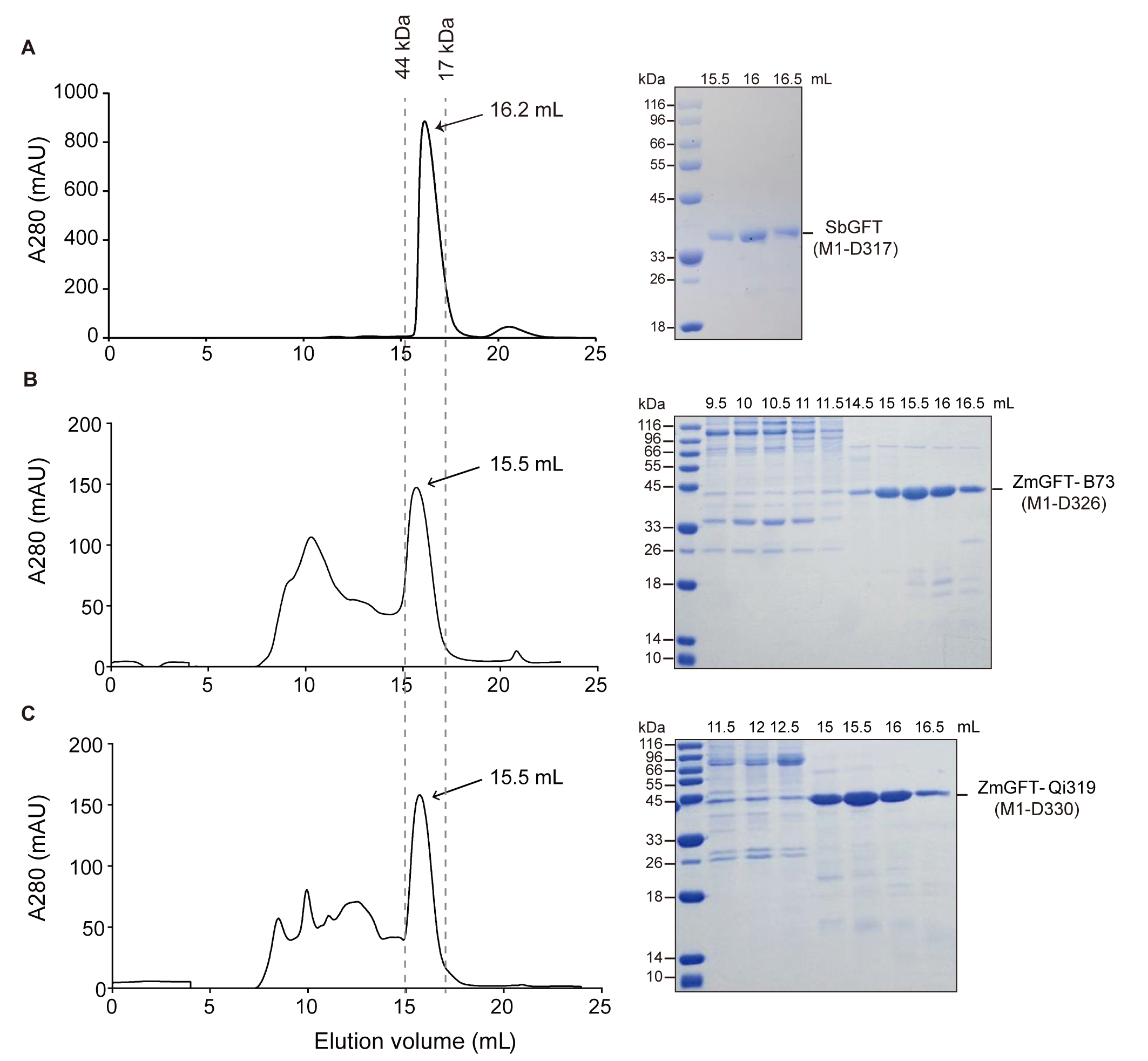

Supplement: Supplementary file 7 — Supplemental Figure 6 [file ADVS-12-e15082-s009.tif]

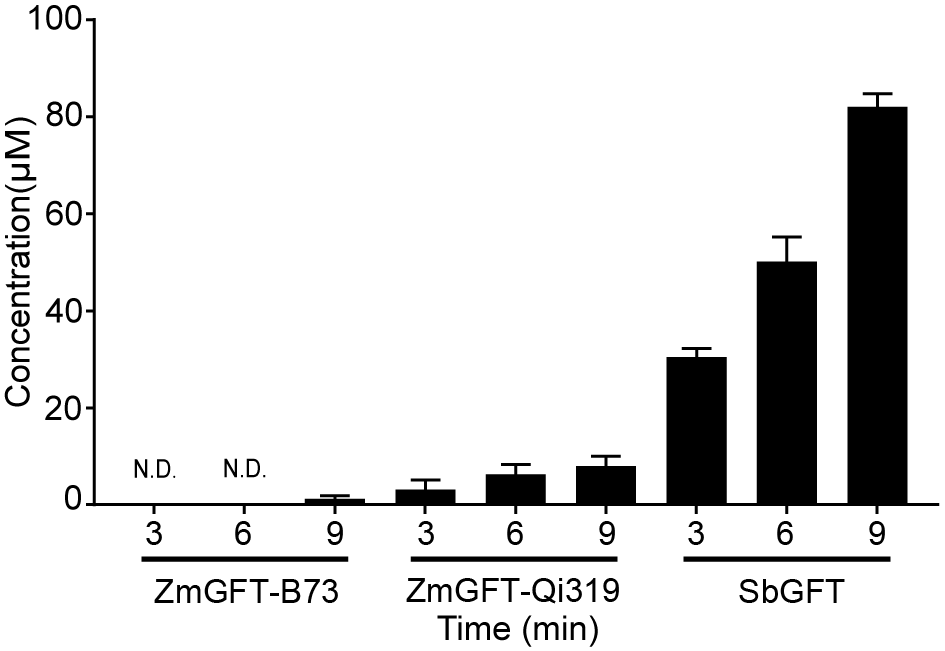

Supplement: Supplementary file 8 — Supplemental Figure 7 [file ADVS-12-e15082-s016.tif]

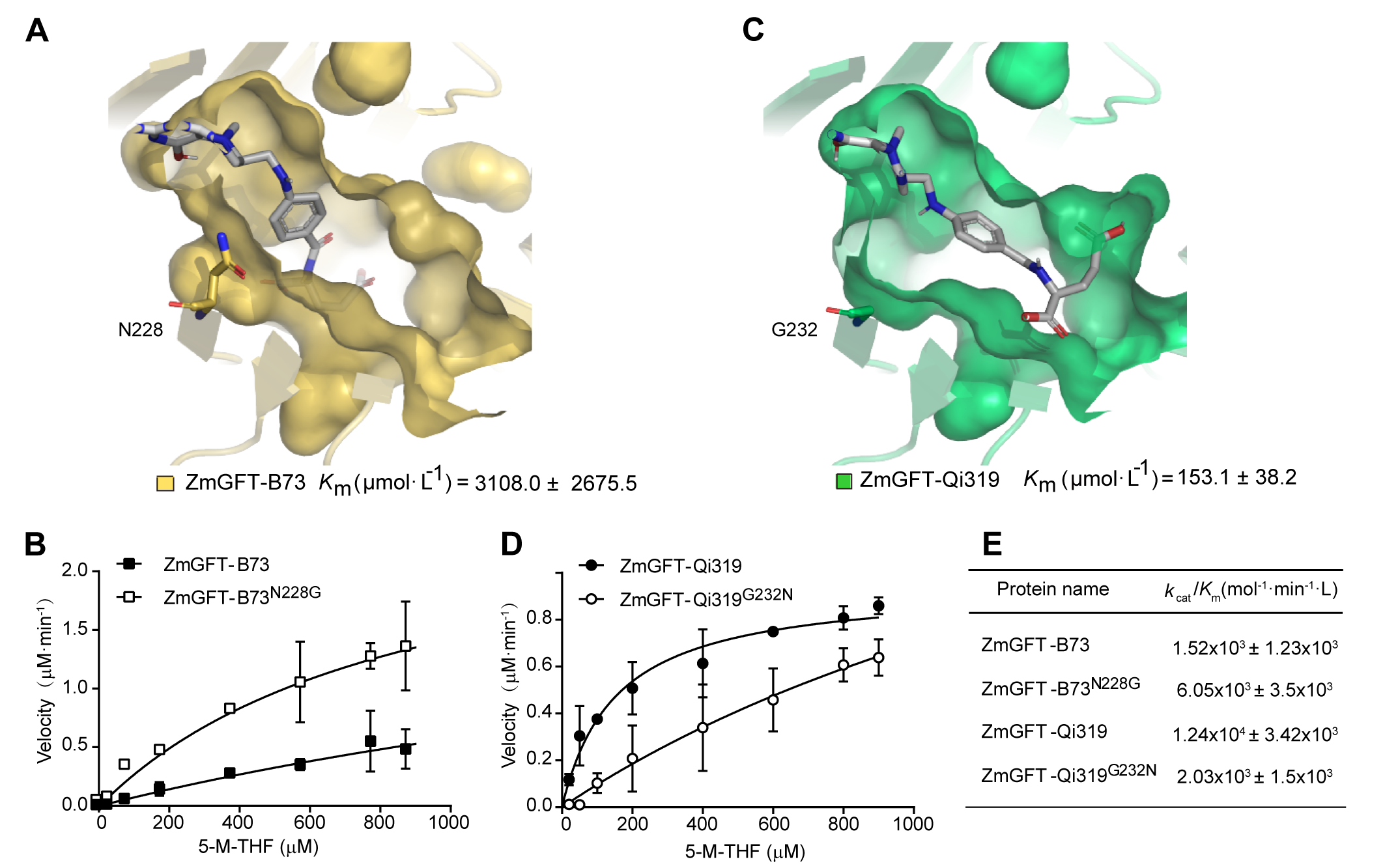

Supplement: Supplementary file 9 — Supplemental Figure 8 [file ADVS-12-e15082-s014.tif]

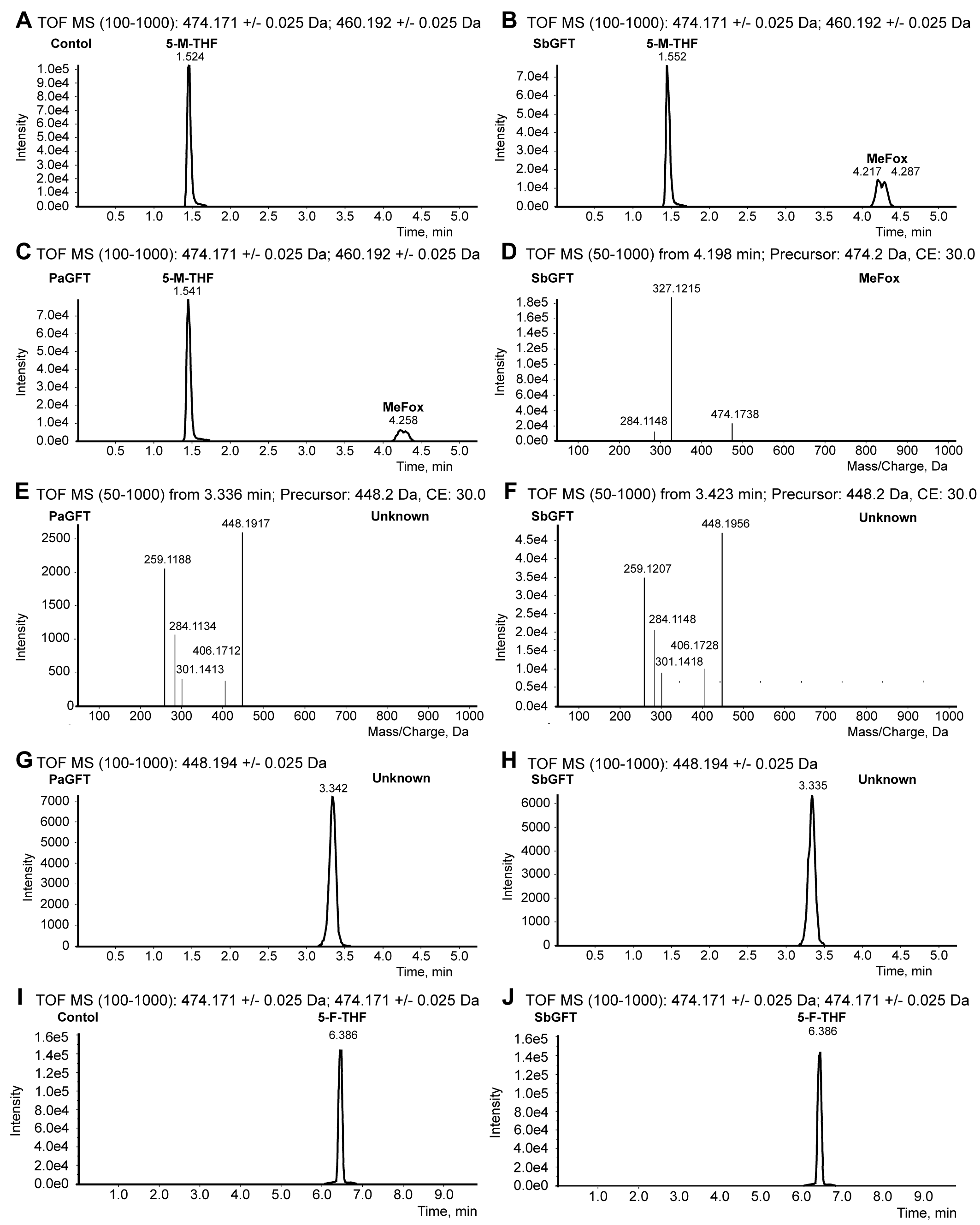

Supplement: Supplementary file 10 — Supplemental Figure 9 [file ADVS-12-e15082-s018.tif]

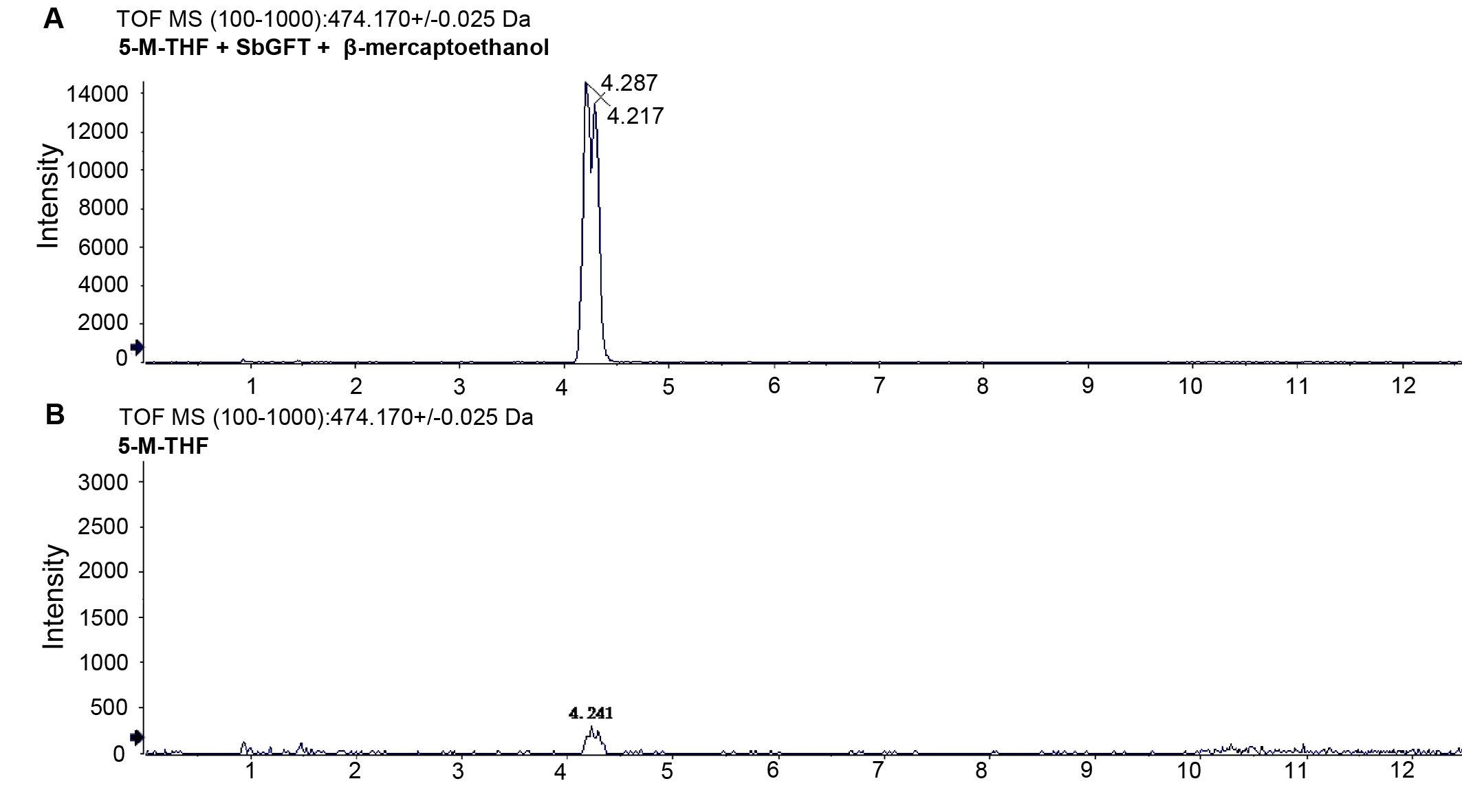

Supplement: Supplementary file 11 — Supplemental Figure 10 [file ADVS-12-e15082-s020.tif]

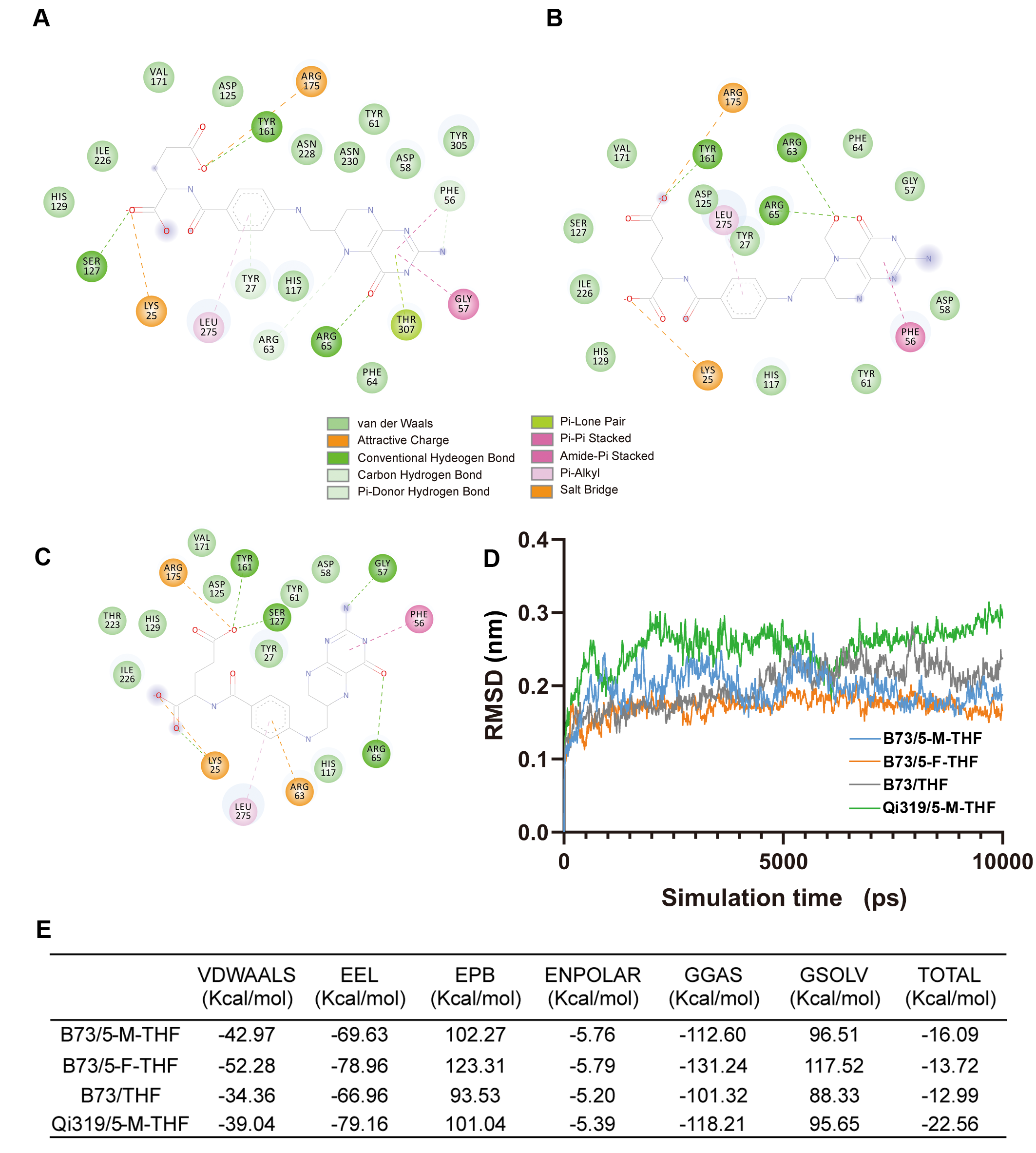

Supplement: Supplementary file 12 — Supplemental Figure 11 [file ADVS-12-e15082-s013.tif]

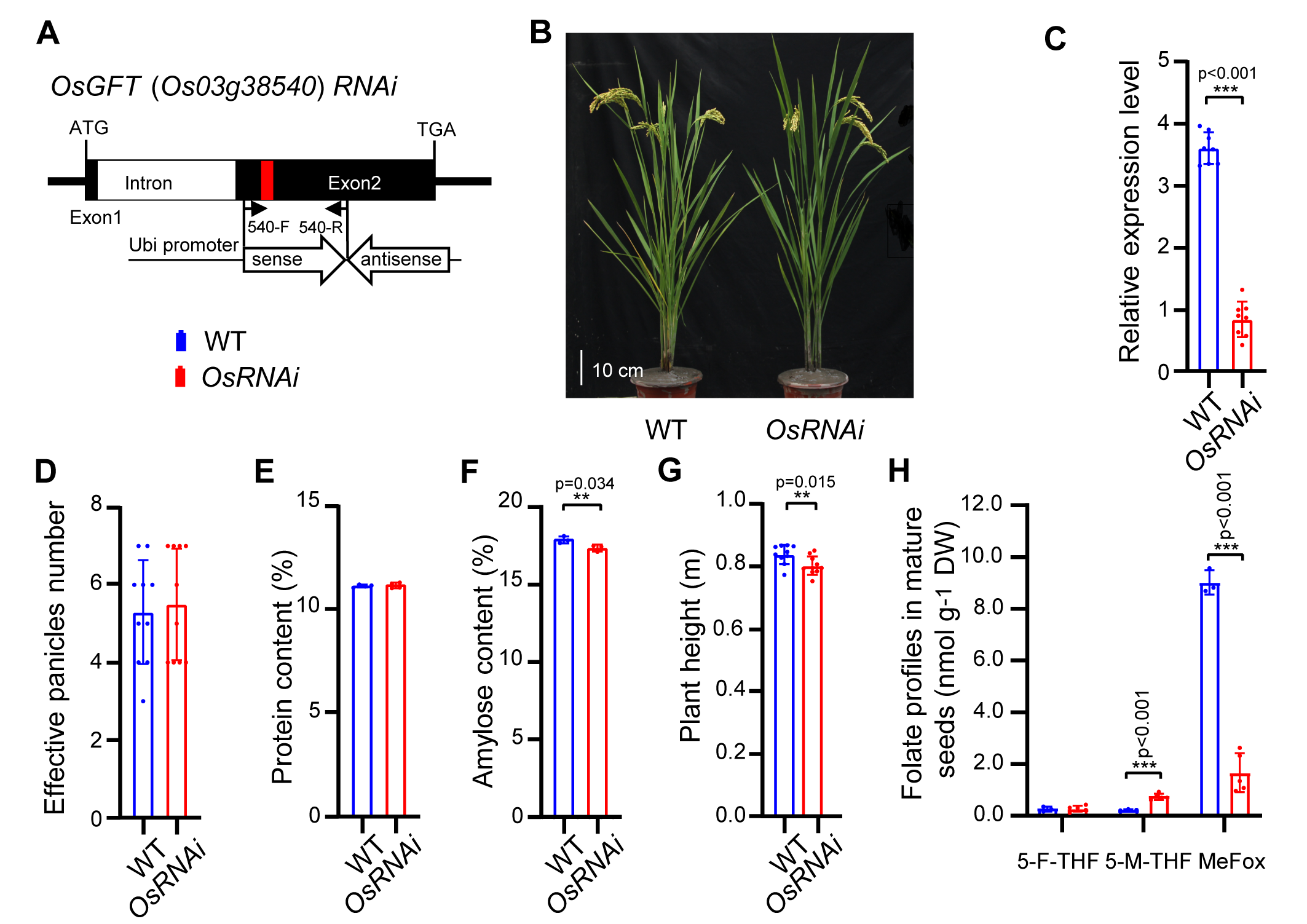

Supplement: Supplementary file 13 — Supplemental Figure 12 [file ADVS-12-e15082-s031.tif]

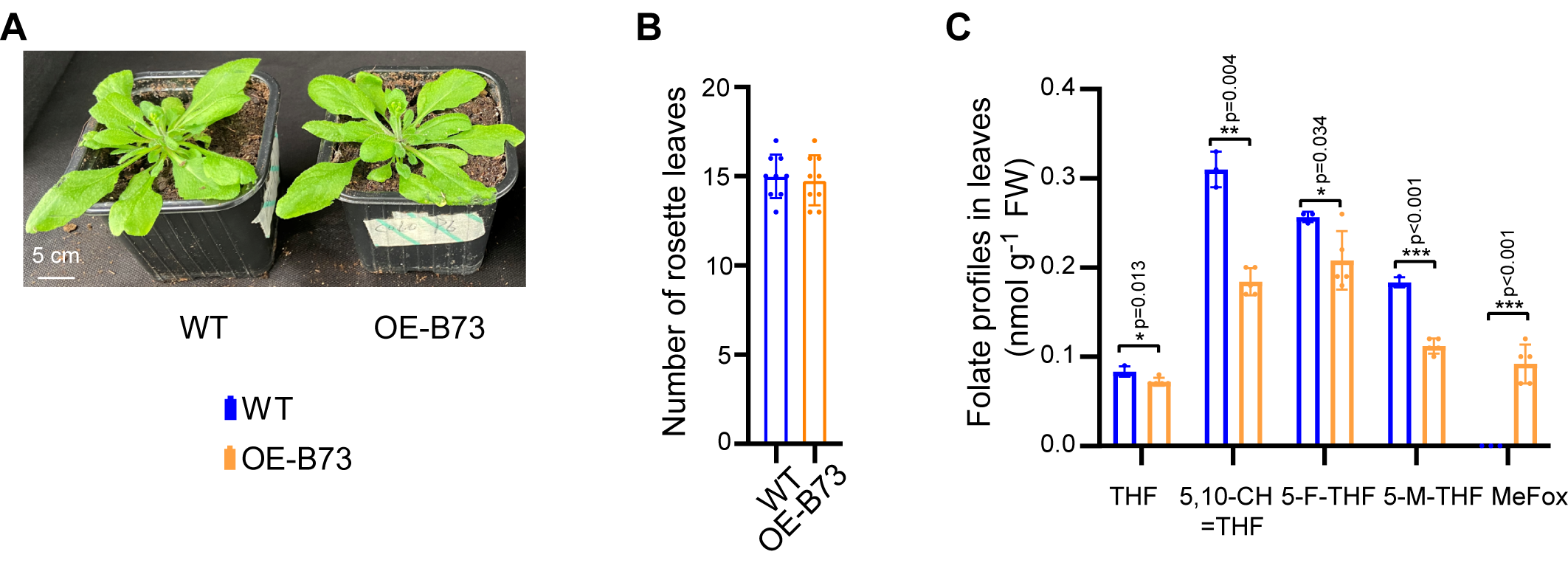

Supplement: Supplementary file 14 — Supplemental Figure 13 [file ADVS-12-e15082-s006.tif]

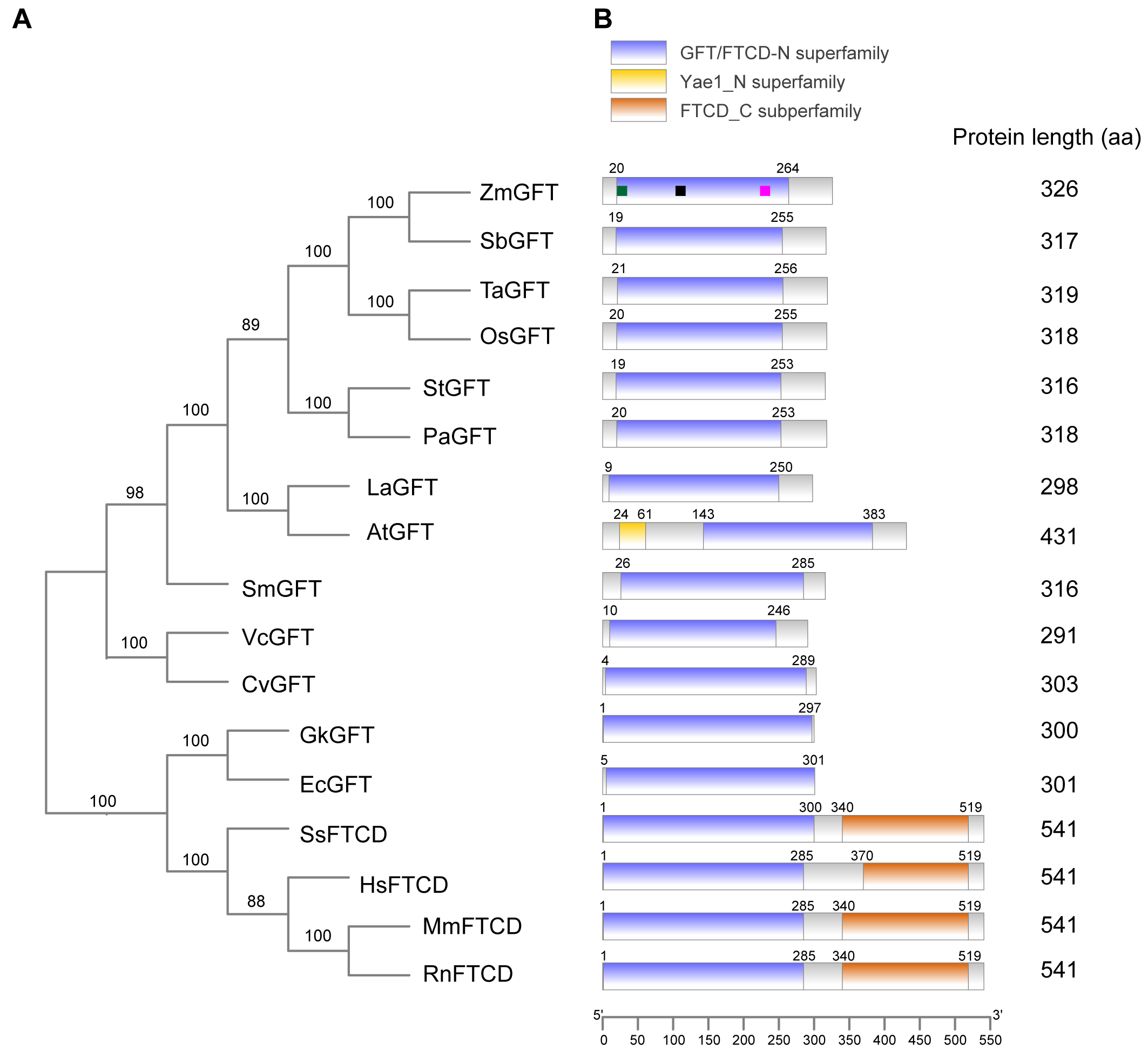

Supplement: Supplementary file 15 — Supplemental Figure 14 [file ADVS-12-e15082-s003.tif]

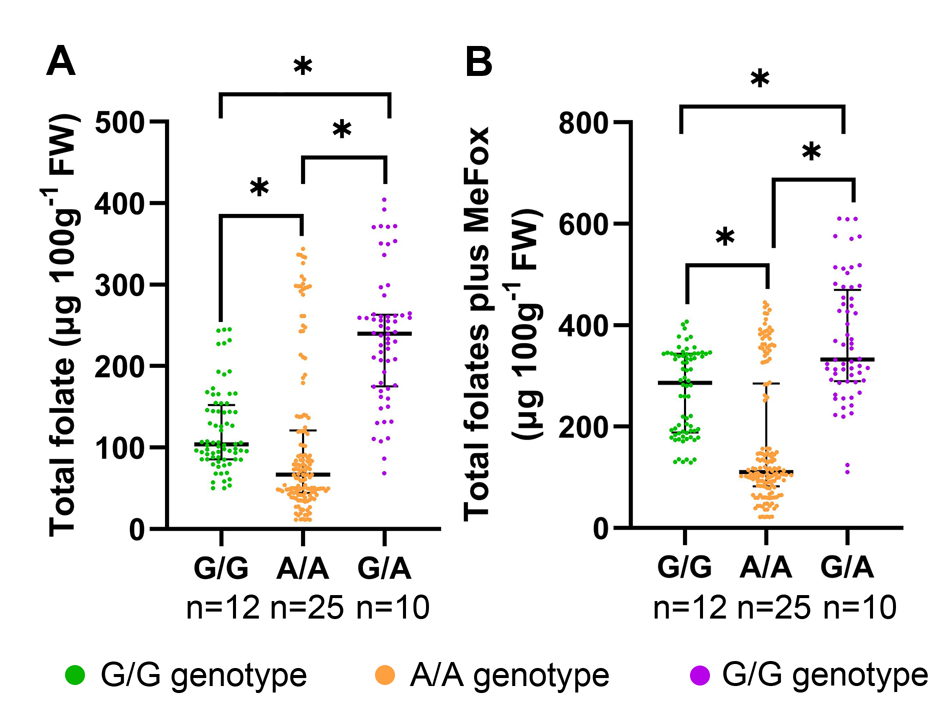

Supplement: Supplementary file 16 — Supplemental Figure 15 [file ADVS-12-e15082-s011.tif]

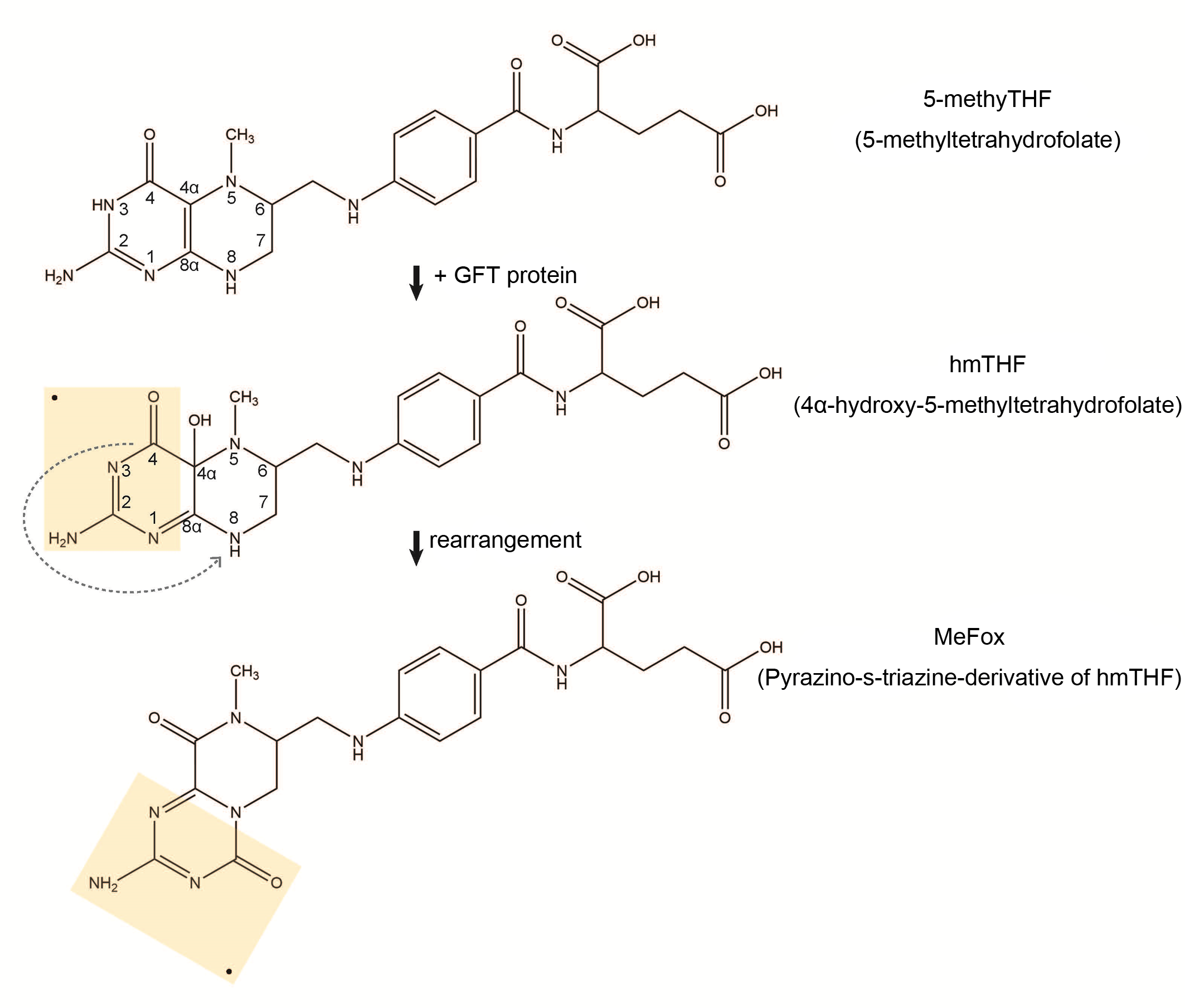

Supplement: Supplementary file 17 — Supplemental Figure 16 [file ADVS-12-e15082-s033.tif]

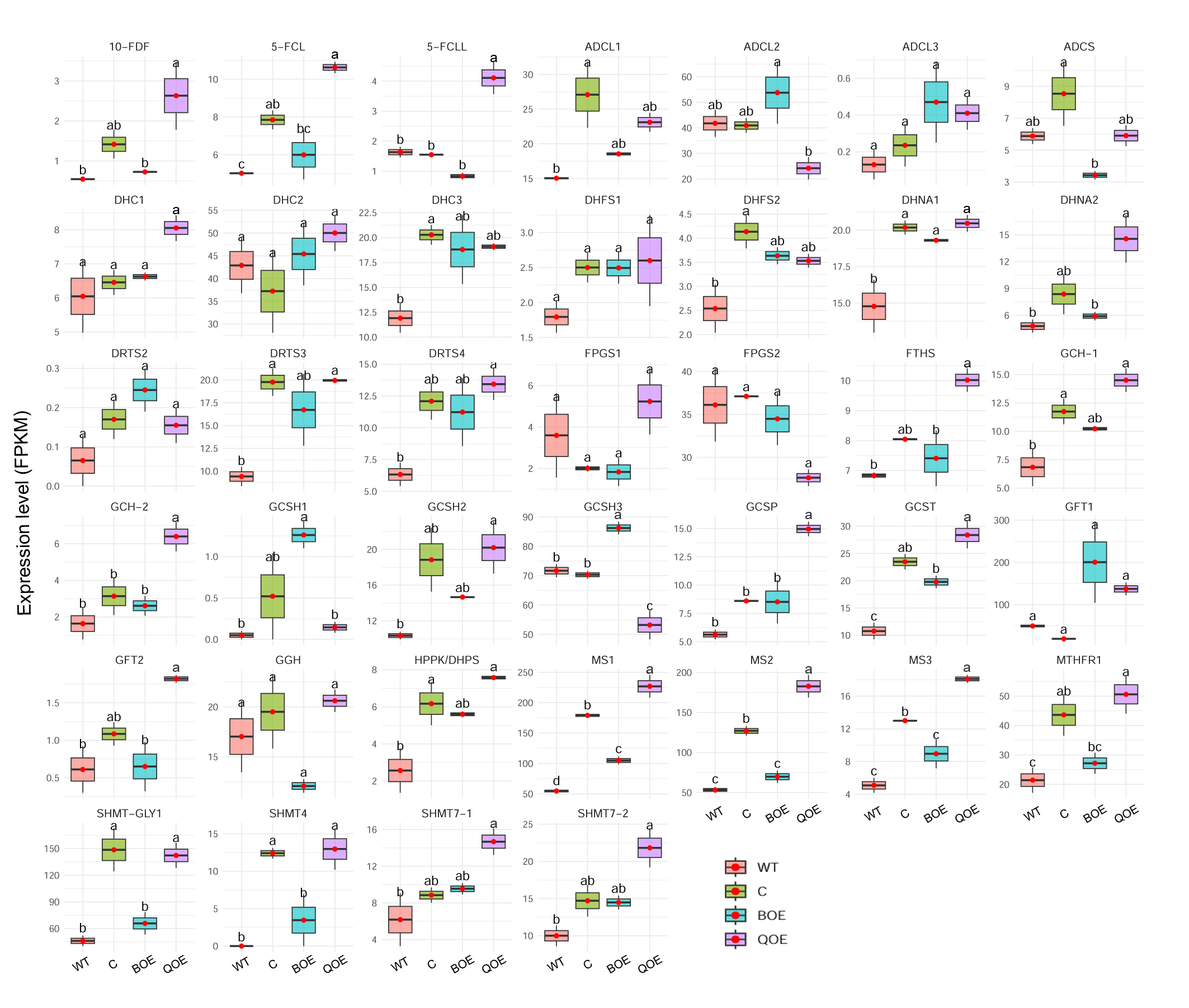

Supplement: Supplementary file 18 — Supplemental Figure 17 [file ADVS-12-e15082-s030.tif]

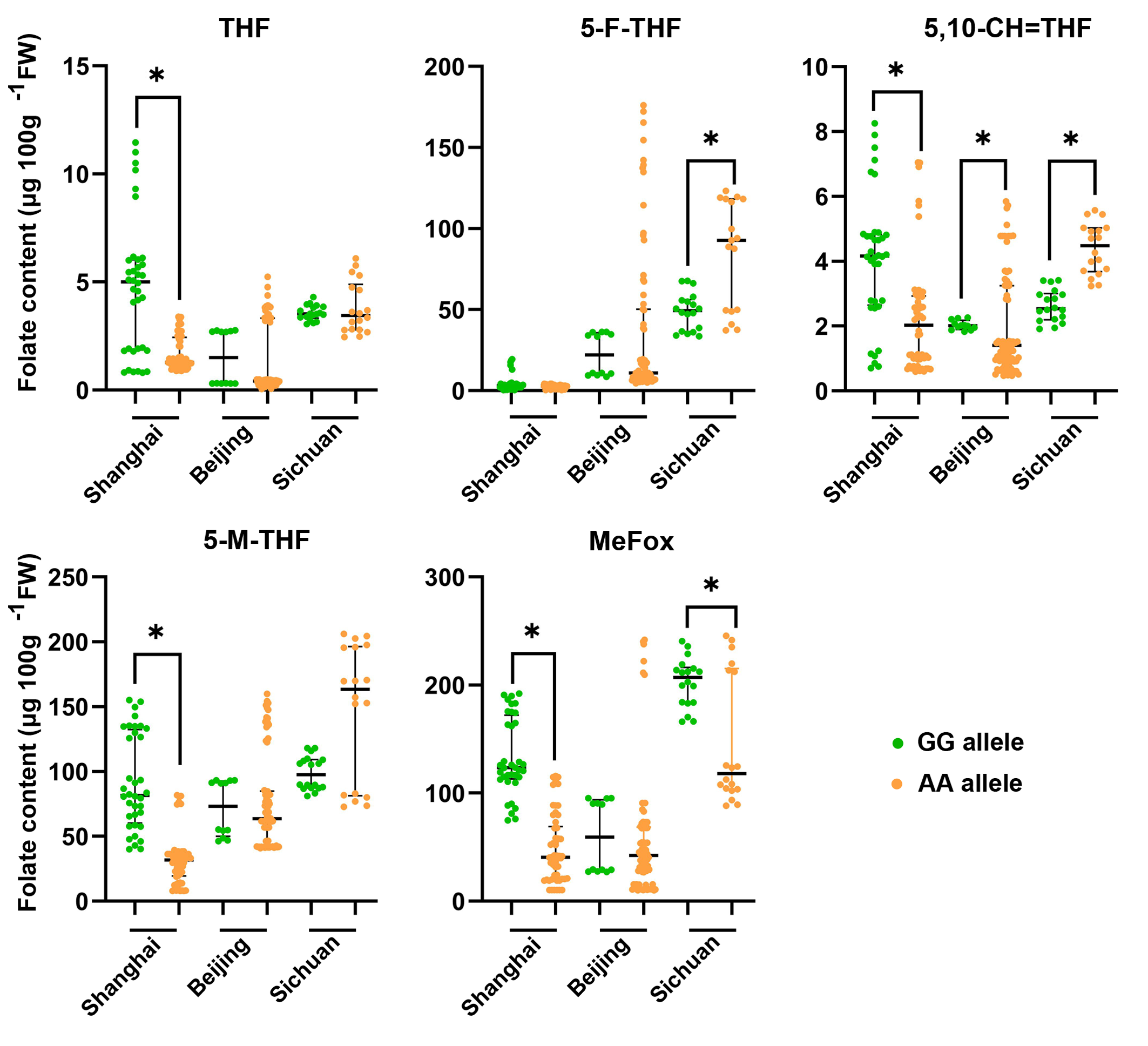

Supplement: Supplementary file 19 — Supplemental Figure 18 [file ADVS-12-e15082-s026.tif]
